# Very short-term production prediction for photovoltaic plants using Temporal Convolutional Networks

**DOI:** 10.1371/journal.pone.0354512

**Published:** 2026-07-28

**Authors:** Loukas Samaras, Elena García-Barriocanal, Miguel-Angel Sicilia, Lino González García

**Affiliations:** 1 Computer Science Department, Polytechnic Building, University of Alcalá, Madrid, Spain; 2 Artificial Intelligence (AI) Consulting and Solutions, Madrid, Spain; Dr Shakuntala Misra National Rehabilitation University, INDIA

## Abstract

Very short-term forecasting of solar photovoltaic energy production at national scale is challenging due to the high variability and spatial aggregation of generation across large territories. This paper evaluates Temporal Convolutional Networks (TCN) — a deep learning architecture based on causal and dilated convolutions — for nowcasting national-level solar production at one-hour and fifteen-minute horizons, using data from Spain sourced from the European Network of Transmission System Operators for Electricity. Multivariate models augmented with past weather observations (solar irradiance and sun height) are compared against linear regression baselines. Results demonstrate that the multivariate TCN substantially outperforms linear regression at the one-hour horizon, and achieves consistent improvement at the fifteen-minute horizon. The relative contribution of architecture and weather features is resolution-dependent: at hourly granularity, the TCN architecture itself provides the dominant gain, while at the fifteen-minute scale the inclusion of weather covariates becomes the primary driver of accuracy, reflecting the greater atmospheric variability at finer temporal scales. A key finding is that past-only weather inputs are sufficient for accurate nowcasting, eliminating the need for future meteorological forecasts as model inputs. The results support the practical applicability of TCN-based models for national-level solar energy integration and provide a data-driven feature-selection criterion for similar renewable energy forecasting tasks.

## 1. Introduction

From 1974, the global energy demand grew from around 5,000 TWh to almost 22,000 in 2019, four and a half times more, with an average annual increase of 3.32% [1–3]. This growth can be approximately expressed by the following equation:


$$\begin{array}{c} \text{Ed }=\text{ 5388}.\text{9}{\text{e}}^{\text{0}.\text{032x}} \end{array}$$ (1)

where *Ed* is the energy demand and x stands for the year factor = *y* – 1973, with *y=* {1974, 1975,... 2019}.

**Fig 1:**
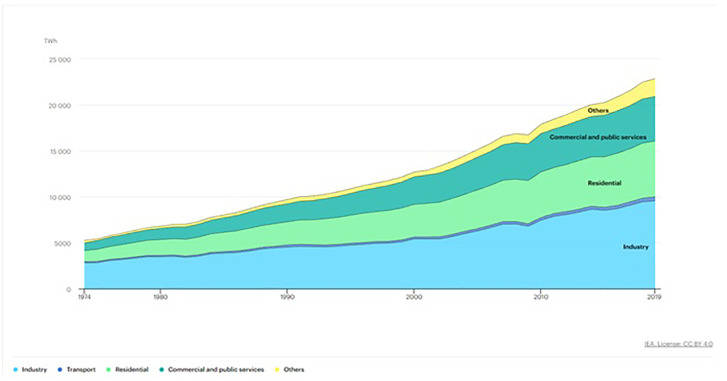
The global energy consumption (TWh) 1974-2019 (image by IEA).

Although the International Energy Agency (IEA) reported that, during 2019, the total power consumption increase was reduced by around 1.1% in OECD countries, the rise in the non-OECD countries was much higher, specifically 3.8% in the previous year. Furthermore, according to the above equation, global consumption is estimated to double over the next twenty years to around 50,000 TWh. Fig 1 shows the global energy consumption, total and by sector:

Meanwhile, the world population has not increased at the same rate, as it almost doubled from 4 billion in 1974 to almost 7.7 billion in 2019 with a continuously declining annual rate (from 1.94% in 1974 to 1.05% in 2019) [4]. Despite the declining rate of population increase, some sectors, such as industry, absorb so much energy, amounting to around three-quarters of the total consumption (74.08%) [5]. The updated global electricity demand, according to IEA’s 2024 report, is expected to rise at a faster rate over the next three years, growing by an average of 3.4% annually through 2026 [3].

It seems that the dominant reasons for this high energy demand could also be found in the financial growth and the income development of the world population [6]. In any case, improving energy efficiency and energy production may be the key to meeting the world’s high energy demand. This, combined with using more environmentally friendly energy sources, such as photovoltaic or wind energy, would allow tackling global climate change. Therefore, an accurate forecast can be considered of great importance for achieving the proper balance of energy supply for the years to come. During the last decade, an increasing number of researchers have approached the challenge of developing and extending ideas on ‘strategic niche management’. These ideas include developing desirable sustainable innovations by experimenting with new technologies and socio-technical systems to achieve more sustainable development. In the energy domain, this discussion encompasses the importance of shared long-term objectives, the necessity of harmonizing energy policies, and the requirement for continual monitoring and adaptation to energy and climate changes [7]. Prediction, on the other hand, is needed both in the short term and in the medium or long term to maintain the optimal balance of power production and demand, as state*d in* [8]*: “The optimal investment and operations of integrated local energy systems require medium to long-term prediction of energy consumption*”.

Solar energy is one of the cleanest sources of energy and one of the most abundant, with over 63 million PV systems, heat pumps and EV chargers already installed in European homes. Its magnitude is globally estimated 1,017 joules, which can be delivered per second. In contrast, the oil reserve in the total world is estimated to be 1.7*1022 joules [9]. Furthermore, it has been found that an accurate prediction of the energy load can result in an increase in the efficiency of solar Photovoltaic Cells (PV), e.g., the Maximum Power Point Tracking algorithm (MPPT) [10]. In a recent study of 2023 [11], modern artificial neural networks are proposed to forecast a solar PV System for a standalone household system. This study aimed to achieve a 24-hour ahead forecast of the energy load of a standalone system to prepare the battery for future use. The forecasting uses machine learning techniques and deep learning networks like RNN, LSTM, and Gated Recurrent Units (GRU) to predict the weather, the solar irradiance and the temperature.

ENTSO-E is the European Network of Transmission System Operators for Electricity, the association for the cooperation of European Transmission System Operators (TSOs). It has 39 TSO members and represents 35 countries responsible for the secure and coordinated operation of the European electricity system, the largest interconnected electrical grid in the world. These operators are responsible for safe and efficient electricity production and/or distribution. By monitoring and maintaining equipment and this system, they can ensure they operate at peak efficiency. The network offers its data online, including data on electricity production from renewable resources, such as solar and wind farms, onshore and offshore. In addition to the hourly power produced (changed to a 15-minute window from mid-2022 for most countries), it also provides production or consumption forecast data for the next day. According to the ENTSO-E demand methodology [12], the prediction system utilizes a temperature regression and a load projection model that incorporates an analysis under various climate conditions. More specifically, it uses a regression algorithm with input variables split into two profiles and one parameter profile that includes a) electricity load data (training years 2012–2016), b) climate variable profiles (e.g., city temperature, irradiance, wind speed and holidays for the same training period) and c) parameters for the regression settings (e.g., order, p-value), for days groups and pod functions. Finally, all these variables are adjusted by applying different coefficients for temperature dependence and independent loads to produce the final prediction model.

**Fig 2:**
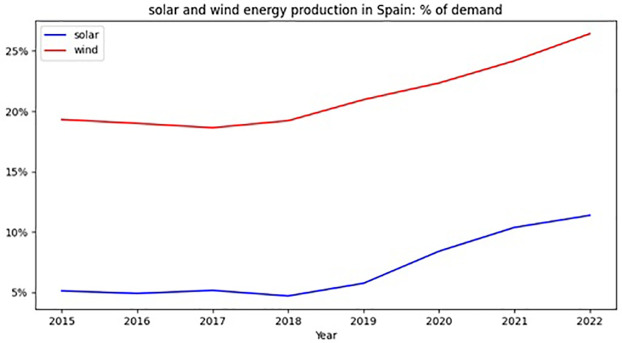
Production of solar and wind energy in Spain (2015-2022). Source: ENTSO-E [10,12].

A short or mid-term estimation will be helpful in order to achieve the power balance (of production and demand) and take advantage of the novel prediction models and methods, especially in the field of renewable energy generation based on DL techniques and domain knowledge [13]. For instance, some models estimate the energy consumption of buildings based on specific parameters, such as the power load density, the area, the heat gain, the outdoor air, the existence of internal or external walls or doors, etc. [14]. As noted above, it is not only residential energy consumption that has increased over the last decades but also the power draw related to other activities, such as industrial operations. It is crucial to bear in mind that, at least for now, the energy generation from renewable resources, such as wind farms, cannot cover a large portion of the demand. In the case of our study, the daily average of the energy production by the wind industry is limited, and for Spain, it is only 168,473.3 (kilo Watts by hour) KWh, less than 170 MWh daily. Nevertheless, the constantly increasing global demand, along with climate change and the need for sustainable growth with the careless protection of the natural ecosystem, may result in a generalized and extended use of these alternative resources. On the other hand, current ENTSO-E data show that solar energy production in Spain is even more limited and covered only 5.07% of the total energy load until 2018. However, after 2022, this had doubled to over 11% of the total demand, while the wind plants produced 19.10% until 2018 and increased to 26.42% of the total demand in 2022, as shown in the following Fig 2. This means that energy production from PV cells still falls short of the energy produced by wind plants.

In recent years, empirical tests have revealed that Convolutional Networks, such as the Temporal Convolutional Networks (TCN), substantially outperform generic recurrent architectures such as LSTMs and GRUs, while they can provide certain benefits, such as flexible receptive field size, stable gradients, low memory requirement for training and variable length inputs [15] Furthermore, Nvidia Corporation has been using convolutional neural networks [16] for years in their graphics cards to manage upscaling of images and video and finally increase the efficiency of this hardware. Therefore, it could be of particular interest to apply such models for energy forecasting.

In the recent work by Li et al. (2023) [17], the TCN model was found to provide a significantly better forecast of the solar energy from PV cells from 95 utility scale PV farms in North Carolina, USA, from January 1 to November 30, 2020 over other models, such as the linear regression or even the LSTM neural networks.

Furthermore, the research has been extended to evaluate solar energy production with external weather data and artificial neural networks, such as in the research on the solar PV power output for 250MW solar farms by G. Balraj, et al. (2022) [18], or by Zameer A, et al. (2023) [19]. However, current literature provides studies on limited-area forecasts with specific power plants located there. On broader areas, such as on a national level, the work of T. Cabello-López et al. (2023) [20], focuses on the entire Spanish territory, consisting of hundreds of solar farms. The main goal of the latter was to develop a methodology to integrate solar irradiance forecasts with historical data from solar power plants in Spain to improve the performance of multivariate models, including neural networks.

Given that the prediction of a standalone power system is feasible and more accessible because it is studied in a specific location with specific weather conditions, as in the work of [11], it is interesting to investigate the feasibility of forecasting the solar energy production of an entire country, such as Spain, which consists of multiple power stations and several households and enterprises in different areas, and therefore with different weather conditions. This context raises the following challenges:

- The high volatility of the data, e.g., the variance or standard deviation, which is much higher than the energy load.

- The aggregated values of solar energy production of all plants in Spain.

- The different climatic conditions of each power plant across the country.

**Fig 3:**
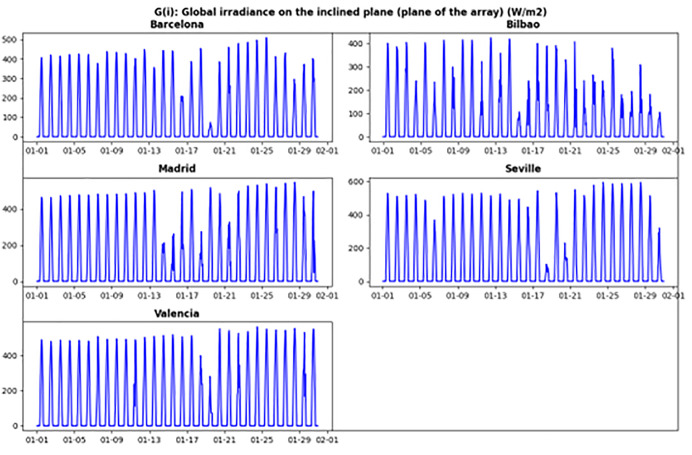
Global irradiance in the most significant areas of Spain (January 2015).

The solar irradiation, for example, varies across different parts of Spain, as shown in the following Fig 3, which depicts the global irradiance in the five major areas of this country:

Existing national-level solar energy forecasting for Spain has been addressed by Cabello-López et al., who apply a multivariate CNN-LSTM to System Operator Information System (E-SIOS) data across 1-hour, 24-hour, and 48-hour horizons, outperforming the official system operator forecast by 47.58% for the 1-hour case. However, their study does not address sub-hourly horizons, which are critical for intra-day electricity market operations. On the methodological side, Li et al. [17] demonstrate that TCN architectures outperform CNN-LSTM and other deep learning models for utility-scale PV forecasting on individual farms. The present work bridges these two directions: it applies TCN to the national-level Spain forecasting problem at the 15-minute resolution — a horizon not addressed by prior national-level studies — reporting nMAE values of 4.10% (1-hour) and 1.79% (15-minute) for the multivariate model.

Given the above, taking into account the existing literature and the aforementioned challenges, we expand the current research with more data by setting the following research questions:

- RQ1: Is it possible to predict the solar energy production of a whole country consisting of many power production units?

- RQ2: In this scenario, is the efficiency of forecasting with AI algorithms much higher than that of traditional methods, e.g., linear regression?

- RQ3: Can weather data further improve forecasting?

- RQ4: If RQ3 is true, is it needed first to forecast weather data for a short-term prediction?

The ultimate goal of the research presented in this paper is to produce short or very short-term forecasts of solar energy production based on one-hour and fifteen-minute data for a multi-stakeholder country. To this end, six different models are presented in this study, three for one-hour data and three for fifteen-minute data, and all of them are compared.

The current paper is structured in four sections. This Introduction contextualizes the problem and reviews related work. Section 2 (Materials and Methods) describes the data, models, and evaluation metrics. Section 3 (Results and Discussion) presents and interprets the experimental findings. Finally, Section 4 presents the Conclusions.

## 2. Materials and Methods

**Fig 4:**
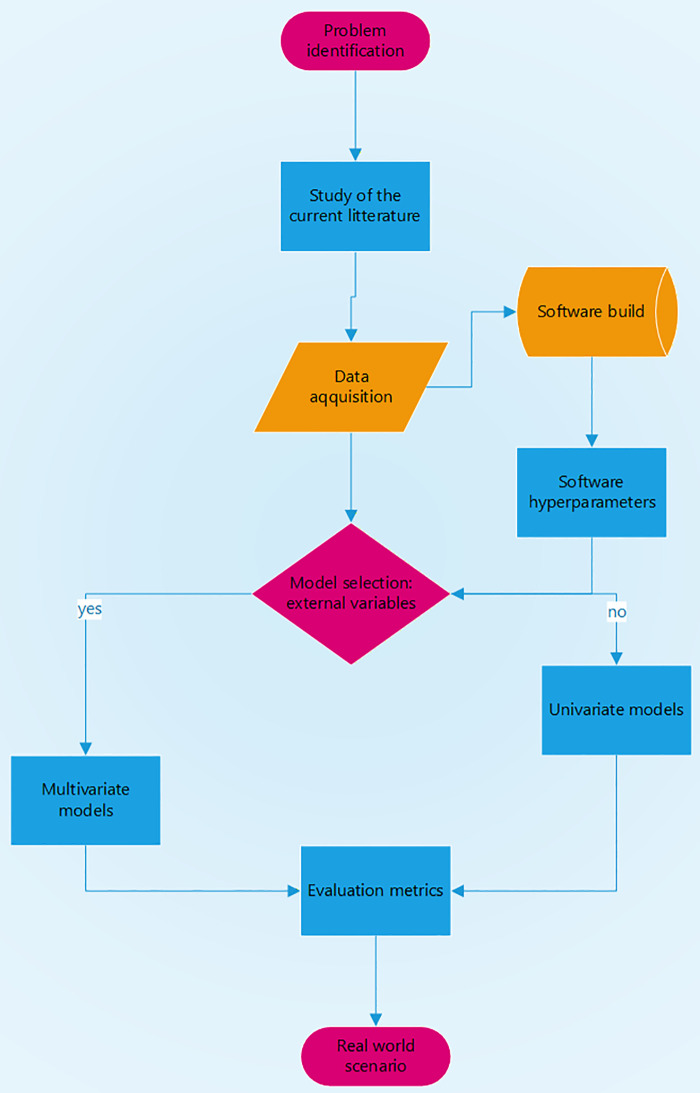
The flowchart of the research.

This research has been conducted according to the following chart flow, as shown in the following Fig 4:

The first two steps (the problem identification and the study of the current literature) were presented in the preceding sections. The analysis from now on will focus on the following:

• The data acquisition (energy and weather data). All datasets we used have been uploaded to a GitHub repository (https://github.com/luxorsatellite/Darts_tcn/tree/main)
• The software builds with the best optimizations
• The model selection and the inclusion of exogenous variables
• Training univariate and multivariate models with 80% of the data and
• backtesting the remaining 20% and evaluating models’ performance and finally,
• A real-world scenario, in which we predict the future of the solar energy production out of the sample, meaning after the end of the dataset. In this scenario, we present, not only the outcome, but also the computer hardware resources and total time needed for this. For this scenario, there are no separate training and validation sets; all data are trained in order to achieve out-of-the-sample predictions. When training on the full dataset, the best model was saved and loaded to provide the best forecast out-of-the sample.

### 2.1. Energy data

The power data represent the aggregated solar generation in Mega Watts (MW) and are the energy production generated by the solar PV cells from solar stations all over Spain. The ENTSO-E system has available hourly data from 2015-01-01 until 2022-05-22, while afterwards, the data are published at a 15-minute resolution. Until 2018, solar energy production was 5.07% of the total power demand; during 2022 only, it was over doubled to 11.38%. For this study, two datasets come from the ENTSO-E system:

All energy and weather data can be found on the Kaggle website [21]. Solar energy production data can also be found in the python darts dataset’s library and can be directly downloaded by writing the python command “series = EnergyDataset ().load()”. Solar energy data include the following:

i) hourly data extending from 01.01.2015 until 31.12.2018 (4 years). The mean value of these data is 1,432.82 MW and the standard deviation is 1,679.96 MW.
ii) 15-minute data from 23.05.2022 until 31.12.2023 (2 years) with a mean value of 4,319.52 MW and a standard deviation of 5,263.24 MW, over the mean.

As already mentioned in the Introduction, solar energy production has a standard deviation of over 100% of its mean, while for the energy total load (consumption) from all over Spain (years 2015–2022), the mean is 28,268.69 MW per hour and the standard deviation is only 16.51%, 4,565.86 MW/h.

In this research, the first four-year dataset is combined into a single series with weather data of the same period. The second one, covering almost two years, starting from 23.05.2022, has been used in combination with solar irradiation of the same period, as there are no 15-minute solar irradiation data before. No more data of this kind (hourly or 15-minute) exist after 2023.

**Fig 5:**
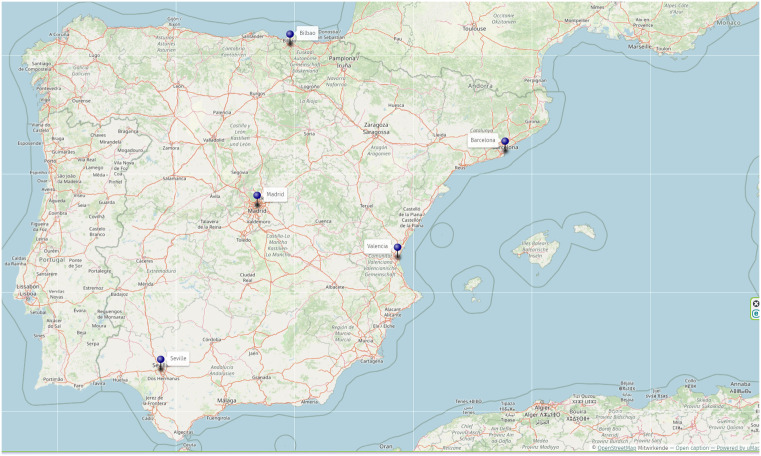
The most significant areas of Spain.

Data are provided from solar PV cells located in cities that represent the major areas of Spain, from approximately the four cardinal directions: North, South, East and West, as shown in the following Fig 5:

As mentioned in the Introduction as a challenge, it should be considered that these values are aggregated at country level, and their standard deviation is much higher than the energy consumption data. Therefore, the power demand is more predictable since it drops after 23:00 almost daily. However, solar energy production significantly drops after the sun sets, which is not always the same for each day and season of the year. The above considerations result in a significant forecast loss, even hourly.

**Fig 6:**
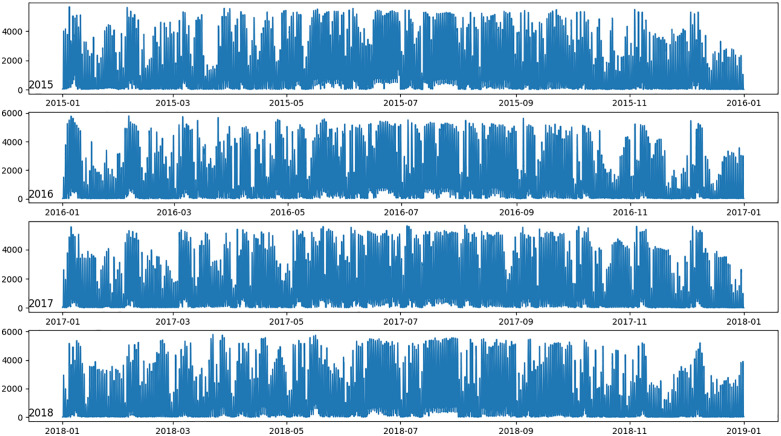
The decomposition of solar energy.

Finally, it should be noted that although power demand increases in summer and winter, solar energy production is affected not only by the current month, solar radiation and cloud coverage but also by the season, as shown in the following Fig 6:

Fig 6 shows that the maximum (aggregated) production can reach its maximum capacity even in winter (e.g., in January of 2016, almost 5,800 MW) despite lower temperatures and significantly less cloud cover. This happens because the heat loss is lower, so the energy loss, although it cannot be sustained for as long as during summer. One type of loss in PV systems is thermal loss: the more heat, the more loss, and, thus, the less power. This is why, even in winter, the energy production from PV can reach a similar peak production capacity as in summer months, although for shorter time, as the sun shines less.

### 2.2. Weather data

For the purpose of this research, we compare univariate models with only energy data and models with weather data as explanatory variables of two datasets: one with hourly values and another one with values every 15 minutes.

These data are gathered from the following sources:

i) Weather Hourly data from Open Weather Application Program Interface (API) [22] for the five largest cities in Spain: Bilbao, Barcelona, Madrid, Seville and Valencia.

These data include temperature (average, minimum and maximum, pressure, humidity, wind speed, wind direction (degrees), one- and three-hour rainfall and three-hour snowfall, as well as cloud sky coverage data,

ii) Weather data from the European Commission Photovoltaic Geographical Information System (PVGIS) [23] website for the same five largest cities, which are available through the PVGIS-ERA5 system and the radiation database PVGIS-SARAH2. This dataset includes the following data:

- Global irradiance on the inclined plane (plane of the array), in W/m2

- Sun height, in degrees

- 2 m. air temperature, in Celsius degrees

- 10 m. total wind speed, in m/s

### 2.3. Past and future variables

External variables (called ‘covariates’ in Python Darts) can be used to predict each target variable in two ways:

i) As future unknown: In the case of solar energy production, we could predict the target variables (energy values) based on our knowledge of the future exogenous variables, such as the irradiance data in this case. For instance, if we have target energy data for January of a specific year and want to predict February of this year, we definitely need to know the weather data for February in advance.

The problem, however, is twofold:

First, we don’t actually know the future irradiance data. One solution would be to first conduct a forecast of them and then insert them as future inputs into the target data and make a second forecast of the target, but this way, the forecast results of the target data will be lowered, because two forecast procedures would be executed; one for the external data and another for the target data. Since it is impossible for the first ones to reveal a correlation of 100%, the second forecast will inevitably be less accurate. This is mostly done in the current research, as depicted in the current literature.

Second, most models (e.g., the Auto-Regression Moving Average model with exogenous variables-ARIMAX, the TCN model, etc.) do not accept future covariates, but only past covariates.

ii) As past known: in this case, external data are treated as past covariates, meaning the same way as the target data. This way, we save time and accuracy, as we do not have to perform two forecasts.

### 2.4. Datasets and Missing data

The first dataset of one-hour values contains no missing data but in the second one there nine missing energy values. To deal with this, we use linear interpolation to fill in the nan values with the following python command: *df = df.resample(resamplimg_interval).interpolate(limit_direction = ‘both’), where:*

• df is the dataframe,
• resampling_interval = 15 minutes
• *limit_direction* = interpolation direction: both, meaning forward and backward

### 2.5. Model selection

This subsection describes different models designed and developed to predict solar energy production. Implementations of all models were developed within Python version 3.11 [24] environment with Darts version 0.29 [25]. All models were trained with a modern computer system of a CPU with 24 cores and 32 threads (i9-13980HX) and a powerful graphics card (GPU) consisting of 7,144 Cuda cores (Nvidia RTX 4080M) with a compute capability of 8.9.

Models and their architecture were progressively enriched in an attempt to improve results. Initially, a multivariate linear regression model with only the energy data was taken as a baseline, then a univariate TCN and finally, a multivariate TCN model with weather features was tested. All of them were tested for both hourly and 15-minute data. In total, six models were evaluated, three with the hourly data and three with the 15-minute data.

The baseline model serves as a fast and reasonably precise estimate for the current dataset, in order to be compared to more complex models later on. Since we use the python darts library, we could use as baseline any classic model, such as ARIMA, naïve model, exponential smoothing, etc. We opted for linear regression, because it can support both past and future covariates, datetime cyclical features and it is very fast compared to other models, e.g., ARIMA, for datasets of over 35,000 values. The ARIMA model, for example, for back testing estimation, needs around three hours in the first 35K+ dataset, while the second 56K+ dataset took over 9 hours, because it is executed on a CPU single-thread instance, while the linear regression is executed on multi-thread instance.

The datasets were divided into training sets of 80% of all the values, while the remaining 20% was left as a validation set. It should be noted that the Python Darts library, when backtesting, does not allow different training, validation and test sizes as in traditional neural networks without this library (e.g., 80% training, 10% validation and 10% test size), but only training and validation datasets. This means that the training is done with the training data and the best-found model is directly applied to the validation dataset.

The entered weather data were selected according to their correlation to the energy values. The weather variables used are as follows:

• The G(i), which means the global irradiance on the inclined plane, showed an average correlation coefficient of 0.796 with the energy data of one hour and much lower, 0.548 for the 15-minute data.
• The H_sun, meaning the sun height (degree), with an average correlation of 0.785 and 0.552 respectively.
• The temperature has an average correlation with solar energy of only 0.369 and a very low and negative correlation of −0.0452 respectively.

**Fig 7:**
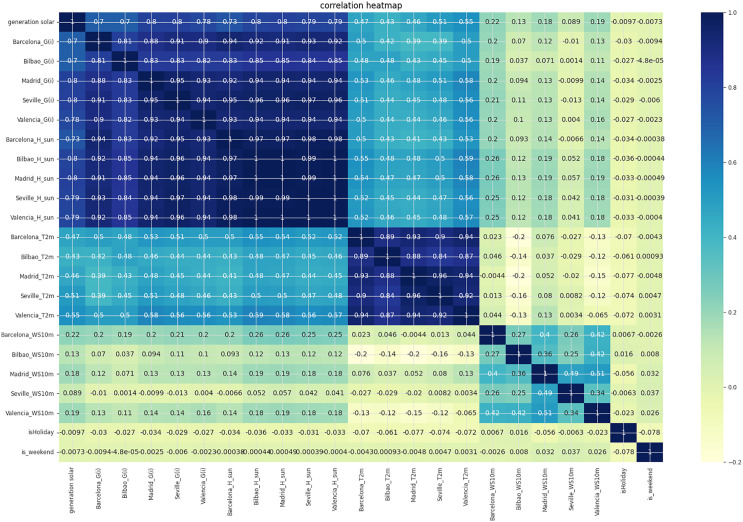
The correlation heatmap.

The feature selection was based on the best correlation coefficients between energy and weather values. The above Fig 7 shows the respective correlation matrix, while Table 1 shows these correlations, both of the entire data and the training data only.

**Table 1:** Best correlations.

Dataset	variable	name	R	R (train)
**hourly**	**0**	generation solar	1	1
**“**	**8**	Madrid_H_sun	0.804021	0.799143
**“**	**4**	Seville_G(i)	0.803871	0.800096
**“**	**3**	Madrid_G(i)	0.800787	0.796690
**15-minute**	**17**	generation solar	1	1
**“**	**29**	Seville_H_sun	0.772861	0.776642
**“**	**24**	Seville_G(i)	0.769252	0.766909
**“**	**28**	Madrid_H_sun	0.750504	0.755526

### 2.6. Neural network: the TCN model

Generally, a convolutional equation h is the result of a mathematical operation on two (or more) functions f and g. Given t is the time, it is estimated as follows:


$$\begin{array}{c} h=f*g(t):=\int_{-\infty }^{\infty }f(\tau )\cdot g(t-\tau )\cdot d\tau \end{array}$$ (2)

where:

**h**: the integral of the product of two functions *f* and *g*

**t** and **τ,** time or numerical spots (e.g., 1,2.3…n). It is the area under the function f(τ), weighted by the function **g(τ)** and shifted by the amount t, where:

**f(τ)** and **g(τ)** are the input functions.

TCN employs causal convolutions to ensure that outputs at each time step depend only on past inputs, eliminating any risk of information leakage from the future. A causal convolution layer computes:


$$\begin{array}{c} y_{t}=\sum_{i=\text{0}}^{k-\text{1}}f(i)\cdot x_{t-i} \end{array}$$ (3)

where:

y_t, the output at time t

x_{t − i}, the input at lag i (only past and present values are used)

f(i), the filter coefficient at position i

k, the filter size (kernel size)

The causal constraint ensures that y_t depends only on x_t, x_{t − 1}, …, x_{t − k + 1}, with no access to future inputs.

Dilated causal convolutions extend Equation (3) by introducing a dilation factor d, which creates gaps between filter taps and allows the network to capture exponentially larger temporal contexts without increasing the filter size. The dilation factor is typically set to d = 2^1^ at layer l, so that a network of L layers with filter size k achieves a receptive field of 1 + 2·(k − 1)·(2ᴸ − 1). The specific values of k, L, and the dilation base used in this work were determined by hyperparameter optimisation and are reported in Section 2.6. The dilated causal convolution operation is:


$$\begin{array}{c} F(s)=(x\cdot df(s))=\sum k=\text{1}f(i)\cdot x_{s-d\cdot i} \end{array}$$ (4)

For a 1-D sequence input x ∈ ℝ and a filter f: {0... k-1} -- > ℝ. Note that, for a given sequence length, the maximum input sequence must not exceed the total validation size minus the output length.

**F**, the dilation operation

**s**, the element of the sequence

**d**, the dilation factor,

**k**, the filter size

s-d*i stands for the direction of the past. When d = 1, a dilated convolution reduces to a regular convolution.

**Fig 8:**
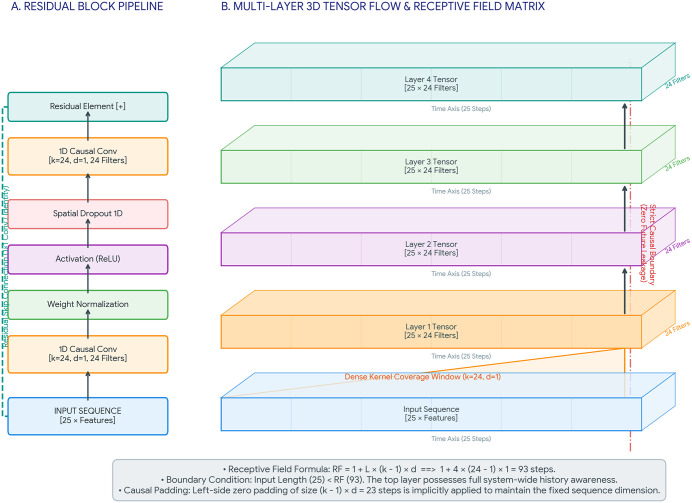
TCN architecture.

Residual connections allow the TCN to be trained as a very deep network by letting gradients bypass convolutional layers. Each TCN residual block computes:


$$\begin{array}{c} o=ReLU\left(x+F(x)\right) \end{array}$$ (5)

where:

F(x), the dilated causal convolution transformation of the block input

x, the block input; a 1 × 1 convolution is applied to x when input and output dimensions differ. In the following Fig 8 a description of the TCN model architecture and its main components is depicted:

**A. Residual Block Pipeline (Block Architecture):**

The left section of the diagram illustrates the internal processing flow of a standard TCN Residual Block. The data passes sequentially through two stages of 1D Causal Convolutions, each paired with Weight Normalization to stabilize training, a ReLU activation function to introduce non-linearity, and Spatial Dropout to prevent overfitting. The dashed green line represents the Residual Skip Connection, which adds the original input directly back to the final transformed output, enabling efficient deep network training by eliminating the vanishing gradient problem.

**B. Multi-Layer 3D Tensor Flow (Three-Dimensional Data Stream):**

The right section geometrically maps out the data dimensions as they move upward through the 4 stacked hidden layers. Each layer is visualized as a 3D tensor block to demonstrate how 24 independent filters extract features concurrently across all 25 timesteps of the sequence. The shaded orange area at the base highlights the dense kernel operation where k=24 and dilation d=1; a single hidden node compiles a continuous historical window of 24 consecutive steps without skipping any entries, while the vertical red dashed line (Causal Boundary) guarantees that no future data leaks backward in time.

**C. Receptive Field Formula (Mathematical Foundation):**

The card at the bottom explains the global system awareness using the Receptive Field equation: RF = 1 + L times (k - 1 times d). Given 4 layers (L=4), a massive kernel size (k=24), and a fixed dilation (d=1), the model builds a maximum contextual reach of 93 past timesteps. Because the actual input sequence length of 25 is well within this 93-step coverage window, the final top layer possesses full historical memory of the entire sequence from the very first step, utilizing an implicit left-side causal zero padding of 23 steps to keep tensor dimensions perfectly uniform throughout the pipeline.

The general TCN framework supports dilated causal convolutions. Nevertheless, the optimized configuration selected in this study used a dilation base of 1. Therefore, the final implemented model corresponds to a causal convolutional TCN residual architecture without dilation expansion.

### 2.7. Model optimizations

One of the more difficult tasks in neural networks is to determine the appropriate parameters of the selected model. To achieve this, we used the following methods provided by the Python Darts library, which are not available in other frameworks, as described below:

**Seasonality check:** Statistical seasonality testing identified seasonality periods of twenty-four hours, nine days, four weeks, and twelve months; these were used to determine the minimum kernel size and number of filters.

**Encoders (datetime attributes):** Cyclic encodings of datetime attributes were used as past covariates. For the 1-hour dataset these comprised month, week, day, day-of-week, and hour; for the 15-minute dataset, minute was additionally included. All encoded features were normalised using a MinMax scaler.

### Optimization libraries-Optuna

There are two popular Python libraries for hyperparameter optimization, named Optuna [26] and Ray Tune [27].

We used the open-source *Optuna* library for the hyperparameter optimization, and the Darts’ TCN Model, and found that the best layers are four, while the best dilation base parameter was 1, meaning a regular convolution process will be executed, as by the abovementioned equation 4.

### Learning rate tuner

An automated learning rate finder was applied before training, scanning candidate values and selecting the one associated with the steepest loss decrease.

**Fig 9:**
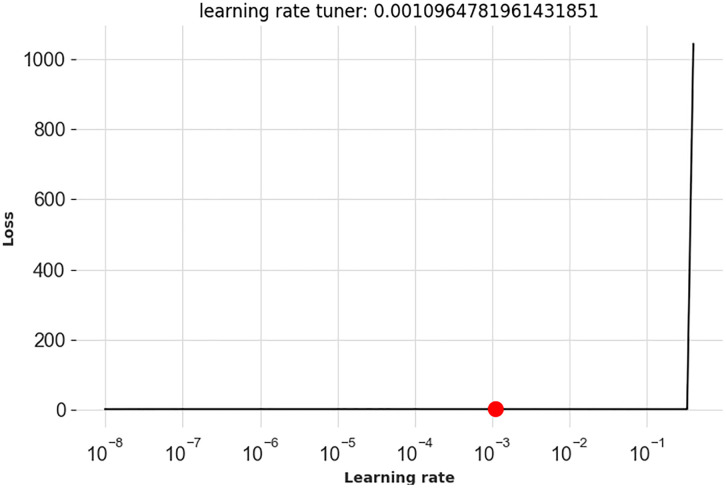
The learning rate tuner (TCN).

For example, given a lr_decay = 0, the best learning rate for the best TCN network (forecast horizon 1h) was found to be ≈ 0.0011, as shown in Fig 9.

**Scaling:** Data were normalised to the [0, 1] range using a MinMax scaler, which speeds up training and reduces RAM and VRAM usage.

Neural networks were trained using the PyTorch library [28] with Nadam as the optimiser, together with a ReduceLROnPlateau learning rate scheduler and early stopping monitoring validation loss.

The hyperparameters of the best model are: input length 25 (seasonality of 24 plus forecast horizon of 1), batch size 24, kernel size 24, dilation base 1, maximum 100 training epochs with early stopping patience of 20 epochs, dropout 0.2, weight normalisation enabled, 4 layers, and weight decay 0. A fixed random seed was used throughout to ensure reproducibility.

### 2.8. Error metrics and evaluation

#### Mean Absolute Error.

To evaluate the trained models, we used the mean absolute error (MAE) (single and normalized), the mean absolute percentage error (MAPE), and finally, the Squared Pearson correlation coefficient (R), which denotes the forecast’s final efficiency metric.


$$\begin{array}{c} MAE=\frac{\text{1}}{n}\sum_{i=\text{1}}^{n}\left|X_{i}-Y_{i}\right| \end{array}$$ (6)

**MAE normalized:** To eliminate the scale differences of the different data samples, we also used a normalized type of MAE and MAPE, expressed as a percentage, according to the mean value, as follows:


$$\begin{array}{c} nMAE=\text{1}\text{0}\text{0}\frac{\frac{\text{1}}{n}\sum_{i=\text{1}}^{n}\left|X_{i}-Y_{i}\right|}{\frac{\text{1}}{n}\sum_{i=\text{1}}^{n}X_{i}}=\text{1}\text{0}\text{0}\cdot \frac{\sum_{i=\text{1}}^{n}\left|X_{i}-Y_{i}\right|}{\sum_{i=\text{1}}^{n}X_{i}}=\frac{\text{1}\text{0}\text{0}\cdot mae}{mean} \end{array}$$ (7)

**Symmetric Mean Absolute Percentage Error:**


$$\begin{array}{c} sMAPE=\text{1}\text{0}\text{0}\frac{\text{1}}{n}\sum_{i=\text{1}}^{n}\frac{\left|X_{i}-Y_{i}\right|}{(|X_{i}|+|Y_{i}|)/\text{2}} \end{array}$$ (8)

**Correlation Coefficient:**


$$\begin{array}{c} Rxy=\frac{\sum_{i=\text{1}}^{n}\left(X_{i}-\overset{―}{X}\right)\left(Y_{i}-\overset{―}{Y}\right)}{\sqrt{\sum_{i=\text{1}}^{n}{\left(X_{i}-\overset{―}{X}\right)}^{\text{2}}\sum_{i=\text{1}}^{n}{\left(Y_{i}-\overset{―}{Y}\right)}^{\text{2}}}} \end{array}$$ (9)

In all equations, **X** stands for the true values of solar energy, whereas **Y** represents the predicted (expected) values, produced by each model. The last correlation equation calculates the correlation coefficient of X and Y values, which means the correlation between the actual values (X) and the predicted values (Y).

Both nMAE and sMAPE are similar most times, such as in predicting the total energy load (demand), but they differ substantially for solar energy. For example, even for the one-hour forecast of the total energy load in Spain, using a simple univariate linear regression, the nMAE is 0.585%, while the MAPE is 0.598%. By contrast, the one-hour forecast of solar energy production is very different: 7.71% and 134.74% calculated by a linear regression model respectively, as a 100% MAPE in the second case would concern too low values (e.g., 40 MW true value vs. 80 MW forecasted value is 100% deviation, but at a very small value compared to 1,432.96 of the mean value).

**Success rate:** This metric denotes the directional accuracy of the forecast, meaning how many forecasts correctly predict whether the future values will be higher or lower than the previous step, i.e., the expected difference (‘df’), regardless of how much lower or higher are expected to be. It is classified as binary; no success or success (0 and 1), where:


$$\begin{array}{c} \text{1}:ifdf_{t+\text{1}}=(X_{t+\text{1}}-Y_{t}/|X_{t+\text{1}}-Y_{t}|).(Y_{t+\text{1}}-Y_{t}/|YY_{t+\text{1}}-Y_{t}|)=\text{1},else\text{0},foreachifdf(t)>\text{0}, \end{array}$$ (10)

where X are the true values and Y are the expected values and dft + 1 is the expected difference for the next step in the future. When future actual values are the same as of the previous step, the actual difference is zero and is excluded from the calculation.

**Accuracy:** multiclass F1 score and cross-validation: Similar to the previous, but without exclusion. F1 score calculates the mean accuracy of all classes, while in k-fold cross-validation, the dataset is split into k smaller sets and calculates the average score of every smaller set. These values are from 0 (no accuracy) to 1 (absolute accuracy), but we present them as a percentage to match the result format of our research.

These scores evaluate how well the models distinguish between different production-level categories. A multiclass score, for example, calculates the accuracy of forecast among different categories, which are substantial to calculate, especially to datasets with a high variance. This should be done, because the success of prediction sometimes can be achieved and be high on the classes with higher values and not on the low values, resulting in a low or misleading average. For instance, for the first dataset (hourly values), the minimum value is observed zero and the maximum 5,792. Therefore, we have created through Python’s *scikit-learn* library six classes; from 0 to 1,000, 1,000–2,000 etc. until 5,000–6,000. The final measured accuracy is the global ratio of correctly predicted instances out of the total instances and can be determined by the following formula:


$$\begin{array}{c} Accuracy=Total_{-}Correct_{-}Predictions/Total_{-}Support \end{array}$$

where:

- Total_Correct_Predictions= the count of all samples where a forecasted class matched the true class exactly and

- Total_Support= the total number of rows/samples in the dataset testing pool.

**Feature contribution:** with this metric we will gauge, which independent variables (weather) have an effect on the target (energy) and to what extent by calculating the coefficients of every model. The coefficients’ score is classified as 0, when there is no effect, otherwise the weather features contribute to the forecast. For comparison purposes and, in order to eliminate the difference of the scales of every variable and of every dataset, we adjust the coefficients according to the standard deviation of the independent variables, according to the following equation. This way, all coefficients will scale from −1–1.


$$\begin{array}{c} y=\sum_{i=\text{1}}^{n}{coef}_{i}X_{i}=\sum_{i=\text{1}}^{n}{(coef}_{i}X_{i}{sdt}_{i})(\frac{X_{i}}{{sdt}_{i}}) \end{array}$$ (11)

For ***n*** = total number of variables and *coef*_*i*_ is the coefficient of each variable

## 3. Results and discussion

### 3.1. Summary results

The results for a test size of 20% can be summarized in the following Table 2:

**Table 2:** The summary results.

#	Forecast Horizon	Model	sMAPE (%)	MAE (MW)	nMAE (%)	Success rate (%)	R	Time (sec)
1	1h	LR (baseline) multivariate	45.38	109.10	7.71	84.81	0.99470	0.55
2	1h	TCN_univariate	22.01	59.40	4.17	90.91	0.99827	1051.69
3	1h	TCN_multivariate	20.93	58.37	4.10	91.28	0.99827	803.14
	**1h**	**TCN_improvement (%)**	**53.88%**	**46.50%**	**46.82%**	**7.63%**	**0.36%**	
4	15 min	LR (baseline) multivariate	21.04	69.41	2.00	81.80	0.99968	0.77
5	15 min	TCN_univariate	33.52	82.55	2.38	71.66	0.99965	1093.30
6	15 min	TCN_multivariate	20.34	62.05	1.79	85.79	0.99973	979.78
	**15 min**	**TCN_improvement (%)**	**3.33%**	**10.60%**	**10.50%**	**4.88%**	**0.01%**	

The above table shows the results for every one of the three models tested for each forecast horizon: one hour and fifteen minutes ahead. Additionally, the green highlighting shows how much the best model improves over the linear regression baseline.

In the one-hour scenario, the TCN architecture demonstrates clear standalone value: TCN_univariate (Model 2, using only past energy values, no weather covariates) achieves sMAPE 22.01% and MAE 59.40 MW, already outperforming the multivariate linear regression baseline (Model 1, sMAPE 45.38%, MAE 109.10 MW) by a wide margin. Adding weather covariates to TCN (Model 3) yields a further modest improvement to sMAPE 20.93%.

**Fig 10:**
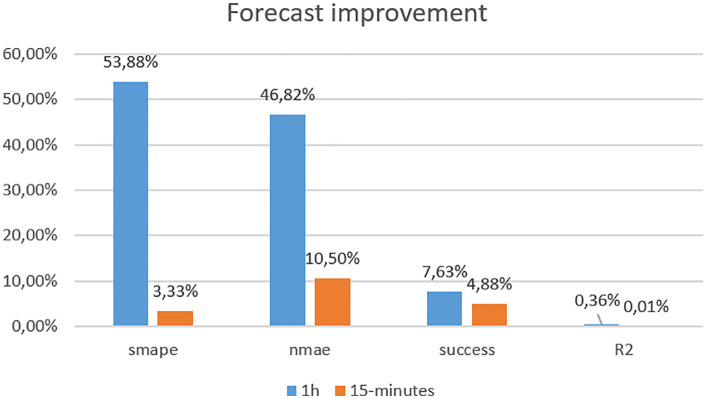
The forecast improvement using TCN.

**Fig 11:**
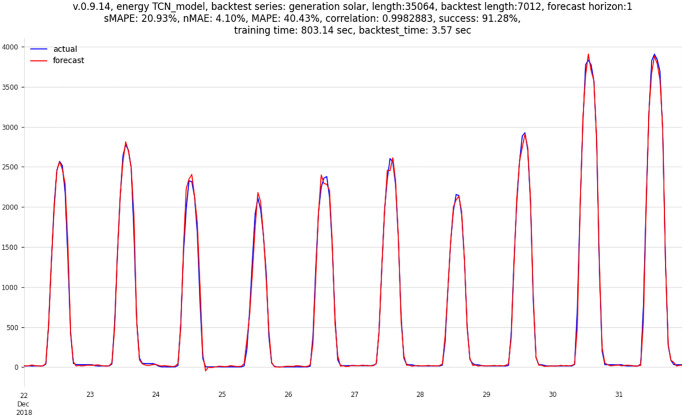
Forecast with weather data (hour ahead).

**Fig 12:**
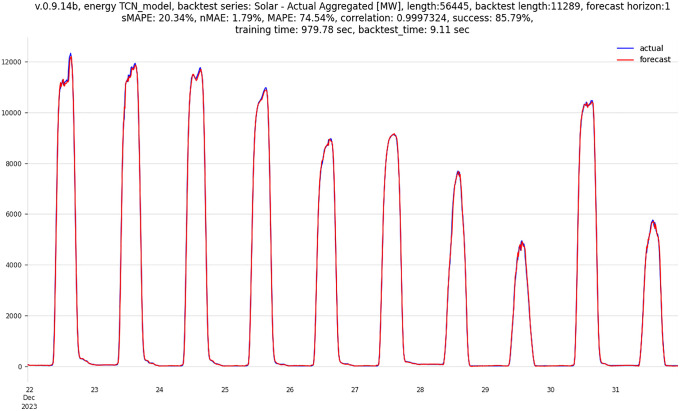
Forecast (15-minutes ahead).

The 15-minute scenario reveals a different balance between architecture and feature effects. Here, TCN_univariate (Model 5, sMAPE 33.52%, MAE 82.55 MW) performs below the multivariate linear regression baseline (Model 4, sMAPE 21.04%, MAE 69.41 MW). This comparison is not, however, a controlled test of architecture: it pits a univariate TCN against a multivariate LR, conflating the architecture effect with the feature effect simultaneously. The architecturally controlled comparison — multivariate LR (Model 4) versus multivariate TCN (Model 6, sMAPE 20.34%, MAE 62.05 MW) — shows that TCN still outperforms LR when both models receive the same weather covariates (3.3% sMAPE improvement, 10.6% MAE improvement). What the univariate result reveals is that at 15-minute resolution, weather covariates carry substantially more predictive signal than the autocorrelation structure of the energy series alone: the feature effect (TCN_univariate to TCN_multivariate: −39% sMAPE) dominates the architecture effect (LR_multivariate to TCN_multivariate: −3.3% sMAPE). This is consistent with the feature importance analysis in Section 3.4, which shows the linear regression model relying heavily on weather inputs, while the TCN model places greater weight on the lagged energy series. The overall forecast improvement is illustrated in Fig 10:

Figs 11 and 12 show the distribution of real and forecasted values of the best model (TCN_multivariate) for both forecasting horizons.

The above figures depict the last 10-day data, although the shown MAE errors concern the entire 20% of the validation data; 7,012 values for the first dataset and 11,289 values for the second respectively, which represents a very substantial test set. The maximum training iterations (epochs) of these networks were set to 100 with an early stopper patience value of 20 epochs.

### 3.2. Training and validation loss

Every neural network calculates in every iteration a loss value, which represents the mean squared error between real and forecasted data. This loss value reflects the mean squared error on data that are pre-scaled to [0, 1] using the MinMax scaler, which speeds up the training process.

**Fig 13:**
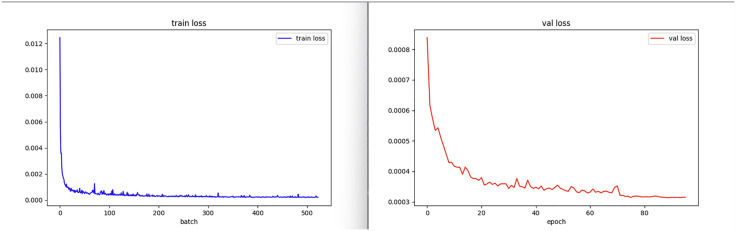
Training loss Fig. 13b Validation loss.

To assess the training performance of the neural network in our case, we can see that this is also evident from the training and validation losses, as shown for hourly-window data in Fig 13 (Fig 13a and Fig 13b):

The above figures clearly show that, both training and validation loss values decline over time as training progresses indicating good training performance, despite some natural fluctuations, mainly in the training data, until the error is minimized and approaches a horizontal line.

The minimum training loss for the 15-minute data was 1.66e-05 and the validation loss 4.47e-05. For the one-hour forecast, both values were 1.94e-04 and 3.18e-04 respectively.

Of all values in the test set, the success rate is 91.28% for the forecast horizon of one hour and 85.79% for the data with the forecast horizon of 15 minutes.

### 3.3. Accuracy: cross-validation and F1 scores

The accuracy achieved by all neural network models exceeds that of the linear regression models, both in F1 score and cross-validation, as shown in the following Table 3.

**Table 3:** Model accuracy: F1 scores and cross-validation.

#	model	f1_score (accuracy)	cross-validation (accuracy)
1	LR (baseline) multivariate	92.07%	92.07%
2	TCN_univariate	95.38%	95.38%
3	TCN_multivariate	95.65%	95.65%
	**TCN_improvement (%)**	**3.89%**	**3.89%**
4	LR (baseline) multivariate	94.06%	93.95%
5	TCN_univariate	94.55%	94.43%
6	TCN_multivariate	94.69%	94.58%
	**TCN_improvement (%)**	**0.67%**	**0.67%**

As we can see from table 3, the accuracy scores of the neural net are all above 95.50% (0.9550) for the first dataset and over 94.60% for the second one. This means that the precision is achieved through all six classes of data; both of low and high data values. In a more detail, we found out that the lower precision was observed in the data of range 3,000–4,000 MW (class #3: accuracy 87%) and the higher on the low ranges (0–1,000 MW, class #0: 99%). However, even for the last class #5 (5,000–6,000 MW, the accuracy is 97%.

### 3.4. A real-world scenario: prediction out of the sample

Using the neural network models, we were able to predict the energy production after the end of the datasets; since the first dataset ends on 31.12.2018 23:00–00:00 and the second one ends in 31.12.2023 23:45–00:00, the starting points were 01.01.2019 00:00 and 01.01.2024 00:00 respectively.

**Fig 14:**
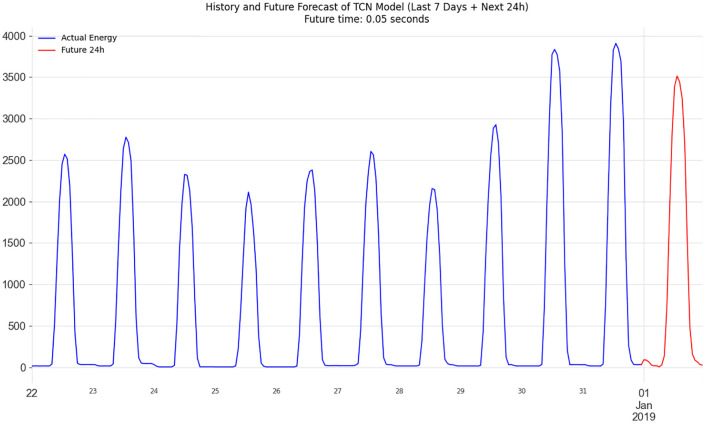
Prediction out-of-the sample (1-hour dataset).

**Fig 15:**
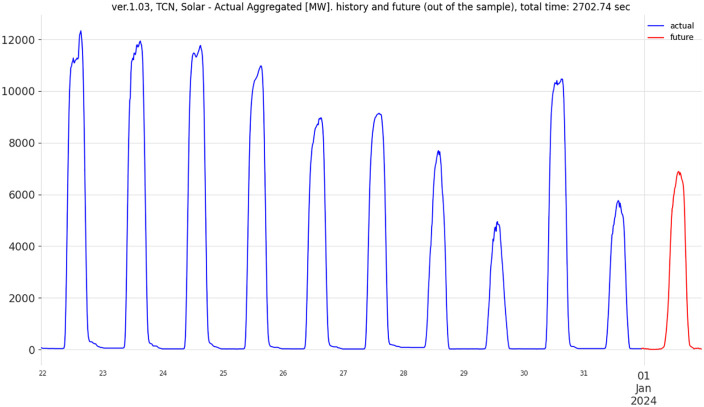
Prediction out-of-the sample (15-minute dataset).

The following figures (Fig 14 and Fig 15) show the 24-hour forecast, 24 steps ahead for the first dataset and 96 steps ahead on the 15-minute interval for the second one.

Regarding hardware resources, we measured the maximum resource requirements for this real-world scenario, including RAM usage and Video RAM used by the Graphics card [29], as well as the total time to complete each dataset. The following Table 4 shows how much RAM and Video RAM is required to complete the scenario, as well as the total training time for each dataset.

**Table 4:** Prediction out-of-the sample. Hardware resources and time.

dataset	RAM (GB)	VRAM (MB)	time (sec)	time (min)
1-hour	19.7	289.1	1,539.8	25.6
15-minute	21.3	527.3	2,702.7	45.0

RAM usage was around 20 GB, more or less, for both models, but video RAM is much less, although almost double for the larger 15-minute dataset, which is nearly twice the size of the first. At this point, we should mention that these amounts of VRAM usage are relatively low, considering that 12,228 MB of the total VRAM of the specific graphics card (only 2.36% and 4.31% for each dataset) and this is a result of the data conversion into ‘float32’ types.

On the other hand, total time ranges from around twenty and a half minutes for the first case and around forty in the second. This means that, if we were asked to predict the next hour (or the next 24 hours) within a time frame of less than an hour (e.g., to publish the early prediction of the upcoming hour or hours online), we would have succeeded. Nevertheless, by using the second dataset, we would certainly not be able to complete a 15-minute forecast with the 15-minute time window.

### 3.5. Feature importance and collinearity

**Fig 16:**
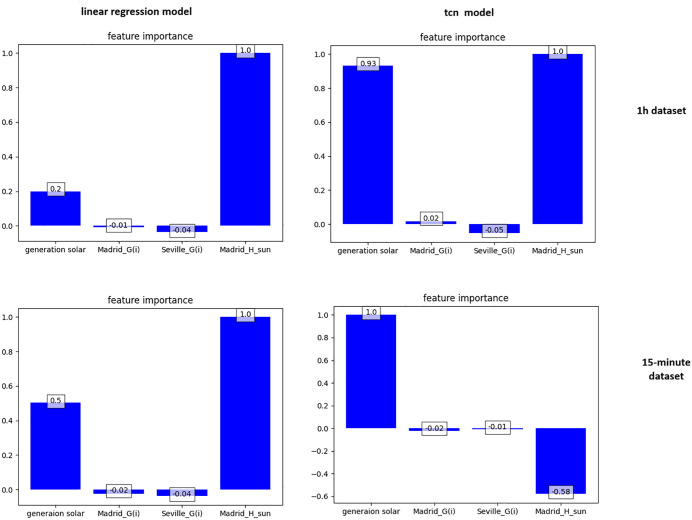
Feature importance. Regression coefficients on validation data.

The importance coefficients of regression for both multivariate models are shown in the following Fig 16

What we observe here is:

i) There is no variable without an importance, as no coefficient is zero.
ii) The past data of the target variable (solar energy production) play a more important role in the TCN models (coefficients 0.93 and 1 for the two datasets respectively), while the linear regression mostly relies on the external features, such as the height of the sun (variable ‘H-sun’ measured in degrees), with the coefficients being lower than 0.2 and 0.5 respectively.
iii) The sun’s height is an important factor for all models, although with a negative coefficient in the case of the TCN model and the 15-minute dataset.
**iv)** As shown in the above figure, g(i) variables of irradiance do not seem to play an important role. Nevertheless, we cannot exclude them, since they are statistically significant and, in this case, we could get a bigger error (nMAE). For the one-hour dataset the error would increase from 4.10% to 4.13%, and 1.88% from 1.79% for 15-minute dataset respectively. For the entire dataset, without training and validation sets, we can measure the correlation importance of each input variable to the target, as shown in the following Table 5:

**Table 5:** Regression coefficients and impact (all data).

Coefficients^a^								
Model		Unstandardized Coefficients		Standardized Coefficients	t	Sig.	Collinearity Statistics	
		B	Std. Error	Beta			Tolerance	VIF
1	(Constant)	420.423	6,431		65.374	0.000		
	Madrid_G(i)	1.259	,065	.216	19.477	.000	.076	13.181
	Seville_G(i)	2.322	,082	.406	28.378	.000	.046	21.907
	Madrid_H_sun	89.163	2,464	1.082	36.184	.000	.010	95.734
	Seville_H_sun	−69.172	2,459	−.877	28.128	.000	.010	104.226

a. Dependent Variable: generation solar

In the real-world scenario, when all data are treated both as training and validation sets, from the above table, we can see that all input variables are important with a statistical significance below 0.05 (column ‘Sig’ for a two-tailed significance) with the standardized coefficient (column ‘beta’ unnormalized) being above 1 for the variable ‘Madrid_H_sun’ and too low for the others. Nevertheless, because all variables have a statistical significance, this is why the errors would be higher for the validation data, if we exclude the less important variables.

Finally, collinearity is observed when the weather variables are related to each other as they have similar or even higher correlation to the target data, e.g., Madrid global irradiance and Madrid sun height show similar correlation to the energy of round 0.80, while there is a stronger correlation between them (around 0.94). In the above table, the VIF (Variance Inflation Factor) column confirms that several weather predictors — particularly the sun-height variables — exceed the conventional multicollinearity threshold of VIF > 10, and in the case of Seville_H_sun (VIF ≈ 104) even surpass the threshold of 100. It is important to recognise, however, that VIF is a diagnostic tool developed for ordinary least-squares (OLS) linear regression, where multicollinearity inflates the variance of coefficient estimates and undermines their interpretability. In a neural network context no such linear coefficients are estimated, so high VIF values do not compromise model stability or predictive accuracy. This is confirmed empirically by the model results: the multivariate TCN achieves R² values of 0.9983 and 0.9997 for the 1-hour and 15-minute datasets respectively, demonstrating that the correlated weather features collectively provide highly informative signals. The VIF analysis was therefore included to characterise inter-feature collinearity structure, not to apply regression-style exclusion criteria.

### 3.6. Discussion

#### 3.6.1. Energy forecast.

Forecasting for a single area or power station is less complex than forecasting for an entire country. In the latter case, both energy and weather values must be calculated as averages or totals across different regions, each with its own unique characteristics. For example, solar energy production may be higher in Central Spain, where larger generators are located, compared to the northern regions. Additionally, although summer weather is generally similar throughout Spain, specific days may be cloudy in some areas while sunny in others. Despite these challenges, this study has confirmed the feasibility of predicting solar energy production for an entire country composed of numerous power production units, as posed by the research questions in the Introduction

According to Mousa Afrasiabi et al. [30], the objective of the analysis and forecast should be to minimize energy loss and the agent’s operation cost. These include all power production means, such as conventional distributed generators, and emerging more environment-friendly technologies like wind turbines, photovoltaics, battery storage systems, etc. For this purpose, the current research’s forecast horizon has been extended from short-term up to mid-term forecast, i.e., from just one hour to a week [31] or even an entire month [32]. The latter highlights the need to address the problem of estimating the energy consumption on the customer level. At the same time, from the study described in this paper, it is observed that the problem of energy consumption forecasting is a time series regression task, as this is evident from the current literature and provided that a kind of linearity should exist (linear or logarithmic), and seasonality as well.

Deep learning algorithms, with the help of artificial neural networks, especially LSTM models, are now commonly used, as they provide higher capabilities and potentials than traditional forecasting tools, such as linear regression models. It is crucial, however, not to overestimate these potentials, especially in long-term forecasting and, more specifically, in the field of energy, as energy production or consumption is affected by various conditions and parameters. Long-term estimates of energy potential for the next 100 years, for example, cannot be reliable as it is not possible to foresee the parameters that influence it, such as climate behavior, population growth, development of the economy, etc.

Among prior studies targeting national-level solar energy production in Spain, Cabello-López et al. [20] provide the most direct point of comparison. Using ESIOS data with a multivariate CNN-LSTM architecture, they achieve a mean absolute error of 67.32 MWh for the 1-hour horizon, outperforming the official Spanish system operator forecast by 47.58%. The present work achieves 58.37 MW for the same horizon, a broadly comparable result; a direct numerical comparison is limited, however, by differences in dataset coverage periods (2015–2018 in our 1-hour dataset versus 2015–2021 in Cabello-López et al.), during which Spain’s installed solar capacity grew substantially. In relative terms, our multivariate TCN achieves nMAE values of 4.10% (1-hour) and 1.79% (15-minute). The 15-minute resolution at national level has not been addressed by prior studies, making the latter result a novel contribution without a direct published benchmark.

Bhatt et al. (2022) [33] used a total dataset of 18,277 values from average 15-minute interval global solar irradiance data collected from January 1, 2016, to January 6, 2017, from the Asian Institute of Technology (AIT) Meteorological station to train and evaluate the performance of the DL models. This work aimed to forecast the solar irradiance for various forecast horizons/steps, from 1 step (15 minutes) to 6 steps (1 hour 30 min) ahead. Although the results regard the solar irradiance and not the solar energy production itself, it is interesting to look at the results since the researchers used (among others) a neural network (an LSTM model) to predict the irradiance on which the solar energy is based and highly associated. The MAPE in that research was calculated (for the best model) at 10.19% for a 15-minute forecast, and the correlation was 0.999, similar to our model of 0.9997. For the one-hour-ahead forecast, the MAPE was found to be relatively 70.51%, and the correlation was 0.979, certainly worse than in our model. At this point, it should be noted that forecasted irradiance values do not mean that the possible solar energy forecasted values would give the same results when the first (solar irradiance) values are used as future inputs to the second (solar energy), but rather would definitely result in lower than those due to the learning and regression process of the used model, while require a 100% correlation of solar irradiance data with solar energy to be so, which may not always be the case.

**Fig 17:**
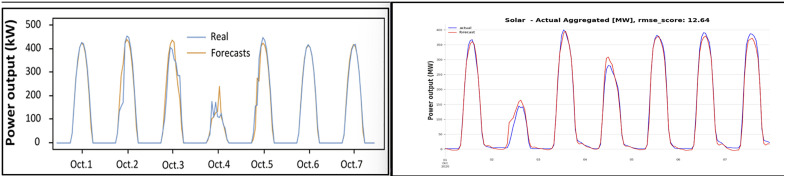
Solar energy forecast (one hour ahead). Solar energy forecast in Spain.

Y. Li et al. (2023) [17] use 5-minute field data collected from 95 utility-scale PV farms in North Carolina spanning January to November 2020, split into training (70%), validation (10%), and testing (20%), to develop and validate a hybrid TCN-based forecasting framework. The Root Mean Square Error (RMSE) between real and forecasted values was found 43.17, given that the maximum production was 400 KW in October. This work incorporates a physics-based model, meaning weather data, such as cloud data. Considering that in our work we have no weather data for this period, but only solar energy data, we tested a TCN model with scaled data from 0 to 400 MW, and the RMSE was found to be only 12.64, although the data distribution is quite different from those in the US, as shown in the following Fig 17 (on the left is the forecast provided by Y. Li et al).

Comparing our correlation of true and forecasted values of the 15-minute dataset with 56,445 values our TCN model, to the one found in the work of Aman et al. (2023) [34] with 52,560 10-minute values (from 24 November, 2021 to 24 November, 2022) with a hybrid CNN-LSTM model, we measure correlation coefficient of 0.99973 (for every sun condition) against the 0.9898, which is the best found of this study (with sunny weather conditions). It is evident that our model seems better in this comparison too.

The TCN model was also found to achieve the best performance in the study of M. Abdelsattar et al. (2025) [35] on forecasting the solar energy production. This comparative research has been conducted with 4,200 samples of historical records of solar power generation along with a total of 20 meteorological and solar positional features input features. From a total of eight models, TCN gave the best results; R of 0.7786 and RMSE of 429.4863. While these results are very good, they fall far short of our TCN model’s metrics, but we have to keep in mind that we have used far more data than just 4,200 records. All models, but especially neural networks show better performance as more data are available.

Apart from the above models, our TCN model also outperforms older one-hour ahead forecasting techniques (e.g., by using *python keras* [36] LSTM with the same epochs and at a similar running time), the forecast errors are clearly better than those of a linear regression, but worse than a darts TCN; MAE and nMAE were calculated 64.05 and 4.50% respectively (around 10% worse), while MAPE was 50.21 (around 30% worse). The correlation 0.9982 and success rate 87.81% are also lower than the TCN model. Around half of these differences can be observed for the 15-minute forecast.

The following Table 6 below provides a structured comparison of these studies and the proposed system.

**Table 6:** Comparison with prior studies on short-term solar energy forecasting.

Study	Method	Target/ Scale	Horizon	Key Error Metric	R²/ r
Cabello-López et al. [20] (2023)	CNN-LSTM (multivariate)	National solar energy, Spain (ESIOS data)	1h, 24h, 48h	MAE: 67.32 MWh	—
Y. Li et al. [17] (2023)	Hybrid TCN (physics-informed)	PV farm energy, US (95 plants)	Up to 6h (5-min resolution)	RMSE: 43.17 kW (rainy, 6h horizon)	—
Bhatt et al. [33] (2022)	LSTM	Solar irradiance, Thailand (single site)	15-min to 90-min	MAPE: 10.19% (15-min)	r = 0.999
Aman et al. [34] (2023)	CNN-LSTM	Solar energy, 10-min interval	10-min	—	R² = 0.9898
Abdelsattar et al. [35] (2025)	TCN	Solar energy (4,200 samples)	Short-term	RMSE: 429.49	R² = 0.779
**This work**	**TCN (past-covariate, multivariate)**	**National solar energy, Spain**	**1h + 15-min**	**sMAPE: 20.93%/20.34%; MAE: 58.37/62.05 MW; nMAE: 4.10%/1.79%**	**R = 0.9983/0.9997**

A further observation concerns the separation of architecture effects from feature augmentation effects. Across both forecasting horizons, the dominant driver of improvement differs. In the one-hour scenario, the TCN architecture itself provides the larger share of the gain: even without weather covariates, TCN_univariate reduces sMAPE by 51% relative to the multivariate LR baseline, and the subsequent addition of weather features yields only a marginal further reduction of approximately 5%. In the 15-minute scenario, the pattern reverses: the architecture contribution is modest (3.3% sMAPE improvement when comparing multivariate LR to multivariate TCN), while the feature contribution is dominant (weather covariates reduce TCN sMAPE by 39% relative to the univariate TCN). This asymmetry is consistent with the physics of solar forecasting: at hourly granularity, production is strongly autocorrelated and a temporal architecture can exploit this structure effectively; at 15-minute granularity, rapid cloud-cover transitions break autocorrelation more severely, making real-time weather inputs proportionally more valuable. These findings suggest that the two model components — TCN architecture and multivariate weather features — are complementary rather than redundant, and that their relative contributions are resolution-dependent.

#### 3.6.2. Limitations and benefits.

From a technical point of view, artificial networks show great potential compared to traditional methods. They allow for extensive customization and optimization and can be used to forecast various values, including energy.

Regarding modelling energy forecasting, it is observed that the optimal investment and operations of integrated local energy systems require medium to long-term prediction of energy consumption [8]. The problem, though, is that a very long-term energy prediction could be problematic as all prediction models lose accuracy as the forecast horizon grows. Furthermore, it is questionable how useful a prediction of this kind would be. For instance, if we have to predict an entire month for each hour of solar energy prediction, the error of the estimate would be enormous. In the aforementioned work of Bhatt et al. (2022), six forecast horizons were used to predict solar irradiance as steps of 15 minutes: from one step (15 minutes) to six steps ahead (90 minutes). The results showed that, in the last case of six steps, the MAE was increased almost seven times with the better model.

In contrast, it would be more feasible to accomplish monthly daily predictions as a summary or by using trend analysis or a combination of these two methods. Establishing predictions of shorter periods and then estimating more extended periods on greater scales is undoubtedly more reliable. If the goal is to preserve sustainable development with the protection of the environment, monitoring the energy demand on a shorter scale would provide certain benefits. These may include the following:

- Better forecast accuracy enables more efficient integration of solar energy into the grid.

- Improvement of network management and system balancing by allowing grid operators to anticipate fluctuations in solar power generation and better plan for grid stability.

- On the other hand, renewable energy generators also benefit from optimizing their intra-day and day-ahead electricity market trading strategies, increasing the profitability and efficiency of their operations [37].

As far as the needed technological infrastructure is concerned, as shown in the results, the training of neural networks requires a lot more time compared to traditional models. In addition, the requirements include modern equipment, such as powerful graphics cards and very powerful computer system configurations overall. The training time of neural networks is significantly longer than that of a simple linear regression, as our tests showed that the best forecast was found almost at the end of the training of almost 100 epochs. In addition, when using Python libraries for neural networks (e.g., PyTorch, Keras, etc.), some minor differences may be observed when neural models are executed on graphics cards on dissimilar hardware (CPU or GPU) or even with operating systems. As an example, when we executed both neural models in Ubuntu 22.04 and Windows 11 with the same libraries on the same machine, we measured a nMAE difference of 1% and 3% for the two tested datasets (4.09% in Ubuntu instead of 4.10% on Windows and 1.82% instead of 1.79% respectively). Although not very significant, we should point out that this is caused by the different computation of floating points and how these two operating systems treat python computing.

#### 3.6.3. Electricity optimization and efficiency.

With our research, we show how to optimize the forecast of the solar energy production with the use of modern AI techniques, in terms of a robust and early prediction in short or very short-term intervals; one hour or fifteen minutes. Higher precision enables better balancing of energy demand and production, allowing grid operators to monitor and intervene early in the production process. However, electricity estimation exceeds this aspect. Consistent monitoring and maintenance are crucial to guarantee dependable energy generation from PV panels. It has been found [38] that PV panels can develop cracks that can have a substantial effect on their overall performance and efficiency. Therefore, by using Deep Learning (DL) and Residual Network (ResNet) techniques, it is feasible that accurate cracking detection can be achieved using Electroluminescence (EL) images of solar panels. Another approach [39] has been used to track these panels failures by using image processing techniques and imaging technologies, based on neural networks. Other researchers [40] perform digital simulations to investigate the efficiency of a power control system by checking parameters of uncertainties and load disturbances of the frequencies inside the power grid.

In our study, we use weather data from the five major areas of Spain, taking into account that these areas can cover the most significant points of the horizon; from north to south and from west to east. In another approach of 2023 [41], not for solar energy, but for energy from wind turbines, the researchers propose to find out the advantages of specific areas in Egypt, which could be ideal for setting up major wind power generation projects, based on huge historical wind speed datasets obtained at 50 m height over 20–30 years. Finally, optimizing energy use for electric vehicles, would have a double goal: the energy efficiency and the reduction of the environmental load. This kind of research [42–45] can contribute to successfully address these two challenges by using neural networks and special engine controllers towards a motor efficiency. Specifically, the work of S. Patel, S. Yadav and N. Tiwari (2025) [44] targets to reduce environmental issues and decrease dependency on fossil fuels by optimizing driving with two engine controllers; a custom neural (CN) and an integrating deep learning (DL) adaptive controller. They prove that they have achieved a 17.5% improvement in energy efficiency compared to a traditional speed controller. The adaptive DL controller provides a 20% faster response time in regulating the torque output during dynamic driving conditions.

## 4. Conclusions

The multivariate Temporal Convolutional Network achieves R² of 0.9983 and 0.9997 for the 1-hour and 15-minute datasets respectively, with nMAE of 4.10% and 1.79%, and reduces sMAPE by 54% relative to the linear regression baseline in the hourly scenario. These results — obtained on national-level aggregated solar production for Spain using ENTSO-E data — confirm that very short-term photovoltaic nowcasting is feasible at the national grid scale, extending prior work that addressed either hourly horizons only or individual-site architectures.

The principal theoretical contribution is the demonstration that past-only weather covariates — solar irradiance and sun height — are sufficient for accurate forecasting without requiring future meteorological predictions as input. This eliminates a key circular dependency present in many feature-augmented forecasting pipelines. A secondary finding is a resolution-dependent asymmetry: at 1-hour granularity, temporal autocorrelation carries most predictive signal and the TCN architecture alone surpasses the multivariate linear baseline; at 15-minute granularity, rapid atmospheric variability reduces autocorrelation and makes weather inputs proportionally more valuable. This asymmetry has practical implications for sensor infrastructure and system design.

Feature selection showed that solar irradiance and sun height — with average correlations of approximately 0.79 (1-hour) and 0.55 (15-minute), substantially higher than temperature — were the only covariates that contributed meaningfully; temperature was excluded as insufficiently correlated (r ≈ 0.37 for 1-hour, r ≈ −0.05 for 15-minute).

Limitations include the restriction to Spanish national aggregates and fixed training windows (2015–2018 for the hourly dataset; 2022–2023 for the 15-minute dataset); as installed PV capacity grows, periodic retraining will be necessary. Future work could explore probabilistic TCN variants for uncertainty quantification, extend the forecasting horizon beyond 15 minutes, and investigate transfer learning across national grids.

## Abbreviations

AI: Artificial Intelligence
ANN: Artificial Neural Networks
API: Application Program Interface
BES: Battery Energy Storage
CNN: Convolutional Neural Networks
CPU: Central Processing Unit
DL: Deep Learning
ENTSO-E: European Network of Transmission System Operators for Electricity
E-SIOS: System Operator Information System (e-sios)
GRU: Gated Recurrent Units (Neural Networks)
GPU: Graphics Processing Unit
Km/h: Kilometers per hour
KWh: Kilowatt-hours
LSTM: Long short-term memory
MAE: Mean Absolute Error
MAPE: Mean Absolute Percentage Error
MW: Mega Watts
nMAE: Normalized Mean Absolute Error
OECD: Organization for Economic Co-operation and Development
PV: Photovoltaic Plant
PVGIS: Photovoltaic Geographical Information System
RNN: Recurrent Neural Networks
TCN: Temporal Convolutional Network
TSOs: European transmission System operators
TWh: Terawatt-hours
VIF: Variance Inflation Factor
