# Supplementary material for: Very short-term production prediction for photovoltaic plants using Temporal Convolutional Networks
Source: PLoS One. 2026 Jul 28;21(7):e0354512. doi: 10.1371/journal.pone.0354512 (PMC13411920; doi:10.1371/journal.pone.0354512)
Supplement: Attachment — Submitted filename: Response to Reviewers.docx [file pone.0354512.s002.docx]

Dear Editor and Reviewers,

Thank you all for the very interesting and useful comments. We have created a major revision of the manuscript according to your comments, rewriting and enriching most parts especially after the first Section of Introduction. Below you can see the highlights of this revision:

1. A flowchart, which depicts the research protocol
2. New metrics, equations and results, in a more detail, such as accuracy scores, f1_scores and cross-validation
3. A real-world scenario, where we predict the future, after the end of each dataset, as in real life (out-of-the sample)
4. We have included the feature contribution, meaning which (input) variables and how much they affect the target variable, both on the validation data and all data of the real-world scenario
5. Added a new subsection in Discussion to deal with the energy optimization and efficiency
6. The data availability is now feasible through our Github repository, on which we have uploaded the used datasets, including both energy and weather features in each one.
7. A new map was created, by using a *Carto Street map*, according to license (CCAL) CC BY 4.0
8. Added suggested literature to improve understanding of the main highlights of this research
9. Rewriting many parts to better clarify main aspects and issues, and added additional explanations on specific matters, e.g. the difference of using past and future features or how the baseline work is selected
10. The revision has been conducted to fully meet the PLOS ONE’s publication criteria.

Below, you can see our 39 responses to all comments respectively, in bold fonts.

We hope you will find interesting and suitable for publication.

Kind Regards,

The authors

Dr. Loukas Samaras

Prof. Elena García-Barriocanal

Prof. Miguel-Angel Sicilia

Lino González García

**Response to Comments:**

I. **Journal Requirements:**

When submitting your revision, we need you to address these additional requirements.

1. Please ensure that your manuscript meets PLOS ONE's style requirements, including those for file naming. The PLOS ONE style templates can be found at

<https://journals.plos.org/plosone/s/file?id=wjVg/PLOSOne_formatting_sample_main_body.pdf> and

[https://journals.plos.org/plosone/s/file?id=ba62/PLOSOne_formatting_sample_title_authors_affiliations.pdf](https://track.editorialmanager.com/CL0/https:%2F%2Fjournals.plos.org%2Fplosone%2Fs%2Ffile%3Fid=ba62%2FPLOSOne_formatting_sample_title_authors_affiliations.pdf/1/010f019c0cbebf88-09334853-f1b6-406e-b20b-f89d156a70a5-000000/pYUWjCMSbPlV0-K79-vR-K_yX5rTwP3qDTO2FGgUUXo=248)

**Response from authors (1)**: ok. The revised manuscript has been corrected according to the templates

2. Please note that PLOS One has specific guidelines on code sharing for submissions in which author-generated code underpins the findings in the manuscript. In these cases, we expect all author-generated code to be made available without restrictions upon publication of the work. Please review our guidelines at <https://journals.plos.org/plosone/s/materials-and-software-sharing#loc-sharing-code> and ensure that your code is shared in a way that follows best practice and facilitates reproducibility and reuse.

**Response from authors (2)**: ok

3. Thank you for stating the following in the Acknowledgments Section of your manuscript:

“We acknowledge the contribution of Computer Science Department the University of Alcalá de Henares and the private corporation ‘In2AI - Artificial Intelligence (AI) Consulting and Solutions’ for their support to complete this research.”

**Response from authors (3)**: ok, done.

We note that you have provided funding information that is not currently declared in your Funding Statement. However, funding information should not appear in the Acknowledgments section or other areas of your manuscript. We will only publish funding information present in the Funding Statement section of the online submission form.

Please remove any funding-related text from the manuscript and let us know how you would like to update your Funding Statement. Currently, your Funding Statement reads as follows:

“The author(s) received no specific funding for this work.”

**Response from authors (4)**: ok. **There is not funding information in the text, neither in the Acknowledgments section. There is no commercial funding.**

Additionally, because some of your funding information pertains to commercial funding we ask you to provide an updated Competing Interests statement, declaring all sources of commercial funding.

In your Competing Interests statement, please confirm that your commercial funding does not alter your adherence to PLOS ONE Editorial policies and criteria by including the following statement: "This does not alter our adherence to PLOS ONE policies on sharing data and materials.” as detailed online in our guide for authors  <http://journals.plos.org/plosone/s/competing-interests>.  If this statement is not true and your adherence to PLOS policies on sharing data and materials is altered, please explain how.

**Response from authors (5)**: ok. **The Competing Interests statement has the suggested statement included.**

Please include the updated Competing Interests Statement and Funding Statement in your cover letter. We will change the online submission form on your behalf.

**Response from authors (6)**: ok, **done**.

4. Please note that your Data Availability Statement is currently missing the repository name and or the DOI/accession number of each dataset OR a direct link to access each database. If your manuscript is accepted for publication, you will be asked to provide these details on a very short timeline. We therefore suggest that you provide this information now, though we will not hold up the peer review process if you are unable.

**Response from authors (7)**: ok, done. We have the data repository link of Github withon the manuscript in Section 2 ( <https://github.com/luxorsatellite/Darts_tcn/tree/main>)

5. Thank you for stating the following in the Competing Interests section:

“The authors have declared that no competing interests exist.”

**Response from authors (8)**: ok, done.

We note that one or more of the authors are employed by a commercial company:  In2AI - Artificial Intelligence (AI) Consulting and Solutions

1.  Please provide an amended Funding Statement declaring this commercial affiliation, as well as a statement regarding the Role of Funders in your study. If the funding organization did not play a role in the study design, data collection and analysis, decision to publish, or preparation of the manuscript and only provided financial support in the form of authors' salaries and/or research materials, please review your statements relating to the author contributions, and ensure you have specifically and accurately indicated the role(s) that these authors had in your study. You can update author roles in the Author Contributions section of the online submission form.

Please also include the following statement within your amended Funding Statement.

“The funder provided support in the form of salaries for authors [insert relevant initials], but did not have any additional role in the study design, data collection and analysis, decision to publish, or preparation of the manuscript. The specific roles of these authors are articulated in the ‘author contributions’ section.”

If your commercial affiliation did play a role in your study, please state and explain this role within your updated Funding Statement.

**Response from authors (9)**: ok, done

2. Please also provide an updated Competing Interests Statement declaring this commercial affiliation along with any other relevant declarations relating to employment, consultancy, patents, products in development, or marketed products, etc.

Within your Competing Interests Statement, please confirm that this commercial affiliation does not alter your adherence to all PLOS ONE policies on sharing data and materials by including the following statement: "This does not alter our adherence to  PLOS ONE policies on sharing data and materials.” (as detailed online in our guide for authors <http://journals.plos.org/plosone/s/competing-interests>) . If this adherence statement is not accurate and  there are restrictions on sharing of data and/or materials, please state these. Please note that we cannot proceed with consideration of your article until this information has been declared.

Please include both an updated Funding Statement and Competing Interests Statement in your cover letter. We will change the online submission form on your behalf.

**Response from authors (10): ok. Both statements have been included in the Cover Letter**

6. Please include your full ethics statement in the ‘Methods’ section of your manuscript file. In your statement, please include the full name of the IRB or ethics committee who approved or waived your study, as well as whether or not you obtained informed written or verbal consent. If consent was waived for your study, please include this information in your statement as well.

**Response from authors (11): ok. There is no ethic statement nor approval, because no personal data were used.**

7. Please ensure that you refer to Figures 3, 5, 6, 8 and 12 in your text as, if accepted, production will need this reference to link the reader to the figure.

**Response from authors (12)**: ok.

8. We note that Figure 4 in your submission contain [map/satellite] images which may be copyrighted. All PLOS content is published under the Creative Commons Attribution License (CC BY 4.0), which means that the manuscript, images, and Supporting Information files will be freely available online, and any third party is permitted to access, download, copy, distribute, and use these materials in any way, even commercially, with proper attribution. For these reasons, we cannot publish previously copyrighted maps or satellite images created using proprietary data, such as Google software (Google Maps, Street View, and Earth). For more information, see our copyright guidelines: <http://journals.plos.org/plosone/s/licenses-and-copyright>.

We require you to either (1) present written permission from the copyright holder to publish these figures specifically under the CC BY 4.0 license, or (2) remove the figures from your submission:

1. You may seek permission from the original copyright holder of Figure 4 to publish the content specifically under the CC BY 4.0 license.

We recommend that you contact the original copyright holder with the Content Permission Form (<http://journals.plos.org/plosone/s/file?id=7c09/content-permission-form.pdf>) and the following text:

“I request permission for the open-access journal PLOS ONE to publish XXX under the Creative Commons Attribution License (CCAL) CC BY 4.0 (<http://creativecommons.org/licenses/by/4.0/>). Please be aware that this license allows unrestricted use and distribution, even commercially, by third parties. Please reply and provide explicit written permission to publish XXX under a CC BY license and complete the attached form.”

Please upload the completed Content Permission Form or other proof of granted permissions as an "Other" file with your submission.

In the figure caption of the copyrighted figure, please include the following text: “Reprinted from [ref] under a CC BY license, with permission from [name of publisher], original copyright [original copyright year].”

2. If you are unable to obtain permission from the original copyright holder to publish these figures under the CC BY 4.0 license or if the copyright holder’s requirements are incompatible with the CC BY 4.0 license, please either i) remove the figure or ii) supply a replacement figure that complies with the CC BY 4.0 license. Please check copyright information on all replacement figures and update the figure caption with source information. If applicable, please specify in the figure caption text when a figure is similar but not identical to the original image and is therefore for illustrative purposes only.

The following resources for replacing copyrighted map figures may be helpful:

USGS National Map Viewer (public domain): <http://viewer.nationalmap.gov/viewer/>

The Gateway to Astronaut Photography of Earth (public domain): <http://eol.jsc.nasa.gov/sseop/clickmap/>

Maps at the CIA (public domain): <https://www.cia.gov/library/publications/the-world-factbook/index.html> and <https://www.cia.gov/library/publications/cia-maps-publications/index.html>

NASA Earth Observatory (public domain): <http://earthobservatory.nasa.gov/>

Landsat: <http://landsat.visibleearth.nasa.gov/>

USGS EROS (Earth Resources Observatory and Science (EROS) Center) (public domain): [http://eros.usgs.gov/#](http://eros.usgs.gov/)

Natural Earth (public domain): [http://www.naturalearthdata.com/](http://track.editorialmanager.com/CL0/http:%2F%2Fwww.naturalearthdata.com%2F/1/010f019c0cbebf88-09334853-f1b6-406e-b20b-f89d156a70a5-000000/KDGoiT6MeYpS_2bkTmP7itSGeS5KhC0oCxEYKsXuJyI=248)

**Response from authors (13)**: ok. **We changed the Google map with a Open Street map. This map is *licensed for reuse under CC BY 4.0*”.** and **“*Contains data sourced from the Spanish National Geographic Institute (IGN) and National Cartographic System (SCNE)***. You can see here in <https://www.openstreetmap.org/copyright>

9. If the reviewer comments include a recommendation to cite specific previously published works, please review and evaluate these publications to determine whether they are relevant and should be cited. There is no requirement to cite these works unless the editor has indicated otherwise.

**Response from authors (14)**: ok, done.

**Additional Editor Comments:**

Please prepare a substantially revised version of the manuscript addressing all substantive points raised by the reviewers. You are encouraged to thoroughly evaluate, and where appropriate defend, your original reference choices rather than adding references suggested by reviewers; include only those you consider genuinely necessary and justified in your response letter. Please also examine Reviewer 3 comments carefully and correct only those that are factually or methodologically sound, as some uploaded remarks appear to be erroneous or not applicable to your work

**Response from authors (15): ok.. We have prepared a revised manuscript with a lot of updates, mainly after the Introduction Section, in the following Sections: Methods, Results, Discussion, Conclusionsaccording to the reviewer’s recommendations.**

[Note: HTML markup is below. Please do not edit.]
Reviewers' comments:

**II. Review Comments to the Author**

Please use the space provided to explain your answers to the questions above. You may also include additional comments for the author, including concerns about dual publication, research ethics, or publication ethics. (Please upload your review as an attachment if it exceeds 20,000 characters)

Reviewer #1: 1. The writing of the paper needs a lot of improvement in terms of grammar, spelling, and presentation.

**Response from authors (16)**: ok. **Checked and corrected. Some paragraphs were rephrased to be more clear.**

2. What is the novelty of this paper?

**Response from authors (17)**: ok. As stated in the conclusions (paragraph 6):

i) the main novelty of the work reported here is not only that we use AI models with all optimizations never seen before, such as these are extensively described on the methods section with the use of the python darts library
ii) The proposed model uses solar irradiance variables as external past input data (explanatory variables) and not as future data, such as widely described in the current literature. This means that we do not first need to predict these weather features as these data are used as past variables and not as future inputs, such as widely found in the current literature. This means that given the values of the past, the future values of solar energy production are achieved straightforwardly, without the need for any initial prediction of the input variables”.

3. Add a flowchart to show what is done in this study.

**Response from authors (18)**: ok. **Done with figure 4.:**

4. Most Figures are blurred.

**Response from authors (19): ok. Figure 12 (renumber now as figure 16), which is included smaller figure 16a and 16b are now clear. If you zoom in on them and view them separately, they are now very clear. Please, see them as follows:**

5. The mathematics used throughout the article is still not very strict. Please try to update and illustrate some elements in the mathematical model that are not defined very strictly.

**Response from authors (20): ok. All the mathematic equations are strict enough, according to the mathematics’ science and the journal’s guidelines.**

6. The result part is week, results and discussion should be better explained.

**Response from authors (21): ok. The results’ section is now updated to include more information, result metrics in order to show the supremacy of neural networks over classic models. You can see accuracy scores, calculated by f1 scores, cross-validation, the importance of input features to the target variable for each model, et.c**

7. Revise the conclusions and make it concise, (Better to write the main outcomes)

**Response from authors (22): ok. In the revision, we updated the conclusions, according to the following rules:**

i) **They must be drawn appropriately based on the data presented in the result section**
ii) **They must respond to the research questions, stated in the Introduction section**
iii) **They may depict the outcome and the main contribution of this research**

8. Include some Key Suggested References:
<https://doi.org/10.1038/s41598-025-16828-2>
<https://doi.org/10.1038/s41598-025-14908-x>
<https://doi.org/10.1038/s41598-025-09101-z>
<https://doi.org/10.1038/s41598-025-98607-7>
DOI: 10.1109/MEPCON58725.2023.10462371
10.1007/978-3-031-43243-9_7
10.1109/EIConRus.2018.8317170

**Response from authors (23): ok. Thank you for suggesting these additions. We have included all, except the following two:**

i) <https://doi.org/10.1038/s41598-025-16828-2>, because we believe it is not relevant enough, as it discusses wireless optical communication systems, out of the scope of yhis research
ii) <https://doi.org/10.1038/s41598-025-98607-7> : This is also excluded, as it refers to temperature and humidity prediction and not energy itself. Furthermore, we do not use these variables in our work.

You may find them referenced in a new Sub-section (3.6.3) in Discussion Section, where we are talking about energy optimization and efficiency

Reviewer #2: The topic is relevant for grid integration of renewables; however, several methodological and reporting issues should be addressed to ensure technical rigor, fair benchmarking, and reproducibility.

1. Please clarify the exact backtesting protocol (rolling/expanding window, retraining frequency, forecast origin) and ensure strict chronological separation (no future covariate leakage via scaling/encoders).

**Response from authors (24): ok. In the revision, we have included the chart flow of this current research (Fig. 4), which actually depicts the research protocol.**

2. You claim irradiance variables are used as “past variables” so no need to forecast weather, but the now-casting setup still needs a clear statement of what is known at prediction time (e.g., at time t, are G(i) and H_sun available up to t only, and prediction is for t+1?).

**Response from authors (25): ok. In order to explain this difference, we added a new sub-section (section 2.3) mention in Materials and methods, explaining how past and input variables work, as well as the methodology difference.**

3. Linear regression alone is too weak for a time-series benchmark. Add classical TS models (e.g., persistence/naïve, SARIMA/ARIMAX, ETS), and modern ML baselines (XGBoost/LightGBM with lag features). Comprehensive Assessment of Solar Agrivoltaics Potential: Systematic Review and Techno-Economic Assessment Modeling Toward Sustainable Food and Energy Production

**Response from authors (26): ok. We thank you very much for this observation. In the revision, we now refer to what a baseline model is used for and why we preferred these over others; we can see the results in the following figures we present you in this letter. However, we kindly ask to consider the following:**

i) **first, the baseline model must be fast, and**
ii) **second, it should reveal a solid prediction of what we should expect out of the forecast proccess. Since we only use the *Python Darts* libraries, Linear Regression model fulfills these two criteria.**
iii) **if we use another model, such as SARIMAX (seasonal auto-regression moving average model with exogenous variables), we should expend more 3 hours (10,432.94 seconds) for the first dataset and more than 9 hours (34,760.35 seconds) in the back test process.**
iv) **ARIMA models, although provide very good estimation, did not result in better error metrics**
v) **Lastly, ARIMA in *Python Darts,* cannot use past variables but only future, which is not suitable in our case. The same stands for other ‘classic’ models, such as Random Forest, etc.**
vi) **Th linear regression model is also more flexible, as it can use both past and future variables.**

4. Table 2 shows TCN_univariate performing worse than LR for 15-min (higher sMAPE/MAE, lower success), while multivariate improves. Explain why the univariate TCN degrades (architecture mismatch, insufficient receptive field, scaling, seasonality settings), and include an ablation on receptive field / kernel size / dilation and lag length.

**Response from authors (27): ok. Thank you for your question.**

i) **This happens because the linear regression includes past weather data, while we have two TCN models; one without past variables and another without. This is also an insight of how can past external data can improve a model. In the summary results’ table (table 2), we now write it down to state it explicitly in the LR models.**
ii) **The Hyperparameters for the neural networks are as follows: kernel size 24, num of filters 24, batch size 24, dilation base 1 (we refer to it in the model selection). The lags are automatically use according to the input length of seasonality + forecast horizon=24+1; 24, because the seasonality is 24 hours plus the the forecast horizon of 1.**

5. Covariates are selected primarily by correlation. Please justify scientifically (and avoid target leakage) by reporting: (a) correlation computed only on training data, (b) lagged correlation/cross-correlation vs lead/lag structure, and (c) multicollinearity handling (many city variables likely correlated).

**Response from authors (28): ok. Please, see below:**

a) **We include, in the revision, the correlation coefficients computed only on training data on table 1. Both categories are the highest possible for the selected variables**
b) **We did not include lagged correlations, as the lags are automatically computed according to the data seasonality. Furthermore, the learning process of the mural networks is made in a such a way, in order to ‘learn and remember’ the best lag correlations and ‘forget’ the worse.**
c) **Multicollinearity:**

As shown in the correlation matrix heatmap (figure 7), all weather data are highly correlated to each other and to the target as well. E.g. the G(i) data (Global irradiance on the inclined plane (plane of the array) in W/m2) of Madrid is correlated 0.88, 0.93, 0.95 to Barcelona, Bilbao, Seville and Valencia respectively, because they refer to the same weather conditions (solar irradiance) in Spain. They are of course similar, although not exactly the same.

For the models, based on neural networks, to succeed, it is essential that weather data, if is to be inserted as input variables, should definitely have the larger possible correlation to the target variable, meaning that they are also expected to have the highest possible correlation among each other. Under this aspect, in neural networks no issues are occurred due to a multicollinearity, as you may see in the results.

We kindly remind that, multicollinearity has not a negative effect in a regression process, provided of course that the target (and input vars) foll the normal distribution. You can see below the distribution histogram of the dependent variable:

6. The manuscript discusses MAPE instability for low solar outputs, but still uses MAPE in discussion; also please verify the sMAPE equation formatting/denominator and report confidence intervals (bootstrapped) for MAE/nMAE/sMAPE.

**Response from authors (29): ok. Instability mainly refers to the volatility of the data, and not so much to the mape error metric. Furthermore, as it is clearly stated in the Introduction, this evaluation is not done for a stand-alone prediction but for entire country, consisting of different weather conditions and solar energy volume.**

7. Provide full hyperparameters (input length, output length, batch size, epochs actually used after early stopping, optimizer settings, weight decay, random seeds) and code for Darts pipeline. Energy-exergy and environ-economic (4E) analysis of heat storage-based single-slope solar stills integrated with solar air heater
8. “Data can be found free by the appropriate websites…” is not sufficient.

**Response from authors (30): ok, done. In the revision, we have included the hyperparameters of the neural network**

Reviewer #3: 1. The manuscript reports strong aggregate performance metrics; however, class-wise precision, recall, and F1-score are not sufficiently analyzed. Given the multi-class nature of the problem, per-class evaluation and error distribution analysis are required to assess model reliability.

**Response from authors (31): ok. We computed a multi-class f1_score values, as well a multi-sample cross-validation and we present them in a new table 3 of the results.**

2. The dataset preparation process is described, but statistical details regarding class balance before and after preprocessing are missing. A quantitative summary of class distribution and its impact on training should be included.

**Response from authors (32): ok. The impact of the input variables can be seen as follows:**

i) **In Section 3.3 of the results, with the F1_scores, calculating as the average score of all classes for each model, between the actual and forecasted values (*fig. 15*). We believe that this is enough to determine the accuracy of each model (as well as with the cross-correlation).**
ii) **In section 3.5 of the results with the feature importance of each variable**
iii) **In the last added *table 5* of the Results’ section, you can see (as preprocessing) the cross-correlation coefficients of all input variables and their statistical significance to the target variable, for the entire dataset, meaning without training and validation data**

| **Coefficients^a^** | |  |  |  |  |  |  |  |
| --- | --- | --- | --- | --- | --- | --- | --- | --- |
| Model |  | Unstandardized Coefficients | | Standardized Coefficients | t | Sig. | Collinearity Statistics | |
|  |  | B | Std. Error | Beta |  |  | Tolerance | VIF |
| 1 | (Constant) | 420,423 | 6,431 |  | 65,374 | 0,000 |  |  |
|  | Madrid_G(i) | 1,259 | ,065 | ,216 | 19,477 | ,000 | ,076 | 13,181 |
|  | Seville_G(i) | 2,322 | ,082 | ,406 | 28,378 | ,000 | ,046 | 21,907 |
|  | Madrid_H_sun | 89,163 | 2,464 | 1,082 | 36,184 | ,000 | ,010 | 95,734 |
|  | Seville_H_sun | -69,172 | 2,459 | -,877 | -28,128 | ,000 | ,010 | 104,226 |
| a. Dependent Variable: generation solar | | |  |  |  |  |  |  |

3. The model evaluation relies on a single train–validation–test split. The absence of cross-validation or repeated experiments limits confidence in generalization and robustness of the reported results.

**Response from authors (33): ok. Cross-validation has been performed and the results are now included in table 3/**

4. Comparisons with existing approaches are based on reported results from the literature rather than evaluation under identical experimental conditions. This limits the strength of comparative performance claims.

**Response from authors (34): ok. In the Discussion, we compared our findings to the results of other four relatively recent research works, which are as close as possible to our work, regarding relevance. As stated in the introduction, almost every research we have examined refers to models based on data on specific geographic locations and not to an entire country of this magnitude as Spain**

5. The proposed architecture has a high parameter count, yet no analysis of inference time, computational complexity, or memory footprint is provided, which is critical for assessing real-world applicability.

**Response from authors (35): ok. In the revision, we include a real-world scenario of predicting out-of-the sample the next 24 hours. We, therefore, present how this was done and what hardware resources were needed (given the used hardware infrastructure), with the required time as well. The results are now shown in the last table 4.**

6. While data augmentation techniques are applied, their individual contribution to performance improvement is not analyzed. An ablation study would strengthen the methodological justification.

**Response from authors (36): ok. In the revision, the contribution of features (target and input variables) s now presented in a specific results’ sub-section (3.5 Feature importance)**

7. Reproducibility is insufficiently addressed. The manuscript does not clearly state whether the code, trained models, and configuration details are publicly available, which is essential for PLOS ONE compliance.

**Response from authors (37): ok. The data are available in public and free and we mention the web pages to get it from. Furthermore, we have uploaded on a github repository, and added the URL in the start of Section 2, as follows:** [**https://github.com/luxorsatellite/Darts_tcn/tree/main**](https://github.com/luxorsatellite/Darts_tcn/tree/main)

8. The discussion section focuses mainly on performance gains and lacks analysis of failure cases or limitations, such as visually similar classes or adverse imaging conditions, which are important for a balanced scientific assessment.

**Response from authors (38): ok. This would be done, only if we had to evaluate separate data from one specific geographic location and not an entire same country, e.g. Spain. This is one of the major problems and difficulties when evaluating combined areas, as this is mentioned in the research.**

**If we could, however, have both energy data and weather data, each the Spanish region, this could be done and would certainly be an added value assessment, by classifying the entire dataset into regional datasets. We have only weather data for every region, while energy data is for all over Spain.**

9. The literature review can be strengthened by including additional references related to deep learning and neural network–based approaches.
doi.org/10.1504/IJEHV.2023.132034
<https://doi.org/10.1080/01430750.2024.2315485>
<https://doi.org/10.4271/12-08-04-0036>
<https://doi.org/10.1504/IJICA.2025.148630>

**Response from authors (39): ok, done, with following reference numbers, respectively: [88,89,90,91]**
