# Supplementary material for: Very short-term production prediction for photovoltaic plants using Temporal Convolutional Networks
Source: PLoS One. 2026 Jul 28;21(7):e0354512. doi: 10.1371/journal.pone.0354512 (PMC13411920; doi:10.1371/journal.pone.0354512)
Supplement: Attachment — Submitted filename: Response_to_Reviewers_auresp_2.docx [file pone.0354512.s003.docx]

Dear Editor and Reviewers,

Thank you all for your comments. We have created a second revision of the manuscript, by modifying and rewriting to achieve the best outcome. Below you can see the highlights of this revision:

1. Reduced over 900 words of Introduction, for a better and brief transition to the main analysis
2. More compact conclusions within 300 words
3. Further brief analysis of collinearity of weather features
4. Enhancing of some figures, so the axes can be seen better
5. Added some literature from PlosOne and limited references to only those only referring to the specific subject
6. Reduced total references to almost half of the initial count

Below, you can see our 23 responses to the comments respectively.

We hope you will find interesting and suitable for publication.

Kind Regards,

The authors

Dr. Loukas Samaras

Prof. Elena García-Barriocanal

Prof. Miguel-Angel Sicilia

Lino González García

**Response to Comments:**

Journal Requirements:

1. If the reviewer comments include a recommendation to cite specific previously published works, please review and evaluate these publications to determine whether they are relevant and should be cited. There is no requirement to cite these works unless the editor has indicated otherwise. 

Additional Editor Comments:

1. Rewrite Abstract in a single para and remove heading Background:, Objective:, Methods: and Results

**Response from Authors (1):** done as requested.

2. Replace Figure 1, Figure 2 with large size Axis fonts

**Response from Authors (2):** done for Figure 2. Figure 1 is only an image and its axes cannot be changed. Nevertheless, we have enlarged it so that it can be viewed better.

3. Fig. 4 Flowchart is not clearly visible so replace it , and text should be in black colour.

**Response from Authors (3):** done as requested with larger fonts

4. Replace Fig. 8, Fig. 12a, Fig. 12b, and Fig. 14 with large axis font

**Response from Authors (4):** done as requested with larger fonts..

5. Rewrite section 4 conclusion in 2-3 para, 200-300 words avoid numbering

**Response from Authors (5):** Removed over 600 words and now it is limited into 300 words.

6. Refer Already published research papers in Plos one for better presentation of this work.

**Response from Authors (6):**

We added the following references:

[16] Balraj G, Victoire AA, S. J, Victoire A (2022) Variational mode decomposition combined fuzzy—Twin support vector machine model with deep learning for solar photovoltaic power forecasting. PLoS ONE 17(9): e0273632. https://doi.org/10.1371/journal.pone.0273632

]17] Zameer A, Jaffar F, Shahid F, Muneeb M, Khan R, Nasir R (2023) Short-term solar energy forecasting: Integrated computational intelligence of LSTMs and GRU. PLoS ONE 18(10): e0285410. https://doi.org/10.1371/journal.pone.0285410

[18] Tomás Cabello-López, Manuel Carranza-García, José C. Riquelme, Jorge García-Gutiérrez (2023), Forecasting solar energy production in Spain: A comparison of univariate and multivariate models at the national level, Applied Energy, Volume 350,2023,121645, ISSN 0306-2619, https://doi.org/10.1016/j.apenergy.2023.121645.

[Note: HTML markup is below. Please do not edit.]

Reviewer's Responses to Questions

**Comments to the Author**

6. Review Comments to the Author

Please use the space provided to explain your answers to the questions above. You may also include additional comments for the author, including concerns about dual publication, research ethics, or publication ethics. (Please upload your review as an attachment if it exceeds 20,000 characters)

Reviewer #4: 1. Title & Abstract
• The title is clear and representative of the study.
• The abstract states the problem, data source, methods, and some numerical results.
• However, the abstract uses vague or potentially misleading wording such as “regression score” without defining whether this refers to correlation or coefficient of determination.
• The abstract would be stronger if it emphasized the practical significance of the forecast for grid balancing and clarified the exact metric names.

**Response from Authors (7):**

Thank you for your comment. The Abstract gives emphasis by stating that the *“… deep learning methods may achieve better results than traditional models in predicting and facilitating an efficient integration of solar energy into the power grid”.* This efficient integration into the power grid is the main practical significance, consisting of many detailed factors, which cannot be referred in the Abstract due to word limitation. We mention however in Discussion and Conclusions.

2. Introduction
• The problem is clearly important, and the manuscript explains why short-term solar forecasting matters for energy balancing.
• The introduction is broad and contains useful context, but it is too long and sometimes drifts into general energy background not essential to the research question.

**Response from Authors (8):** You are absolutely right. We have reduced it in over 900 words.

• The research questions are explicitly stated, which is a strength.
• The motivation is relevant, but the research gap should be framed more sharply against prior solar forecasting studies rather than against very broad energy-forecasting literature.

**Response from Authors (9):** Thank you very much. We have revised the manuscript with only most relevant literature.

3. Related Work
• The paper cites a substantial body of prior work, including solar forecasting, wind forecasting, and deep learning approaches.
• However, the comparison is often descriptive rather than critical.

**Response from Authors (10):** This is an important remark. Please, take it account that our discussion is both descriptive (whenever possible, with error metrics) and critical as well. For example, the use of weather features as future known variables, while they are not really known, the fact that some research focuses more on the solar irradiance prediction (e.g. Bhatt et al. (2022), and some researchers used much smaller datasets, e.g. Y. Li et al. (2023) use data from May, 2020 to September, etc.

• The literature review mixes directly relevant studies with less relevant material from NLP, speech, and generic convolutional-network applications, which weakens focus.

Repsonse from Authors (11):

We have removed from the text any references to studies that weakens focus and and have restructured the text accordingly

• The manuscript should compare more directly against recent solar forecasting studies using comparable targets, horizons, and datasets.

**Response from Authors (11):** Thank you very much. Indeed, we refer to the problem of forecast horizons, where the errors tend to be bigger, as bigger these forecast horizons are. For instance, in Discussion, we refer to the findings of Bhatt et al. (2022), when they used six forecast horizons to predict solar irradiance as steps of 15 minutes, *“from* ***one step*** *(15 minutes) to* ***six steps*** *ahead (90 minutes). The results showed that, in the last case of six steps, the MAE was increased almost seven times with the better model”.* Furthermore, as you can see the discussion, we only compare with short forecast horizons.

4. Methodology
• This is the most serious weakness of the paper.
• The section labeled as TCN includes LSTM-style equations for input, forget, cell, and output gates. These are not TCN equations and should not be used to describe a temporal convolutional network.

**Response from Authors (12):** Thank you very much, you are right. We have included those equations because neural networks share a lot of common functions, such as:

- The layers’ functions
- The hidden size. Although this TCN has not a specific hyper-parameter for hidden size, this does not mean that it does not incorporate hidden layers functions.

• The manuscript later mentions dilated convolutions, kernel size, and dilation base, but the overall architectural description remains conceptually inconsistent.

**Response from Authors (13):** Thank you, but we believe that the architecture is consistent enough, as it describes layer functions, dilation functions and the hyperparameters as well, as the last have been determined by tuning with optimization techniques, as these described in the text (e.g. the use of “optuna” … etc. Please, take into account that the scope of this paper is not the neural architecture analysis in a more detailed-level.

• The reported hyperparameters are useful, but reproducibility is still incomplete because the forecasting protocol, retraining schedule, scaler fitting procedure, and exact backtesting design are not fully specified.

**Response from Authors (14):** Thank you very much. You are absolutely right that the design should be clear: Please note the following:

- Training: It is carried out by specific models in the 80% of the datasets
- Evaluating- Back testing: It is done for the 20% of the data, by using forecast horizon =1 (since we want to estimate 1-hour prediction in the first dataset and 15-minutes in the second one)
- Forecasting out-of-the sample: It is done using all available data for a forecast horizon of 24 hours and 96 15-minute intervals, both to achieve a 24h hour prediction, as you can clearly see in the figures 13 and 14.
- We used scaling, although this is not necessary, but it can speed up the training. The scaling is performed through min max scaler and is mentioned now in the second revision. The used scaler is *“Scaler(scaler=MinMaxScaler()”*

• The “real-world scenario” is especially problematic because it states that all data are used simultaneously as training and validation, which is not a valid evaluation setup.

**Response from Authors (15):** In darts library of python, there are two ways of predicting out-of-the-sample:

a) By training 80% of the data and evaluating the best model of the 20% validation size or
b) By training all data before the predictions (out of-the-sample).

We choose the second option, as the errors will be lower, due to the higher data volume. Below, you can see the comparison of training the 80% of the data with the training of the 100% of data (scaled loss= Mean Standard Error, torch.nn.MSELoss() with forecast horizon 24 and ouput_chunk_length=24):

100 %

80 %

As you can see in the above graphs, they seem similar, but the second training of all data shows a minimum loss of 0.00065760436700657, while training of 80% data has much bigger one, 0.0013787125935778, over 2.09 times bigger.

• Covariate selection is based mainly on correlation, while strong multicollinearity is acknowledged but not handled rigorously. The response that the network can learn the best lag structure is not a sufficient methodological justification.

**Response from Authors (16):** Thank you very much for your comment; you are right that collinearity can be sometimes a problem. Please, note that following:

- The feature selection is based, most times, on feature contribution and correlation, which are both analyzed in our work,
- The results clearly backed up the selected features,
- Weather data from different locations of a same (European) country, are not quite similar, but not quite different,
- As we stated in our previous review comments, it has to do weather their distribution is normal or not; in our case, both the target and the weather fatures follow the normal distribution,
- At the end of the new revised results, we analyze in brief the VIF (Variance Inflation Factor) and tolerance, although these are mostly related to regression, rather than neural networks, which have a different way of functioning (remember and forget), as clearly shown in feature contribution results.

5. Results
• Results are presented numerically in tables and visually in plots, which is good.
• The hourly multivariate TCN improves clearly over the reported linear-regression baseline, especially in sMAPE and nMAE.
• However, the 15-minute univariate TCN performs worse than the linear-regression baseline, while the multivariate TCN gives only modest gains. This deserves a fuller explanation.

**Response from Authors (15):**

| **#** | **forecast horizon** | **model** | **smape** | **mae** | **nmae** | **success** | **R^2^** | **time (sec)** |
| --- | --- | --- | --- | --- | --- | --- | --- | --- |
| 1 | 1h | LR (baseline) **multivariate** | 45.38 | 109.10 | 7.71 | 84.81 | 0.99470 | 0.55 |
| 2 | 1h | TCN_univariate | 22.01 | 59.40 | 4.17 | 90.91 | 0.99827 | 1051.69 |
| 3 | 1h | TCN_multivariate | 20.93 | 58.37 | 4.10 | 91.28 | 0.99827 | 803.14 |
|  | **1h** | **TCN_improvement (%)** | **53.88%** | **46.50%** | **46.82%** | **7.63%** | **0.36%** |  |
| 4 | 15 min | LR (baseline) **multivariate** | 21.04 | 69.41 | 2.00 | 81.80 | 0.99968 | 0.77 |
| 5 | 15 min | TCN_univariate | 33.52 | 82.55 | 2.38 | 71.66 | 0.99965 | 1093.30 |
| 6 | 15 min | TCN_multivariate | 20.34 | 62.05 | 1.79 | 85.79 | 0.99973 | 979.78 |
|  | **15 min** | **TCN_improvement (%)** | **3.33%** | **10.60%** | **10.50%** | **4.88%** | **0.01%** |  |

It is true. However, this happens when comparting the **univariate** TCN model to a **multi-variate** Linear Regression. When we used weather features the performance gain is obvious, although not so much big, as in the first dataset.

• The benchmark is too weak: comparison only against linear regression is not enough to support strong claims about superiority of AI or deep learning.

**Response from Authors (17):**

We sincerely appreciate the reviewer’s comment. We would like to clarify that our intention is not to provide an exhaustive comparison of all available models, but rather to establish a benchmark model that is both computationally efficient and sufficiently accurate for the purposes of our study, as described in the “Model Selection” section. We have added the necessary clarifications regarding this point in the text

• Metrics are also not fully satisfactory. The manuscript uses MAE, nMAE, MAPE/sMAPE, “success,” F1 score, and cross-validation “accuracy,” but the classification-style metrics are insufficiently justified for a regression problem.

**Response from Authors (18):**

Thank you for this valuable comment. We would like to clarify that the problem addressed in this work is not formulated as a regression task. The inclusion of metrics such as MAE, nMAE, and MAPE/sMAPE is intended to facilitate comparison and to provide complementary perspectives on model performance.

In addition, classification-oriented metrics are employed to evaluate the predictive performance in terms of categorical outcomes. Specifically, we use the multiclass F1-score within a k-fold cross-validation framework, as it provides a robust assessment in settings where class imbalance may be present and where accuracy alone may be insufficient

• The manuscript also refers to Pearson correlation as R2, which is conceptually confusing.

**Response from Authors (19):**

The R^2 coefficient represents the square of the Pearson correlation coefficient (r). It represents the proportion of variance in the dependent variable explained by the model, typically ranging from 0 to 1. Pearson correlation is distinguished from other correlation coefficients, such as the Spearman's and Kendall’s coefficients. We added for the sake of clearness the word “Squared” in the description to make this clearer.

6. Discussion
• The discussion identifies some practical implications of improved short-term forecasting.
• However, several claims are overstated. The manuscript argues that its results are better than most previous studies, but many of those comparisons are not like-for-like because they involve different targets, geographies, horizons, and covariates.

**Response from Authors (20):**

Thank you for this important observation. We agree that direct comparisons with prior studies must be interpreted with caution, as differences in targets, geographical settings, forecasting horizons, and input variables limit strict like-for-like evaluation.

We have revised the manuscript to moderate the strength of our claims and to more clearly acknowledge these limitations, emphasizing that our results should be interpreted within the specific context of our study rather than as universally superior to prior work.

• Some parts of the discussion move too far from the core contribution, especially the subsection on “electricity optimization and efficiency,” which reads more like a general expansion than a focused interpretation of the present results.

**Response from Authors (21):**

In the conclusions, we clearly state that *“Prediction models can be used to manage the balance between energy demand and production. They can help effectively reduce power consumption, specifically for short or mid-term forecasts”.* This balance, however cannot be achieved without the optimization of electricity demand and forecast. This is the core effort of all scientists, either on the production field or in the application area (e.g. electrical motors).

• Limitations are mentioned, but they should be discussed more directly: dependence on selected weather variables, potential leakage issues, limited baseline set, and uncertain generalizability beyond Spain.

**Response from Authors (22):**

We appreciate the concern regarding the focus of the discussion section. Our intention in including the subsection on electricity optimization and efficiency was to contextualize the practical relevance of accurate forecasting. As stated in the conclusions, prediction models can support the balance between energy demand and production, particularly in short- and mid-term horizons. Achieving this balance is inherently linked to the optimization of both electricity demand and forecasting processes.

That said, we have written this subsection to more clearly connect it to the core contribution of the manuscript and to avoid overly general statements, ensuring that the discussion remains tightly aligned with the findings of our study.

7. Conclusion
• The conclusion restates the main findings and links them back to the research questions.
• However, it repeats some over-strong claims and should be moderated.
• A stronger conclusion would summarize the real contribution more carefully: country-level PV now-casting with public data, evidence that past weather covariates help, and a need for stronger future validation and benchmarking.
8. References
• The paper includes both older foundational references and some recent works.
• That said, the reference list is unevenly curated. Some citations are only indirectly relevant, and the literature review should be tightened around recent solar-energy forecasting studies.
• Formatting and consistency also need attention.

Originality
The application of TCN-style forecasting to aggregated national PV generation is potentially valuable, especially with both hourly and 15-minute public data. The idea is not wholly novel at the algorithmic level, but the application context has some originality.
Contribution
The paper’s main contribution is practical rather than theoretical: it explores whether short-term country-scale PV now-casting is feasible and whether weather covariates improve it. That contribution is useful, but it is currently weakened by poor benchmarking and unclear methodology.
Technical quality
Currently not strong enough. The incorrect TCN description, weak validation design, unclear regression/classification metrics, and limited baselines all reduce confidence in the technical rigor.
Clarity
The manuscript is difficult to read in places because of grammar issues, inconsistent terminology, and formatting artifacts.
Organization
The paper has a recognizable structure, but numbering, section flow, and presentation need cleanup. Discussion material is not always well separated from results.
Main weaknesses
• Incorrect TCN methodological description.
• Weak baseline comparison.
• Unclear temporal validation and possible leakage concerns.
• Poorly justified use of classification-style accuracy metrics for regression.
• Overstated comparative claims.
• Serious formatting and manuscript-hygiene problems

**Response from Authors (23): Conclusions, References and Originality:**

a) Contribution, technical quality, clarity and organization: We have performed a revision in a best way, so that we can clearly state what we are doing and why, in reference to previous studies
b) Conclusions: The conclusions in this revision have been written in a shorter way, to avoid possible confusions.
c) References: The references are now limited to around half (46) targeting to the specific subject. Formatting and consistency have also been reviewed.
d) Originality: We agree that the methodological foundations are not entirely novel at the algorithmic level. However, as clarified in both the cover letter and the manuscript, the main contribution of this work does not lie in the forecasting model per se, but in the way past covariates are systematically incorporated as inputs within a unified framework.

In particular, our approach leverages the Darts library, extending its application through a comprehensive optimization strategy that includes learning rate tuning, hyperparameter optimization, and the use of automated datetime encoders, among other elements. To the best of our knowledge, this combination and its application to aggregated national PV generation data have not been explored in prior work.
