# Supplementary material for: Very short-term production prediction for photovoltaic plants using Temporal Convolutional Networks
Source: PLoS One. 2026 Jul 28;21(7):e0354512. doi: 10.1371/journal.pone.0354512 (PMC13411920; doi:10.1371/journal.pone.0354512)
Supplement: Attachment — Submitted filename: Response_to_Reviewers_R3.docx [file pone.0354512.s004.docx]

**Response to Reviewers — Third Revision (R3)**

**Manuscript:** Very short-term production prediction (now-casting) for photovoltaic plants using Arti- ficial Intelligence and Temporal Convolution Networks **Manuscript ID:** PONE-D-25-68879R2 **Jour- nal:** PLOS ONE **Deadline:** July 11, 2026

Dear Editor and Reviewers,

Thank you for the continued and careful review of our manuscript. Below we address each comment raised in this third round of review. Changes in the revised manuscript are highlighted in the track- changes version.

Kind Regards, The Authors

Dr. Loukas Samaras Prof. Elena García-Barriocanal Prof. Miguel-Angel Sicilia Lino González Gar- cía

# Editor Comment

\#	Editor Comment	How Addressed

E1	At this stage fulfill and answer all the reviewers’ comments carefully.

All comments from Reviewer #5 and Reviewer #6 have been addressed individually below, with corresponding changes made to the manuscript. In addition, we have carried out a careful revision of the English language and terminology consistency throughout the manuscript, correcting typographical errors, standardizing technical terms (e.g., “Temporal Convolutional Networks”, “photovoltaic plants”), and ensuring that metric names and notation are used consistently across all sections.

# Reviewer #5

R5.1	**TCN equations (Section 2.5):** Equations (3)–(8) define recurrent gating mechanisms (i_t, f_t, o_t, c_t, h_t) characteristic of LSTM, not TCN. TCN architectures rely on causal convolutions, dilated convolutions, receptive fields, and residual connections. The current formulation creates substantial methodological ambiguity. The manuscript should include equations specifically related to TCN operations and clarify whether Equations (3)–(8) are included for conceptual background only, or whether recurrent components were actually incorporated.

R5.2	**sMAPE formula (Equation 12):** The denominator is written as [(X_i - Y_i)/2] whereas the conventional sMAPE definition uses [(|X_i| +

|Y_i|)/2]. As written, numerator and denominator are derived from the same difference, causing the expression to simplify to nearly constant values (≈

±2), which does not function as a normalized symmetric percentage error. The authors should verify whether this is a typographical error only or whether the metric was also implemented incorrectly, as this could affect the validity of reported results.

The reviewer is correct. The LSTM gating equations (former Eqs. 3–8) have been removed from Section

2.5 and replaced with three TCN-specific equations: Eq. (3)

causal convolution, Eq. (4) dilated causal convolution (including the receptive field formula 1 + 2·(k−1)·(2^L − 1)), and Eq. (5) residual connection. All downstream equation numbers have been updated accordingly (former Eqs. 10–15 are now Eqs. 6–11), and the in-text reference to “equation 9” in Section 2.6 has been updated to “equation 4”.

The reviewer is correct. The implementation used the correct denominator (|X_i| + |Y_i|)/2 throughout — confirmed by the reported sMAPE values (21.04%, 33.52%, 20.34%), which cannot arise from the erroneous formula (which reduces algebraically to ≈

±200%). The error was typographical only. Eq. 8 has been corrected accordingly. No results are affected.

R5.3	**Performance attribution (Table 2):** The TCN_univariate model performs worse than the LR baseline in the 15-minute scenario (sMAPE: 33.52 vs 21.04; MAE: 82.55 vs 69.41). This

suggests that the performance gains are primarily driven by the inclusion of external weather covariates, not by the TCN architecture itself. A clearer separation between (i) the effect of the neural network architecture and (ii) the effect of multivariate feature augmentation is needed to avoid attributing improvements solely to TCN.

The reviewer is correct. The Summary Results section has been rewritten to separate architecture and feature effects. The architecturally controlled comparison (same features, LR vs TCN) shows TCN outperforms LR at both resolutions: 54% sMAPE reduction at 1 hour (Models 1 vs 3:

45.38% → 20.93%) and 3.3%

sMAPE / 10.6% MAE improvement at 15 minutes (Models 4 vs 6:

21.04% → 20.34%; 69.41 → 62.05

MW). The feature-effect comparison (same architecture, TCN univariate vs multivariate) shows weather covariates reduce sMAPE by 39% at 15 minutes

(Model 5 vs 6). The

resolution-dependent asymmetry — architecture-dominant at 1 hour, feature-dominant at 15 minutes — is discussed in a dedicated paragraph added to Section 3.6.

| R5.4 | **VIF interpretation:** Several predictors show very | The reviewer is correct. The |
| --- | --- | --- |
|  | high VIF values (e.g., Madrid_H_sun VIF = | erroneous comparison of VIF |
|  | 95.734; Seville_H_sun VIF = 104.226), which | values to the dependent variable |
|  | standard practice considers indicative of severe | mean (1,432.82 MW) has been |
|  | multicollinearity. The manuscript interprets these | removed. The revised text explicitly |
|  | as “relatively low” by comparing them to the | acknowledges that all weather |
|  | magnitude of the dependent variable, which is not | predictors exceed VIF > 10 — |
|  | consistent with standard VIF interpretation (VIF is | particularly the sun-height variables |
|  | dimensionless and not defined relative to the | (Madrid_H_sun = 95.7, |
|  | scale of the dependent variable). Clarification is | Seville_H_sun = 104.2) — and that |
|  | needed on whether this is intentional or whether | Seville_H_sun also exceeds the |
|  | conventional thresholds (e.g., 5 or 10) should be | threshold of 100. We clarify that |
|  | applied. | VIF is an OLS diagnostic: in a |
|  |  | neural network no linear |
|  |  | coefficients are estimated, so high |
|  |  | inter-predictor correlations do not |
|  |  | destabilise the model or bias |
|  |  | predictions — confirmed empirically |
|  |  | by R² of 0.9983 and 0.9997 on the |
|  |  | two datasets. The VIF analysis is |
|  |  | retained to characterise feature |
|  |  | collinearity structure, not as an |
|  |  | exclusion criterion. |
| R5.5 | **Citation mismatch — Y. Li et al. (2023):** The | The reviewer is correct. Y. Li et |
|  | manuscript cites “Y. Li et al. (2023) [33]” in the | al. (2023) corresponds to reference |
|  | Discussion, but reference [33] appears to be a | [15] in the reference list (Li Y. et al., |
|  | different study (Lv M. et al., 2022) on wind energy | 2023. A TCN-Based Hybrid |
|  | forecasting. Authors should verify the correct | Forecasting Framework for |
|  | reference number for the intended Y. Li et | Hours-Ahead Utility-Scale PV |
|  | al. (2023) citation. | Forecasting, IEEE Transactions on |
|  |  | Smart Grid). The in-text citation has |
|  |  | been corrected from [33] to [15]. |
| R5.6 | **Citation mismatch — Shewalkar et al. (2019)** | The reviewer is correct. Reference |
|  | **[37]:** In Section 3.6.2, reference [37] (Shewalkar | [37] (Shewalkar et al., 2019, |
|  | et al., 2019, focused on speech recognition using | speech recognition) was |
|  | RNN/LSTM/GRU) is used to support a statement | erroneously cited in this context. |
|  | about medium- to long-term prediction for | The citation has been replaced with |
|  | integrated local energy systems. This reference is | reference [6] (Taheri et al., 2021. |
|  | irrelevant to energy systems. Reference [6] | Long-term planning of integrated |
|  | (Taheri et al., 2021) already in the manuscript | local energy systems using deep |
|  | directly supports this statement and should be | learning algorithms), which directly |
|  | used instead. | supports the statement about |
|  |  | medium- to long-term energy |
|  |  | prediction requirements. |

| R5.7  R5.8 | **Reference formatting:** Reference [15] contains an extraneous character “B” before the author name. Minor formatting inconsistencies should be corrected throughout the reference list.  **Equation (13) rendering:** Some subscript indices in Equation (13), particularly the subscript “i” in the Y terms, appear as “ı” (dotless i) rather than a standard mathematical subscript, suggesting a font embedding, Unicode, or PDF rendering artifact. | The extraneous character “B” before the author name in reference [15] has been removed. The reference now reads correctly as: Li Y. et al., 2023. A TCN-Based Hybrid Forecasting Framework…  The full reference list has also been reviewed for consistency.  We thank the reviewer for catching this. The cause was structural: the Ȳ mean term had the subscript ‘i’ nested inside the overbar accent element rather than outside it, causing the accent to render as a dotless-ı in certain PDF viewers. The equation has been corrected:  (1) the subscript ‘i’ has been removed from inside both mean |
| --- | --- | --- |
|  |  | terms (X̄ and Ȳ); (2) the summation |
|  |  | index has been standardised to i=1 |
|  |  | throughout (previously mixed |
|  |  | between t and i); (3) the |
|  |  | observation term Y_t has been |
|  |  | corrected to Y_i for consistency. |
|  |  | This equation is now numbered (9) |
|  |  | following the renumbering |
|  |  | described in R5.1. |
| R5.9 | **Manuscript structure mismatch:** The | The roadmap paragraph at the end |
|  | Introduction states the paper is structured in six | of the Introduction has been |
|  | sections including a standalone “state of the art” | rewritten to accurately reflect the |
|  | section. However, the current manuscript | actual structure of the manuscript. |
|  | contains four main sections: Introduction, | It now states that the paper is |
|  | Materials and Methods, Results and Discussion, | organised in four sections: (1) |
|  | and Conclusions. The roadmap paragraph should | Introduction (contextualisation and |
|  | be updated to accurately reflect the final structure. | related work), (2) Materials and |
|  |  | Methods (data, models, and |
|  |  | metrics), (3) Results and |
|  |  | Discussion, and (4) Conclusions. |
|  |  | The erroneous references to six |
|  |  | sections and a standalone “state of |
|  |  | the art” section have been |
|  |  | removed. |

R5.10	**Non-standard terminology:** The term “photovoltaic shells” is non-standard. Accepted terminology includes photovoltaic cells, modules, panels, or systems.

R5.11	**Typographical errors:** The manuscript contains typographical errors including apparent OCR/formatting artifacts (e.g., “201minu9” instead of “2019”) and misspellings (e.g., “miniute” instead of “minute”, appearing in both the main text figure caption and Appendix II). A careful proofreading pass is recommended.

R5.12	**Figure numbering inconsistency (main text vs. Appendix II):** The US/Spain solar energy forecast comparison appears as Fig. 16a/16b in the main text but as Fig. 13a/13b in Appendix II, causing all subsequent figures to be shifted by one number between the two locations. This directly affects Section 3.6.1, which refers to “Fig 16” while Appendix II shows a different figure under that number.

We thank the reviewer for this observation. All occurrences of “Photovoltaic Shell(s)” have been replaced with “photovoltaic plants” throughout the manuscript, including the abstract and the list of abbreviations.

Both errors have been corrected: the OCR artifact “201minu9” in the Introduction has been fixed to “2019”, and “miniute” has been corrected to “minute” in the figure caption (Fig. 14) and in the Appendix II figure list. A general proofreading pass has also been carried out across the manuscript. The figure list in Appendix II has been corrected to match the numbering used in the main text. The US/Spain solar energy forecast comparison is now listed as Fig 17a/17b in both locations (renumbered from the original Fig 16a/16b following the insertion of

the new Fig. 8 architecture diagram described under R6.4). The previously misaligned entries have been updated accordingly: Fig 14

(1-hour out-of-sample), Fig 15

(15-minute out-of-sample), and Fig 16 (Feature importance) now match the main text throughout.

| R5.13 | **nMAE inconsistency:** Table 2 and Figure 11 | The reviewer is correct. The value |
| --- | --- | --- |
|  | report an nMAE of 1.79% for the 15-minute | 1.96% in Section 3.6.1 was an error |
|  | multivariate TCN (Model 6), while Section 3.6.1 | from a previous version of the |
|  | states 1.96% when comparing against Bouquet et | manuscript; the correct value from |
|  | al. (2023). Authors should verify and ensure | Table 2 is 1.79% (Model 6, |
|  | consistency across all sections. | 15-minute multivariate TCN). |
|  |  | Beyond correcting the number, the |
|  |  | comparison paragraph in Section |
|  |  | 3.6.1 has been substantially |
|  |  | reworked. Bouquet et al. (2023) |
|  |  | forecast Global Horizontal |
|  |  | Irradiance (GHI, in W/m²) at a |
|  |  | single site in Switzerland — a |
|  |  | fundamentally different target |
|  |  | variable from national solar energy |
|  |  | production (MW), making a direct |
|  |  | nMAE comparison invalid |
|  |  | regardless of the numerical values. |
|  |  | The paragraph has therefore been |
|  |  | replaced with a comparison against |
|  |  | Cabello-López et al. (2023) [18], |
|  |  | the most directly comparable study, |
|  |  | which targets national-level solar |
|  |  | energy production in Spain using a |
|  |  | CNN-LSTM architecture (MAE |
|  |  | 67.32 MWh for 1-hour). The |
|  |  | revised paragraph reports our |
|  |  | correct nMAE values (4.10% for |
|  |  | 1-hour, 1.79% for 15-minute) and |
|  |  | explicitly notes that the 15-minute |
|  |  | national-level result has no direct |
|  |  | published benchmark. |
| R5.14 | **Undefined “profits” metric:** Figures 10 and 11 | We thank the reviewer for flagging |
|  | report a metric labeled “profits” (values 4,827.66 | this. Following the reviewer’s |
|  | and “inf” respectively) that is not defined or | suggestion, the “profits” indicator |
|  | discussed anywhere in the manuscript. The | has been removed from Figures 11 |
|  | occurrence of “inf” suggests a potential | and 12 (renumbered after the |
|  | computational issue. Authors should define and | insertion of the new Fig. 8 |
|  | explain this metric, or remove it if it is not part of | architecture diagram). The figures |
|  | the formal evaluation framework. | now display only the standard |
|  |  | forecast error metrics (sMAPE, |
|  |  | nMAE, correlation, success rate). |
|  |  | The simulated trading metric |
|  |  | definition that had been added to |
|  |  | the Error Metrics section has also |
|  |  | been removed. |

R5.15	**R² vs. correlation coefficient inconsistency (Table 1):** Table 1 has a column labeled “R²” but the text describes these as “correlation coefficients.” The reported values are not consistent with the standard relationship between Pearson r and R²: e.g., if 0.800787 is R², the implied r ≈ 0.895, which does not match the reported correlation value (~0.796). Authors should verify whether the column header or textual interpretation is incorrect and ensure consistency. Also: “a average correlation coefficient” should be “an average correlation coefficient.”

The reviewer is correct. The values in Table 1 are correlation coefficients (R²), meaning the (Pearson R)^2, which are coefficients of determination (R²). This is confirmed by cross-checking against the text: the value 0.796690 in the “R2 (train)” column for Madrid_G(i) matches exactly. However, in equation 9, the Pearson Coefficient is referred correctly as R and not R²).

The figure cited in the text (“an average correlation coefficient of 0.796”), which would only be consistent if the values are R² and not R (since √0.796690 ≈ 0.892, not 0.796). The equation ιis corrected as should be. Note that this table reports feature-selection correlations only; the R² values in Table 2 (model results) are correctly labelled as the coefficient of determination and are not affected by this correction. The grammatical error “a average” has also been corrected to “an average.”

# Reviewer #6

R6.1	**Title:** Remove the phrase “now-casting” from the title, as energy prediction is not acquired in real time. Suggested title: *Very short-term production prediction for photovoltaic plants using Artificial Intelligence and Temporal Convolution Networks.*

We thank the reviewer for this suggestion and have adopted a refined version of the proposed title. We also took the opportunity to correct two additional points: (i) “Convolution” has been replaced by the standard term “Convolutional”, as Temporal Convolutional

Networks (TCN) is the established designation in the literature (Bai et al., 2018); (ii) the reference to “Artificial Intelligence” has been removed as it is redundant — TCN is itself a form of artificial intelligence, and retaining both terms would be like naming both the category and the specific method. The revised title is: *Very short-term production prediction for photovoltaic plants using Temporal Convolutional Networks.*

R6.2	**Abstract — results clarity:** The abstract mentions “solar energy production can be successful on a short-term basis.” This should be explained in terms of the actual results obtained. Also, the abstract mentions “99.83% for the first case and 99.97% for the second case” — how many training iterations were used to achieve these accuracy percentages?

We thank the reviewer for these observations. The vague phrase “prediction of solar energy production can be successful on a short-term basis” has been replaced with a qualitative

characterisation of the key findings: the resolution-dependent performance pattern of the TCN relative to the linear regression baseline; the contrasting roles of TCN architecture and

weather-feature augmentation across the two forecasting horizons; and the sufficiency of past-only weather inputs as the principal methodological contribution. Regarding the “99.83% and 99.97%” figures, we

clarify that these were values of the coefficient of determination (R² 0.9983 andd 0.9997 respectively),

not classification accuracy scores obtained from training iterations. Rather than retaining them in the abstract with clarifying terminology, we have taken the reviewer’s comment as an opportunity to rewrite the abstract in a fully qualitative manner — removing specific percentages and R² values in favour of a concise description of the methodological approach and key findings. All quantitative results are reported in full in the Results and Discussion section and the Conclusions.

R6.3	**Introduction — Fig. 2 reference and literature survey:** (a) Fig. 2 lacks a reference for the values shown — a citation should be added. (b) The literature survey does not highlight accuracy and error metrics (such as MAE or MSE) for prior studies, nor does it identify which previous studies used the same dataset. A simpler

comparison with the most relevant prior studies is recommended, including key results from each referenced study to better demonstrate the contribution of the proposed system.

(a) The caption of Fig. 2 has been updated to include the data source: “Source: ENTSO-E [10]”. (b) A

comparative narrative paragraph has been added to the literature review section (just before the research questions), positioning this work relative to the two most relevant prior studies.

Cabello-López et al. (2023) [18] is the most directly comparable study, as it targets national-level solar energy production forecasting for Spain using a multivariate

CNN-LSTM across 1-hour, 24-hour, and 48-hour horizons, outperforming the official system operator forecast by 47.58% for the 1-hour case; however, their study does not address sub-hourly horizons. Li et al. (2023) [15] demonstrate that TCN

architectures outperform CNN-LSTM and other deep

learning models for utility-scale PV forecasting, but on individual farms rather than national aggregates. The paragraph makes explicit that the present work bridges these two directions — applying TCN at the national level and extending to the 15-minute resolution not previously addressed at this scale — and reports the achieved nMAE values (4.10% for 1-hour, 1.79% for

15-minute) for direct reference. A fuller numerical comparison with Cabello-López et al. (including their

67.32 MWh MAE) is provided in the Discussion section, where the

non-comparability of absolute errors across different dataset periods (2015–2018

vs. 2015–2021) is also noted.

R6.4	**Methods (Sections 2.5 and 2.6) — model diagram:** A detailed diagram of the proposed model illustrating the architecture design, network layers, interconnections, and internal model configurations should be included to provide a clearer understanding of the system structure and operational flow. The concept of the model should be clearly illustrated through an appropriate schematic diagram, minimizing excessive software-related details.

We thank the reviewer for this valuable suggestion. A three-panel architecture figure (Fig. 8) has been inserted at the end of Section 2.5: **(a)** the overall TCN pipeline, showing the input through L stacked TCN blocks with exponentially increasing dilation factors (d = 1, 2, …, 2^(L−1)) to the one-step forecast output; **(b)** a receptive-field illustration for k=2, L=3 (d=1, 2, 4), tracing contributing and non-contributing paths to the output neuron; and **(c)** the internal residual block structure — two dilated causal convolution layers with ReLU activations, a skip connection adding the block input

directly to F(x), and an optional 1×1 projection. Following the reviewer’s guidance to minimise

software-related details, the Python code snippets in Section 2.6 have been removed and replaced with concise prose descriptions; the scientific content is fully preserved.

R6.5	**Results and Discussion:** (a) Table 2: Review symbols and results more carefully for accuracy and consistency. (b) Section 3.5: Requires better organization and arrangement. (c) Section 3.6.1: Create a simplified comparison table between previous studies and the proposed system in terms of accuracy, error rates (MAE), datasets used, and methodology.

13

**(a) Table 2 headers:** All column headers have been corrected for consistency and clarity: smape → sMAPE (%), mae → MAE (MW) (units added), nmae → nMAE (%), success

→ Success rate (%) (clarifying it

is a directional accuracy measure), R2 → R², and header capitalization standardised throughout. The underlying numerical values were verified to be internally consistent: all improvement percentages in the summary rows were recomputed and confirmed correct (e.g.,

sMAPE improvement 53.88% = (45.38 − 20.93) / 45.38). **(b)**

**Section 3.5:** Several prose issues have been corrected: a typographical error (“12,228 pf the total VRAM”) has been fixed to “12,228 MB of the total VRAM”; the grammatical constructions “consider the 12,228…” and “this is result of” have been corrected to “considering that 12,228…” and “this is a result of”; “graphic card” has been corrected to “graphics card” throughout. The opening paragraph of the section has also been normalised from a List Paragraph to a Normal paragraph style for visual consistency with the surrounding text. **(c) Comparison table:** A structured comparison table (Table 6) has been added to

Section 3.6.1, immediately after the narrative comparison paragraphs and before the

architecture-vs-feature discussion. The table lists the six most relevant prior studies (Cabello-López et

al. [18], Y. Li et al. [15], Bhatt et

al. [31], Aman et al. [32], Abdelsattar et al. [33], and a Keras LSTM baseline) alongside this work, with columns for Method, Target/Scale, Forecast Horizon, Key Error Metric, and R²/r. A note is included in the table caption that direct numerical comparison is limited by methodological

R6.6	**Conclusions:** Update to include: (a) newly formulated theoretical contributions; (b) limitations of the study and prospects for future research; (c) key results in compact form re-emphasizing their significance; (d) how the article contributes new knowledge to the domain; (e) future work to motivate other researchers.

14

We thank the reviewer for this structured and valuable guidance. The Conclusions section has been fully rewritten to address all five elements within the

editor-mandated word limit. The revised section is organised in four paragraphs: (1) **Key results** — the multivariate TCN achieves R² of 0.9983 and 0.9997, nMAE of 4.10%

and 1.79%, and a 54% sMAPE reduction over the linear baseline at the hourly scale, obtained on national-level aggregated PV production for Spain; (2) **Theoretical contributions** — two principal findings are articulated:

(a) that past-only weather covariates (solar irradiance, sun height) are sufficient for accurate nowcasting without requiring future meteorological inputs, eliminating a circular dependency common in feature-augmented pipelines; and
(b) a resolution-dependent asymmetry showing that at 1-hour granularity the TCN architecture provides the dominant gain, while at 15-minute granularity weather covariates become proportionally more valuable as atmospheric variability disrupts temporal autocorrelation; (3) **Domain contribution** — feature selection showed that solar irradiance and sun height (with city-level correlations above 0.75 with the target) were the only covariates that contributed meaningfully, while temperature (r ≈ 0.37) was excluded; (4) **Limitations and future work** — the restriction to Spanish national aggregates and fixed training windows (2015–2018 for the hourly dataset; 2022–2023 for the 15-minute dataset) are acknowledged, and future directions are identified: probabilistic TCN variants for

uncertainty quantification, extended forecasting horizons, and transfer
