# Supplementary material for: Very short-term production prediction for photovoltaic plants using Temporal Convolutional Networks
Source: PLoS One. 2026 Jul 28;21(7):e0354512. doi: 10.1371/journal.pone.0354512 (PMC13411920; doi:10.1371/journal.pone.0354512)
Supplement: Attachment — Submitted filename: Response_to_Reviewers_R4.docx [file pone.0354512.s005.docx]

**Response to Reviewers — Fourth Revision (R4)**

**Manuscript:** Very short-term production prediction (now-casting) for photovoltaic plants using Arti- ficial Intelligence and Temporal Convolution Networks **Manuscript ID:** PONE-D-25-68879R2 **Jour- nal:** PLOS ONE **Deadline:** Aug 17 2026 11:59PM.

Dear Editor and Reviewers,

Thank you for the continued and careful review of our manuscript. Below we address each comment raised in this third round of review. Changes in the revised manuscript are highlighted in the track- changes version.

Kind Regards, The Authors

Dr. Loukas Samaras Prof. Elena García-Barriocanal Prof. Miguel-Angel Sicilia Lino González Gar- cía

# Editor Comment

\#	Editor Comment	How Addressed

**Revision 4**. Please use the space provided to explain your answers to the questions above. You may also include additional comments for the author, including concerns about dual publication, research ethics, or publication ethics. (Please upload your review as an attachment if it exceeds 20,000 characters)

All comments have been addressed in the 4^th^ revision

# Reviewer #5

R5.1	**The inconsistency between Table 1** and the surrounding text has not been fully resolved. The text describes the reported values as “correlation coefficients”, whereas Table 1 still labels the corresponding columns as “R²” and “R² (train)”. Pearson correlation coefficients and coefficients of determination are not the same quantity. If the values are Pearson correlation coefficients, the table headings should be changed accordingly. If they are R² values, the text should be revised and the interpretation should be corrected throughout the manuscript. This is an important point for statistical clarity.

R5.2	**My previous comment regarding the 15-minute dataset** still appears to be unaddressed. The manuscript should explicitly state whether the 15-minute solar-generation dataset contains missing values and, if so, how these were handled during preprocessing, model training, and evaluation. In addition, the reported end timestamp of the 15-minute dataset should be checked for consistency with the deposited data. This clarification is important for reproducibility.

R5.3	**Table 3 reports multiclass F1-score and	cross-validation accuracy.** Since the main task is a continuous forecasting/regression problem, the manuscript should more clearly explain how the classes were defined, what these classification-oriented metrics represent in this context, and why they are needed in addition to MAE, nMAE, sMAPE, and R/R²-type measures. If these metrics are not central to the forecasting evaluation, the authors may consider removing them or moving them to supplementary material.

The author is correct. The software we have built use the Pearson coefficient with the following python command: *cor=stats.pearsonr(df[df.columns[target_col]], df[col])*, where:

- stats is the python scipy library,

- pearsonr is the correlation coefficient

- Target_col is the energy column and

-col is every other column of the data frame. Therefore, Table 1 and in text reference are corrected

You absolutely correct. We mentioned in sub section 2.5 that 9 energy values are missing and we performed linear interpolation. We used the following command:

*df=df.resample(resamplimg_interval).interpolate(limit_direction='both'),* where:

•	df is the dataframe,

•	resampling_interval= 15 minutes

•	*limit_direction* =Interpolation direction: both, meaning forward and backward

These scores were asked by another reviewer and there were not in the original manuscript. However, we agree they are indeed useful for the forecast evaluation, as they provide a metric of accuracy (true or not), regardless the magnitude of the individual forecasted values towards the actual ones.

R5.4	**Possible inconsistency in the TCN architecture** description. Figure 8 and Section 2.5 describe dilated causal convolutions with increasing dilation factors, whereas the hyperparameter discussion indicates a dilation base of 1, which corresponds to regular causal convolution. Please clarify whether the final optimized model used dilated causal convolutions or regular causal convolutions, and revise the terminology accordingly.

R5.5	**The manuscript still needs careful proofreading and formatting correction before publication**. Examples include the metadata short title “using using AI”, typographical errors in the Data Availability Statement such as “oroginal” and “web sotes”, inconsistent singular/plural keyword usage, inconsistent decimal formatting in Table 5, and some remaining unclear or grammatically incomplete sentences. Numerical formatting should also be checked carefully, especially scientific notation/exponent formatting.

R5.6	**The authors should ensure that the funding	statement, acknowledgements, author affiliations, and competing interest statement are fully consistent with PLOS ONE requirements**. The submission form states that the authors received no specific funding and have no competing interests. However, the manuscript lists an author affiliated with In2AI and acknowledges support from this private corporation. The authors should clarify whether this support involved salaries, materials, infrastructure, software/data support, or only non-financial institutional support, and revise the Funding Statement and Competing Interest Statement accordingly. I leave the final assessment of this disclosure issue to the editorial office.

The reviewer is correct. The neural network architecture has been corrected according the hyperparameters. We provide a brief description of the 3 key elements:

A. Residual Block Pipeline (Block Architecture)

B. Multi-Layer 3D Tensor Flow (Three-Dimensional Data Stream)

C. Receptive Field Formula (Mathematical Foundation)

All corrections have been made, thank you very much

All comply with the journal’s guidelines. No funding was received In2A private corporation is specialized in such topics and provides us with their support and experience on the energy field.

**Reviewer #6.**

| R6.1 | Fig. 7 (Correlation Heatmap): Please improve the figure resolution and adjust its size to enhance readability and ensure that all labels and details are clearly visible. Also, Fig. no. 8. | Thank you very much for your comment. This heatmap has now been adjusted as much possible within the available page margins, since it contains a lot of information. |
| --- | --- | --- |
|  |  |  |
|  |  |  |
|  |  |  |
|  |  |  |
|  |  |  |
|  |  |  |
|  |  |  |
|  |  |  |
|  |  |  |
|  |  |  |
|  |  |  |
|  |  |  |
|  |  |  |
|  |  |  |
|  |  |  |
|  |  |  |
|  |  |  |
|  |  |  |
|  |  |  |
|  |  |  |
|  |  |  |
|  |  |  |
|  |  |  |
|  |  |  |
