# Supplementary material for: Very short-term production prediction for photovoltaic plants using Temporal Convolutional Networks
Source: PLoS One. 2026 Jul 28;21(7):e0354512. doi: 10.1371/journal.pone.0354512 (PMC13411920; doi:10.1371/journal.pone.0354512)
Supplement: Attachment — Submitted filename: Response_to_Reviewers_R5.docx [file pone.0354512.s006.docx]

**Response to Reviewers — Fifth Revision (R5)**

**Manuscript:** Very short-term production prediction (now-casting) for photovoltaic plants using Arti- ficial Intelligence and Temporal Convolution Networks **Manuscript ID:** PONE-D-25-68879R2 **Jour- nal:** PLOS ONE **Deadline:** Aug 20 2026 11:59PM.

Dear Editor and Reviewers,

Thank you for the continued and careful review of our manuscript. Below we address each comment raised in this third round of review. Changes in the revised manuscript are highlighted in the track- changes version.

Kind Regards, The Authors

Dr. Loukas Samaras Prof. Elena García-Barriocanal Prof. Miguel-Angel Sicilia Lino González Gar- cía

# Editor Comment

\#	Editor Comment	How Addressed

**Revision 5**. Please include the following items when submitting your revised manuscript:

- A letter that responds to each point raised by the academic editor and reviewer(s). You should upload this letter as a separate file labeled 'Response to Reviewers'.
- A marked-up copy of your manuscript that highlights changes made to the original version. You should upload this as a separate file labeled 'Revised Manuscript with Track Changes'.
- An unmarked version of your revised paper without tracked changes. You should upload this as a separate file labeled 'Manuscript'.

All comments have been addressed. All these documents have been prepared and uploaded

# Reviewer #5

R5.1	Typographical, metadata, and formatting issues:
**a) Please correct the remaining typographical, metadata, and formatting issues** wherever these fields are editable by the authors. If some of these items belong to the submission system metadata and are no longer editable by the authors, I kindly ask that they be checked during editorial or production processing.
In particular, the short title currently appears as “Very short-term production prediction for photovoltaic plants using using AI”. This should be corrected to a non-repetitive form, such as:
Very short-term production prediction for photovoltaic plants using Temporal Convolutional Networks
**b) In the Data Availability Statement**, please correct typographical/character issues such as “orιginal” and “web sιtes”. A corrected version could be:
Data can be found free on the following GitHub repository: <https://github.com/luxorsatellite/Darts_tcn/tree/main>. Alternatively, it can be downloaded from its original sources through the websites referred to in the manuscript.
**c) submission information**: I also noticed the phrase “A letetr...” in the submission information. If this field is editable and intended to remain part of the submission record, it should be corrected to “A letter...”. If it is only an internal submission-system note and not editable by the authors, this point can simply be ignored or handled by the editorial office.

All issues have been resolved in the metadata section of the on-line submission system, as follows:

a) Short title corrected as “Very short-term production prediction for photovoltaic plants using Temporal Convolutional Networks”.
b) Corrected as “Data can be found free on the following GitHub repository: <https://github.com/luxorsatellite/Darts_tcn/tree/main>. Alternatively, it can be downloaded from its original sources through the websites referred to in the manuscript.”
c) Corrected to “A letter...”.

R5.2	**Numerical formatting**
Please standardize numerical formatting in the tables, especially Table 4. For example, values such as 19.73, 289.10, 1.539,89, and 25,66 should follow a single decimal/thousand-separator convention throughout the manuscript.

R5.3	**Clarification of the final TCN architecture**.	The TCN description has improved, but there is still a minor terminology inconsistency. The manuscript describes TCNs in terms of dilated causal convolutions, while the optimized model is reported to use dilation base = 1, which corresponds to a regular causal convolution rather than an expanded dilated convolution.
Suggested correction:
Please add one clarifying sentence in the TCN architecture/hyperparameter section, for example:
Although the general TCN framework supports dilated causal convolutions, the optimized configuration selected in this study used a dilation base of 1. Therefore, the final implemented model corresponds to a causal convolutional TCN residual architecture without dilation expansion.
Alternatively, if the authors prefer to keep the term “dilated causal convolution”, please explicitly explain that dilation base = 1 is the special case of dilated convolution that reduces to regular causal convolution. This would avoid any possible misunderstanding about the final optimized architecture.

R5.4	**Clarification of Table 3 classification-oriented metrics.** Table 3 reports F1-score and cross-validation accuracy, but the main task of the paper is continuous solar-production forecasting/regression. It is therefore still not fully clear how the continuous target variable was converted into classes, what thresholds or bins were used, and what “multiclass” means in this forecasting context.

Table 4 corrected with single decimal numbers

Corrected as follows: “The general TCN framework supports dilated causal convolutions. Nevertheless, the optimized configuration selected in this study used a dilation base of 1. Therefore, the final implemented model corresponds to a causal convolutional TCN residual architecture without dilation expansion.”

The author is correct. These scores evaluate how well the models distinguish between different production-level categories. A multiclass score, for example, calculates the accuracy of forecast among different categories, which are substantial to calculate, especially to datasets with a high variance.

Suggested correction:
Please either remove these classification-oriented metrics from the main manuscript or move them to supplementary material if they are not central to the forecasting evaluation. If the authors wish to keep Table 3 in the main text, please add a short explanation such as:

For the supplementary classification-oriented evaluation reported in Table 3, the continuous solar-production values were discretized into [number] classes using [equal-width / quantile-based / domain-defined] thresholds. The F1-score and cross-validation accuracy therefore do not replace the main regression metrics, but provide an additional indication of how well the models distinguish between different production-level categories.

Please specify the exact number of classes and the class-definition method. This clarification would make the purpose of Table 3 much clearer for readers.

This should be done, because the success of prediction sometimes can be achieved and be high on the classes with higher values and not on the low values, resulting in a low or misleading average. In our data the classes are determined by the scale of the dataset. For instance, for the first dataset (hourly values), the minimum value is observed zero and the maximum 5,792. Therefore, we have created through Python's *scikit-learn* library six classes; from 0 to 1,000, 1,000-2,000 etc. until the last one 5,000-6,000.

The final measured accuracy is the global ratio of correctly predicted instances out of the total instances and can be determined by the following formula:

Accuracy = Total_Correct_Predictions / Total_Support, where:

Total_Correct_Predictions= the count of all samples where a forecasted class matched the true class exactly and

Total_Support= the total number of rows/

Therefore, we added these remarks in the metrics section.

Furthermore, under table 3 we briefly present the result of these categories: “As we can see from table 3, the accuracy scores of the neural net are all above 95.50% (0.9550) for the first dataset and over 94.60% for the second. This means that the precision is achieved through all six classes of data; both of low and high data values. In a more detail, we found out that the lower precision was observed in the data of range 3,000-4,000 MW (class #3: 87%) and the higher on the low ranges (0-1,000 MW, class 0: 99%). However, even for the last class #5 (5,000-6,000 MW), the accuracy is 97%.
