# Supplementary material for: Very short-term production prediction for photovoltaic plants using Temporal Convolutional Networks
Source: PLoS One. 2026 Jul 28;21(7):e0354512. doi: 10.1371/journal.pone.0354512 (PMC13411920; doi:10.1371/journal.pone.0354512)
Supplement: Peer review history

# Peer review: Decision Letter 0

**DOI:** 10.1371/journal.pone.0354512.r001

**Authors:** Dinesh Nishad

29 Jan 2026

PONE-D-25-68879

Very short-term production prediction (now-casting) for photovoltaic plants using Artificial Intelligence and Temporal Convolution Networks

PLOS One

Dear Dr. Samaras,

Thank you for submitting your manuscript to PLOS ONE. After careful consideration, we feel that it has merit but does not fully meet PLOS ONE’s publication criteria as it currently stands. Therefore, we invite you to submit a revised version of the manuscript that addresses the points raised during the review process.

Please submit your revised manuscript by Mar 15 2026 11:59PM. If you will need more time than this to complete your revisions, please reply to this message or contact the journal office at plosone@plos.org. When you're ready to submit your revision, log on to https://www.editorialmanager.com/pone/ and select the 'Submissions Needing Revision' folder to locate your manuscript file.

Please include the following items when submitting your revised manuscript:

- A letter that responds to each point raised by the academic editor and reviewer(s). You should upload this letter as a separate file labeled 'Response to Reviewers'.
- A marked-up copy of your manuscript that highlights changes made to the original version. You should upload this as a separate file labeled 'Revised Manuscript with Track Changes'.
- An unmarked version of your revised paper without tracked changes. You should upload this as a separate file labeled 'Manuscript'.

If you would like to make changes to your financial disclosure, please include your updated statement in your cover letter. Guidelines for resubmitting your figure files are available below the reviewer comments at the end of this letter.

If applicable, we recommend that you deposit your laboratory protocols in protocols.io to enhance the reproducibility of your results. Protocols.io assigns your protocol its own identifier (DOI) so that it can be cited independently in the future. For instructions see: https://journals.plos.org/plosone/s/submission-guidelines#loc-laboratory-protocols. Additionally, PLOS ONE offers an option for publishing peer-reviewed Lab Protocol articles, which describe protocols hosted on protocols.io. Read more information on sharing protocols at https://plos.org/protocols?utm_medium=editorial-email&utm_source=authorletters&utm_campaign=protocols.

We look forward to receiving your revised manuscript.

Kind regards,

Dinesh Kumar Nishad, PhD

Academic Editor

PLOS One

**Journal Requirements:**

When submitting your revision, we need you to address these additional requirements.

1. Please ensure that your manuscript meets PLOS ONE's style requirements, including those for file naming. The PLOS ONE style templates can be found at

https://journals.plos.org/plosone/s/file?id=wjVg/PLOSOne_formatting_sample_main_body.pdf and

https://journals.plos.org/plosone/s/file?id=ba62/PLOSOne_formatting_sample_title_authors_affiliations.pdf

2. Please note that PLOS One has specific guidelines on code sharing for submissions in which author-generated code underpins the findings in the manuscript. In these cases, we expect all author-generated code to be made available without restrictions upon publication of the work. Please review our guidelines at https://journals.plos.org/plosone/s/materials-and-software-sharing#loc-sharing-code and ensure that your code is shared in a way that follows best practice and facilitates reproducibility and reuse.

3. Thank you for stating the following in the Acknowledgments Section of your manuscript:

“We acknowledge the contribution of Computer Science Department the University of Alcalá de Henares and the private corporation ‘In2AI - Artificial Intelligence (AI) Consulting and Solutions’ for their support to complete this research.”

We note that you have provided funding information that is not currently declared in your Funding Statement. However, funding information should not appear in the Acknowledgments section or other areas of your manuscript. We will only publish funding information present in the Funding Statement section of the online submission form.

Please remove any funding-related text from the manuscript and let us know how you would like to update your Funding Statement. Currently, your Funding Statement reads as follows:

“The author(s) received no specific funding for this work.”

Additionally, because some of your funding information pertains to commercial funding we ask you to provide an updated Competing Interests statement, declaring all sources of commercial funding.

In your Competing Interests statement, please confirm that your commercial funding does not alter your adherence to PLOS ONE Editorial policies and criteria by including the following statement: "This does not alter our adherence to PLOS ONE policies on sharing data and materials.” as detailed online in our guide for authors  http://journals.plos.org/plosone/s/competing-interests.  If this statement is not true and your adherence to PLOS policies on sharing data and materials is altered, please explain how.

Please include the updated Competing Interests Statement and Funding Statement in your cover letter. We will change the online submission form on your behalf.

4. Please note that your Data Availability Statement is currently missing the repository name and or the DOI/accession number of each dataset OR a direct link to access each database. If your manuscript is accepted for publication, you will be asked to provide these details on a very short timeline. We therefore suggest that you provide this information now, though we will not hold up the peer review process if you are unable.

5. Thank you for stating the following in the Competing Interests section:

“The authors have declared that no competing interests exist.”

We note that one or more of the authors are employed by a commercial company:  In2AI - Artificial Intelligence (AI) Consulting and Solutions

1.  Please provide an amended Funding Statement declaring this commercial affiliation, as well as a statement regarding the Role of Funders in your study. If the funding organization did not play a role in the study design, data collection and analysis, decision to publish, or preparation of the manuscript and only provided financial support in the form of authors' salaries and/or research materials, please review your statements relating to the author contributions, and ensure you have specifically and accurately indicated the role(s) that these authors had in your study. You can update author roles in the Author Contributions section of the online submission form.

Please also include the following statement within your amended Funding Statement.

“The funder provided support in the form of salaries for authors [insert relevant initials], but did not have any additional role in the study design, data collection and analysis, decision to publish, or preparation of the manuscript. The specific roles of these authors are articulated in the ‘author contributions’ section.”

If your commercial affiliation did play a role in your study, please state and explain this role within your updated Funding Statement.

2. Please also provide an updated Competing Interests Statement declaring this commercial affiliation along with any other relevant declarations relating to employment, consultancy, patents, products in development, or marketed products, etc.

Within your Competing Interests Statement, please confirm that this commercial affiliation does not alter your adherence to all PLOS ONE policies on sharing data and materials by including the following statement: "This does not alter our adherence to  PLOS ONE policies on sharing data and materials.” (as detailed online in our guide for authors http://journals.plos.org/plosone/s/competing-interests) . If this adherence statement is not accurate and  there are restrictions on sharing of data and/or materials, please state these. Please note that we cannot proceed with consideration of your article until this information has been declared.

Please include both an updated Funding Statement and Competing Interests Statement in your cover letter. We will change the online submission form on your behalf.

6. Please include your full ethics statement in the ‘Methods’ section of your manuscript file. In your statement, please include the full name of the IRB or ethics committee who approved or waived your study, as well as whether or not you obtained informed written or verbal consent. If consent was waived for your study, please include this information in your statement as well.

7. Please ensure that you refer to Figures 3, 5, 6, 8 and 12 in your text as, if accepted, production will need this reference to link the reader to the figure.

8. We note that Figure 4 in your submission contain [map/satellite] images which may be copyrighted. All PLOS content is published under the Creative Commons Attribution License (CC BY 4.0), which means that the manuscript, images, and Supporting Information files will be freely available online, and any third party is permitted to access, download, copy, distribute, and use these materials in any way, even commercially, with proper attribution. For these reasons, we cannot publish previously copyrighted maps or satellite images created using proprietary data, such as Google software (Google Maps, Street View, and Earth). For more information, see our copyright guidelines: http://journals.plos.org/plosone/s/licenses-and-copyright.

We require you to either (1) present written permission from the copyright holder to publish these figures specifically under the CC BY 4.0 license, or (2) remove the figures from your submission:

1. You may seek permission from the original copyright holder of Figure 4 to publish the content specifically under the CC BY 4.0 license.

We recommend that you contact the original copyright holder with the Content Permission Form (http://journals.plos.org/plosone/s/file?id=7c09/content-permission-form.pdf) and the following text:

“I request permission for the open-access journal PLOS ONE to publish XXX under the Creative Commons Attribution License (CCAL) CC BY 4.0 (http://creativecommons.org/licenses/by/4.0/). Please be aware that this license allows unrestricted use and distribution, even commercially, by third parties. Please reply and provide explicit written permission to publish XXX under a CC BY license and complete the attached form.”

Please upload the completed Content Permission Form or other proof of granted permissions as an "Other" file with your submission.

In the figure caption of the copyrighted figure, please include the following text: “Reprinted from [ref] under a CC BY license, with permission from [name of publisher], original copyright [original copyright year].”

2. If you are unable to obtain permission from the original copyright holder to publish these figures under the CC BY 4.0 license or if the copyright holder’s requirements are incompatible with the CC BY 4.0 license, please either i) remove the figure or ii) supply a replacement figure that complies with the CC BY 4.0 license. Please check copyright information on all replacement figures and update the figure caption with source information. If applicable, please specify in the figure caption text when a figure is similar but not identical to the original image and is therefore for illustrative purposes only.

The following resources for replacing copyrighted map figures may be helpful:

USGS National Map Viewer (public domain): http://viewer.nationalmap.gov/viewer/

The Gateway to Astronaut Photography of Earth (public domain): http://eol.jsc.nasa.gov/sseop/clickmap/

Maps at the CIA (public domain): https://www.cia.gov/library/publications/the-world-factbook/index.html and https://www.cia.gov/library/publications/cia-maps-publications/index.html

NASA Earth Observatory (public domain): http://earthobservatory.nasa.gov/

Landsat: http://landsat.visibleearth.nasa.gov/

USGS EROS (Earth Resources Observatory and Science (EROS) Center) (public domain): http://eros.usgs.gov/#

Natural Earth (public domain): http://www.naturalearthdata.com/

9. If the reviewer comments include a recommendation to cite specific previously published works, please review and evaluate these publications to determine whether they are relevant and should be cited. There is no requirement to cite these works unless the editor has indicated otherwise.

**Additional Editor Comments:**

Please prepare a substantially revised version of the manuscript addressing all substantive points raised by the reviewers. You are encouraged to thoroughly evaluate, and where appropriate defend, your original reference choices rather than adding references suggested by reviewers; include only those you consider genuinely necessary and justified in your response letter. Please also examine Reviewer 3 comments carefully and correct only those that are factually or methodologically sound, as some uploaded remarks appear to be erroneous or not applicable to your work

[Note: HTML markup is below. Please do not edit.]

Reviewers' comments:

Reviewer's Responses to Questions

**Comments to the Author**

1. Is the manuscript technically sound, and do the data support the conclusions?

The manuscript must describe a technically sound piece of scientific research with data that supports the conclusions. Experiments must have been conducted rigorously, with appropriate controls, replication, and sample sizes. The conclusions must be drawn appropriately based on the data presented.

Reviewer #1: Partly

Reviewer #2: Yes

Reviewer #3: Partly

**********

2. Has the statistical analysis been performed appropriately and rigorously?

Reviewer #1: No

Reviewer #2: Yes

Reviewer #3: No

**********

3. Have the authors made all data underlying the findings in their manuscript fully available?

The PLOS Data policy requires authors to make all data underlying the findings described in their manuscript fully available without restriction, with rare exception (please refer to the Data Availability Statement in the manuscript PDF file). The data should be provided as part of the manuscript or its supporting information, or deposited to a public repository. For example, in addition to summary statistics, the data points behind means, medians and variance measures should be available. If there are restrictions on publicly sharing data—e.g. participant privacy or use of data from a third party—those must be specified.

Reviewer #1: No

Reviewer #2: Yes

Reviewer #3: Yes

**********

4. Is the manuscript presented in an intelligible fashion and written in standard English?

PLOS ONE does not copyedit accepted manuscripts, so the language in submitted articles must be clear, correct, and unambiguous. Any typographical or grammatical errors should be corrected at revision, so please note any specific errors here.

Reviewer #1: No

Reviewer #2: Yes

Reviewer #3: Yes

**********

5. Review Comments to the Author

Please use the space provided to explain your answers to the questions above. You may also include additional comments for the author, including concerns about dual publication, research ethics, or publication ethics. (Please upload your review as an attachment if it exceeds 20,000 characters)

Reviewer #1: 1. The writing of the paper needs a lot of improvement in terms of grammar, spelling, and presentation.

2. What is the novelty of this paper?

3. Add a flowchart to show what is done in this study.

4. Most Figures are blurred.

5. The mathematics used throughout the article is still not very strict. Please try to update and illustrate some elements in the mathematical model that are not defined very strictly.

6. The result part is week, results and discussion should be better explained.

7. Revise the conclusions and make it concise, (Better to write the main outcomes)

8. Include some Key Suggested References:

https://doi.org/10.1038/s41598-025-16828-2

https://doi.org/10.1038/s41598-025-14908-x

https://doi.org/10.1038/s41598-025-09101-z

https://doi.org/10.1038/s41598-025-98607-7

DOI: 10.1109/MEPCON58725.2023.10462371

10.1007/978-3-031-43243-9_7

10.1109/EIConRus.2018.8317170

Reviewer #2: The topic is relevant for grid integration of renewables; however, several methodological and reporting issues should be addressed to ensure technical rigor, fair benchmarking, and reproducibility.

1. Please clarify the exact backtesting protocol (rolling/expanding window, retraining frequency, forecast origin) and ensure strict chronological separation (no future covariate leakage via scaling/encoders).

2. You claim irradiance variables are used as “past variables” so no need to forecast weather, but the now-casting setup still needs a clear statement of what is known at prediction time (e.g., at time t, are G(i) and H_sun available up to t only, and prediction is for t+1?).

3. Linear regression alone is too weak for a time-series benchmark. Add classical TS models (e.g., persistence/naïve, SARIMA/ARIMAX, ETS), and modern ML baselines (XGBoost/LightGBM with lag features). Comprehensive Assessment of Solar Agrivoltaics Potential: Systematic Review and Techno-Economic Assessment Modeling Toward Sustainable Food and Energy Production

4. Table 2 shows TCN_univariate performing worse than LR for 15-min (higher sMAPE/MAE, lower success), while multivariate improves. Explain why the univariate TCN degrades (architecture mismatch, insufficient receptive field, scaling, seasonality settings), and include an ablation on receptive field / kernel size / dilation and lag length.

5. Covariates are selected primarily by correlation. Please justify scientifically (and avoid target leakage) by reporting: (a) correlation computed only on training data, (b) lagged correlation/cross-correlation vs lead/lag structure, and (c) multicollinearity handling (many city variables likely correlated).

6. The manuscript discusses MAPE instability for low solar outputs, but still uses MAPE in discussion; also please verify the sMAPE equation formatting/denominator and report confidence intervals (bootstrapped) for MAE/nMAE/sMAPE.

7. Provide full hyperparameters (input length, output length, batch size, epochs actually used after early stopping, optimizer settings, weight decay, random seeds) and code for Darts pipeline. Energy-exergy and environ-economic (4E) analysis of heat storage-based single-slope solar stills integrated with solar air heater

8. “Data can be found free by the appropriate websites…” is not sufficient.

Reviewer #3: 1. The manuscript reports strong aggregate performance metrics; however, class-wise precision, recall, and F1-score are not sufficiently analyzed. Given the multi-class nature of the problem, per-class evaluation and error distribution analysis are required to assess model reliability.

2. The dataset preparation process is described, but statistical details regarding class balance before and after preprocessing are missing. A quantitative summary of class distribution and its impact on training should be included.

3. The model evaluation relies on a single train–validation–test split. The absence of cross-validation or repeated experiments limits confidence in generalization and robustness of the reported results.

4. Comparisons with existing approaches are based on reported results from the literature rather than evaluation under identical experimental conditions. This limits the strength of comparative performance claims.

5. The proposed architecture has a high parameter count, yet no analysis of inference time, computational complexity, or memory footprint is provided, which is critical for assessing real-world applicability.

6. While data augmentation techniques are applied, their individual contribution to performance improvement is not analyzed. An ablation study would strengthen the methodological justification.

7. Reproducibility is insufficiently addressed. The manuscript does not clearly state whether the code, trained models, and configuration details are publicly available, which is essential for PLOS ONE compliance.

8. The discussion section focuses mainly on performance gains and lacks analysis of failure cases or limitations, such as visually similar classes or adverse imaging conditions, which are important for a balanced scientific assessment.

9. The literature review can be strengthened by including additional references related to deep learning and neural network–based approaches.

doi.org/10.1504/IJEHV.2023.132034

https://doi.org/10.1080/01430750.2024.2315485

https://doi.org/10.4271/12-08-04-0036

https://doi.org/10.1504/IJICA.2025.148630

**********

6. PLOS authors have the option to publish the peer review history of their article (what does this mean?). If published, this will include your full peer review and any attached files.

If you choose “no”, your identity will remain anonymous but your review may still be made public.

**Do you want your identity to be public for this peer review?**  For information about this choice, including consent withdrawal, please see our Privacy Policy.

Reviewer #1: No

Reviewer #2: No

Reviewer #3: No

**********

[NOTE: If reviewer comments were submitted as an attachment file, they will be attached to this email and accessible via the submission site. Please log into your account, locate the manuscript record, and check for the action link "View Attachments". If this link does not appear, there are no attachment files.]

To ensure your figures meet our technical requirements, please review our figure guidelines: https://journals.plos.org/plosone/s/figures

You may also use PLOS’s free figure tool, NAAS, to help you prepare publication quality figures: https://journals.plos.org/plosone/s/figures#loc-tools-for-figure-preparation.

NAAS will assess whether your figures meet our technical requirements by comparing each figure against our figure specifications.

# Author comment: Author response to Decision Letter 1

**DOI:** 10.1371/journal.pone.0354512.r002

25 Feb 2026

Thank you all for the very interesting and useful comments. We have created a major revision of the manuscript according to your comments, rewriting and enriching most parts especially after the first Section of Introduction. You can find our responses to all comments in the letter named 'Response to Reviewers.docx'

Attachment Submitted filename: Response to Reviewers.docx

# Peer review: Decision Letter 1

**DOI:** 10.1371/journal.pone.0354512.r003

**Authors:** Dinesh Nishad

18 Apr 2026

PONE-D-25-68879R1

Very short-term production prediction (now-casting) for photovoltaic plants using Artificial Intelligence and Temporal Convolution Networks

PLOS One

Dear Dr. Samaras,

Thank you for submitting your manuscript to PLOS ONE. After careful consideration, we feel that it has merit but does not fully meet PLOS ONE’s publication criteria as it currently stands. Therefore, we invite you to submit a revised version of the manuscript that addresses the points raised during the review process.

Please submit your revised manuscript by Jun 02 2026 11:59PM. If you will need more time than this to complete your revisions, please reply to this message or contact the journal office at plosone@plos.org. When you're ready to submit your revision, log on to https://www.editorialmanager.com/pone/ and select the 'Submissions Needing Revision' folder to locate your manuscript file.

Please include the following items when submitting your revised manuscript:

- A letter that responds to each point raised by the academic editor and reviewer(s). You should upload this letter as a separate file labeled 'Response to Reviewers'.
- A marked-up copy of your manuscript that highlights changes made to the original version. You should upload this as a separate file labeled 'Revised Manuscript with Track Changes'.
- An unmarked version of your revised paper without tracked changes. You should upload this as a separate file labeled 'Manuscript'.

If you would like to make changes to your financial disclosure, please include your updated statement in your cover letter. Guidelines for resubmitting your figure files are available below the reviewer comments at the end of this letter.

If applicable, we recommend that you deposit your laboratory protocols in protocols.io to enhance the reproducibility of your results. Protocols.io assigns your protocol its own identifier (DOI) so that it can be cited independently in the future. For instructions see: https://journals.plos.org/plosone/s/submission-guidelines#loc-laboratory-protocols. Additionally, PLOS ONE offers an option for publishing peer-reviewed Lab Protocol articles, which describe protocols hosted on protocols.io. Read more information on sharing protocols at https://plos.org/protocols?utm_medium=editorial-email&utm_source=authorletters&utm_campaign=protocols.

As the corresponding author, your ORCID iD is verified in the submission system and will appear in the published article. PLOS supports the use of ORCID, and we encourage all coauthors to register for an ORCID iD and use it as well. Please encourage your coauthors to verify their ORCID iD within the submission system before final acceptance, as unverified ORCID iDs will not appear in the published article. *Only*  the individual author can complete the verification step; PLOS staff *cannot*  verify ORCID iDs on behalf of authors.

We look forward to receiving your revised manuscript.

Kind regards,

Dinesh Kumar Nishad, PhD

Academic Editor

PLOS One

Journal Requirements:

1. If the reviewer comments include a recommendation to cite specific previously published works, please review and evaluate these publications to determine whether they are relevant and should be cited. There is no requirement to cite these works unless the editor has indicated otherwise.

Additional Editor Comments:

1. Rewrite Abstract in a single para and remove heading Background:, Objective:, Methods: and Results

2. Replace Figure 1, Figure 2 with large size Axis fonts

3. Fig. 4 Flowchart is not clearly visible so replace it , and text should be in black colour.

4. Replace Fig. 8, Fig. 12a, Fig. 12b, and Fig. 14 with large axis font

5. Rewrite section 4 conclusion in 2-3 para, 200-300 words avoid numbering

6. Refer Already published research papers in Plos one for better presentation of this work.

[Note: HTML markup is below. Please do not edit.]

Reviewer's Responses to Questions

**Comments to the Author**

1. If the authors have adequately addressed your comments raised in a previous round of review and you feel that this manuscript is now acceptable for publication, you may indicate that here to bypass the “Comments to the Author” section, enter your conflict of interest statement in the “Confidential to Editor” section, and submit your "Accept" recommendation.

Reviewer #4: (No Response)

**********

2. Is the manuscript technically sound, and do the data support the conclusions?

The manuscript must describe a technically sound piece of scientific research with data that supports the conclusions. Experiments must have been conducted rigorously, with appropriate controls, replication, and sample sizes. The conclusions must be drawn appropriately based on the data presented.

Reviewer #4: Partly

**********

3. Has the statistical analysis been performed appropriately and rigorously?

Reviewer #4: No

**********

4. Have the authors made all data underlying the findings in their manuscript fully available?

The PLOS Data policy requires authors to make all data underlying the findings described in their manuscript fully available without restriction, with rare exception (please refer to the Data Availability Statement in the manuscript PDF file). The data should be provided as part of the manuscript or its supporting information, or deposited to a public repository. For example, in addition to summary statistics, the data points behind means, medians and variance measures should be available. If there are restrictions on publicly sharing data—e.g. participant privacy or use of data from a third party—those must be specified.

Reviewer #4: Yes

**********

5. Is the manuscript presented in an intelligible fashion and written in standard English?

PLOS ONE does not copyedit accepted manuscripts, so the language in submitted articles must be clear, correct, and unambiguous. Any typographical or grammatical errors should be corrected at revision, so please note any specific errors here.

Reviewer #4: No

**********

6. Review Comments to the Author

Please use the space provided to explain your answers to the questions above. You may also include additional comments for the author, including concerns about dual publication, research ethics, or publication ethics. (Please upload your review as an attachment if it exceeds 20,000 characters)

Reviewer #4: 1. Title & Abstract

• The title is clear and representative of the study.

• The abstract states the problem, data source, methods, and some numerical results.

• However, the abstract uses vague or potentially misleading wording such as “regression score” without defining whether this refers to correlation or coefficient of determination.

• The abstract would be stronger if it emphasized the practical significance of the forecast for grid balancing and clarified the exact metric names.

2. Introduction

• The problem is clearly important, and the manuscript explains why short-term solar forecasting matters for energy balancing.

• The introduction is broad and contains useful context, but it is too long and sometimes drifts into general energy background not essential to the research question.

• The research questions are explicitly stated, which is a strength.

• The motivation is relevant, but the research gap should be framed more sharply against prior solar forecasting studies rather than against very broad energy-forecasting literature.

3. Related Work

• The paper cites a substantial body of prior work, including solar forecasting, wind forecasting, and deep learning approaches.

• However, the comparison is often descriptive rather than critical.

• The literature review mixes directly relevant studies with less relevant material from NLP, speech, and generic convolutional-network applications, which weakens focus.

• The manuscript should compare more directly against recent solar forecasting studies using comparable targets, horizons, and datasets.

4. Methodology

• This is the most serious weakness of the paper.

• The section labeled as TCN includes LSTM-style equations for input, forget, cell, and output gates. These are not TCN equations and should not be used to describe a temporal convolutional network.

• The manuscript later mentions dilated convolutions, kernel size, and dilation base, but the overall architectural description remains conceptually inconsistent.

• The reported hyperparameters are useful, but reproducibility is still incomplete because the forecasting protocol, retraining schedule, scaler fitting procedure, and exact backtesting design are not fully specified.

• The “real-world scenario” is especially problematic because it states that all data are used simultaneously as training and validation, which is not a valid evaluation setup.

• Covariate selection is based mainly on correlation, while strong multicollinearity is acknowledged but not handled rigorously. The response that the network can learn the best lag structure is not a sufficient methodological justification.

5. Results

• Results are presented numerically in tables and visually in plots, which is good.

• The hourly multivariate TCN improves clearly over the reported linear-regression baseline, especially in sMAPE and nMAE.

• However, the 15-minute univariate TCN performs worse than the linear-regression baseline, while the multivariate TCN gives only modest gains. This deserves a fuller explanation.

• The benchmark is too weak: comparison only against linear regression is not enough to support strong claims about superiority of AI or deep learning.

• Metrics are also not fully satisfactory. The manuscript uses MAE, nMAE, MAPE/sMAPE, “success,” F1 score, and cross-validation “accuracy,” but the classification-style metrics are insufficiently justified for a regression problem.

• The manuscript also refers to Pearson correlation as R2, which is conceptually confusing.

6. Discussion

• The discussion identifies some practical implications of improved short-term forecasting.

• However, several claims are overstated. The manuscript argues that its results are better than most previous studies, but many of those comparisons are not like-for-like because they involve different targets, geographies, horizons, and covariates.

• Some parts of the discussion move too far from the core contribution, especially the subsection on “electricity optimization and efficiency,” which reads more like a general expansion than a focused interpretation of the present results.

• Limitations are mentioned, but they should be discussed more directly: dependence on selected weather variables, potential leakage issues, limited baseline set, and uncertain generalizability beyond Spain.

7. Conclusion

• The conclusion restates the main findings and links them back to the research questions.

• However, it repeats some over-strong claims and should be moderated.

• A stronger conclusion would summarize the real contribution more carefully: country-level PV now-casting with public data, evidence that past weather covariates help, and a need for stronger future validation and benchmarking.

8. References

• The paper includes both older foundational references and some recent works.

• That said, the reference list is unevenly curated. Some citations are only indirectly relevant, and the literature review should be tightened around recent solar-energy forecasting studies.

• Formatting and consistency also need attention.

Originality

The application of TCN-style forecasting to aggregated national PV generation is potentially valuable, especially with both hourly and 15-minute public data. The idea is not wholly novel at the algorithmic level, but the application context has some originality.

Contribution

The paper’s main contribution is practical rather than theoretical: it explores whether short-term country-scale PV now-casting is feasible and whether weather covariates improve it. That contribution is useful, but it is currently weakened by poor benchmarking and unclear methodology.

Technical quality

Currently not strong enough. The incorrect TCN description, weak validation design, unclear regression/classification metrics, and limited baselines all reduce confidence in the technical rigor.

Clarity

The manuscript is difficult to read in places because of grammar issues, inconsistent terminology, and formatting artifacts.

Organization

The paper has a recognizable structure, but numbering, section flow, and presentation need cleanup. Discussion material is not always well separated from results.

Main weaknesses

• Incorrect TCN methodological description.

• Weak baseline comparison.

• Unclear temporal validation and possible leakage concerns.

• Poorly justified use of classification-style accuracy metrics for regression.

• Overstated comparative claims.

• Serious formatting and manuscript-hygiene problems.

**********

7. PLOS authors have the option to publish the peer review history of their article (what does this mean?). If published, this will include your full peer review and any attached files.

If you choose “no”, your identity will remain anonymous but your review may still be made public.

**Do you want your identity to be public for this peer review?**  For information about this choice, including consent withdrawal, please see our Privacy Policy.

Reviewer #4: **Yes:** Waleed A. Ali

**********

[NOTE: If reviewer comments were submitted as an attachment file, they will be attached to this email and accessible via the submission site. Please log into your account, locate the manuscript record, and check for the action link "View Attachments". If this link does not appear, there are no attachment files.]

To ensure your figures meet our technical requirements, please review our figure guidelines: https://journals.plos.org/plosone/s/figures

You may also use PLOS’s free figure tool, NAAS, to help you prepare publication quality figures: https://journals.plos.org/plosone/s/figures#loc-tools-for-figure-preparation.

NAAS will assess whether your figures meet our technical requirements by comparing each figure against our figure specifications.

# Author comment: Author response to Decision Letter 2

**DOI:** 10.1371/journal.pone.0354512.r004

14 May 2026

Dear Editor and Reviewers,

Thank you all for your comments. We have created a second revision of the manuscript, by modifying and rewriting to achieve the best outcome. You can see our responses to comments in the Response to Reviewers.docx file.

Attachment Submitted filename: Response_to_Reviewers_auresp_2.docx

# Peer review: Decision Letter 2

**DOI:** 10.1371/journal.pone.0354512.r005

**Authors:** Dinesh Nishad

27 May 2026

PONE-D-25-68879R2

Very short-term production prediction (now-casting) for photovoltaic plants using Artificial Intelligence and Temporal Convolution Networks

PLOS One

Dear Dr. Samaras,

Thank you for submitting your manuscript to PLOS ONE. After careful consideration, we feel that it has merit but does not fully meet PLOS ONE’s publication criteria as it currently stands. Therefore, we invite you to submit a revised version of the manuscript that addresses the points raised during the review process.

Please submit your revised manuscript by Jul 11 2026 11:59PM. If you will need more time than this to complete your revisions, please reply to this message or contact the journal office at plosone@plos.org. When you're ready to submit your revision, log on to https://www.editorialmanager.com/pone/ and select the 'Submissions Needing Revision' folder to locate your manuscript file.

Please include the following items when submitting your revised manuscript:

- A letter that responds to each point raised by the academic editor and reviewer(s). You should upload this letter as a separate file labeled 'Response to Reviewers'.
- A marked-up copy of your manuscript that highlights changes made to the original version. You should upload this as a separate file labeled 'Revised Manuscript with Track Changes'.
- An unmarked version of your revised paper without tracked changes. You should upload this as a separate file labeled 'Manuscript'.

If you would like to make changes to your financial disclosure, please include your updated statement in your cover letter. Guidelines for resubmitting your figure files are available below the reviewer comments at the end of this letter.

If applicable, we recommend that you deposit your laboratory protocols in protocols.io to enhance the reproducibility of your results. Protocols.io assigns your protocol its own identifier (DOI) so that it can be cited independently in the future. For instructions see: https://journals.plos.org/plosone/s/submission-guidelines#loc-laboratory-protocols. Additionally, PLOS ONE offers an option for publishing peer-reviewed Lab Protocol articles, which describe protocols hosted on protocols.io. Read more information on sharing protocols at https://plos.org/protocols?utm_medium=editorial-email&utm_source=authorletters&utm_campaign=protocols.

As the corresponding author, your ORCID iD is verified in the submission system and will appear in the published article. PLOS supports the use of ORCID, and we encourage all coauthors to register for an ORCID iD and use it as well. Please encourage your coauthors to verify their ORCID iD within the submission system before final acceptance, as unverified ORCID iDs will not appear in the published article. *Only*  the individual author can complete the verification step; PLOS staff *cannot*  verify ORCID iDs on behalf of authors.

We look forward to receiving your revised manuscript.

Kind regards,

Dinesh Kumar Nishad, PhD

Academic Editor

PLOS One

Journal Requirements:

If the reviewer comments include a recommendation to cite specific previously published works, please review and evaluate these publications to determine whether they are relevant and should be cited. There is no requirement to cite these works unless the editor has indicated otherwise.

Additional Editor Comments:

At this stage fulfill and answer all the reviewers' comments carefully.

[Note: HTML markup is below. Please do not edit.]

Reviewers' comments:

Reviewer's Responses to Questions

**Comments to the Author**

1. If the authors have adequately addressed your comments raised in a previous round of review and you feel that this manuscript is now acceptable for publication, you may indicate that here to bypass the “Comments to the Author” section, enter your conflict of interest statement in the “Confidential to Editor” section, and submit your "Accept" recommendation.

Reviewer #5: (No Response)

Reviewer #6: (No Response)

**********

2. Is the manuscript technically sound, and do the data support the conclusions?

The manuscript must describe a technically sound piece of scientific research with data that supports the conclusions. Experiments must have been conducted rigorously, with appropriate controls, replication, and sample sizes. The conclusions must be drawn appropriately based on the data presented.

Reviewer #5: Partly

Reviewer #6: Yes

**********

3. Has the statistical analysis been performed appropriately and rigorously?

Reviewer #5: No

Reviewer #6: Yes

**********

4. Have the authors made all data underlying the findings in their manuscript fully available?

The PLOS Data policy requires authors to make all data underlying the findings described in their manuscript fully available without restriction, with rare exception (please refer to the Data Availability Statement in the manuscript PDF file). The data should be provided as part of the manuscript or its supporting information, or deposited to a public repository. For example, in addition to summary statistics, the data points behind means, medians and variance measures should be available. If there are restrictions on publicly sharing data—e.g. participant privacy or use of data from a third party—those must be specified.

Reviewer #5: Yes

Reviewer #6: Yes

**********

5. Is the manuscript presented in an intelligible fashion and written in standard English?

PLOS ONE does not copyedit accepted manuscripts, so the language in submitted articles must be clear, correct, and unambiguous. Any typographical or grammatical errors should be corrected at revision, so please note any specific errors here.

Reviewer #5: No

Reviewer #6: Yes

**********

6. Review Comments to the Author

Please use the space provided to explain your answers to the questions above. You may also include additional comments for the author, including concerns about dual publication, research ethics, or publication ethics. (Please upload your review as an attachment if it exceeds 20,000 characters)

Reviewer #5: This manuscript was read with genuine interest and careful attention throughout. The study addresses a practically important and timely research problem: the very short-term forecasting of aggregated solar photovoltaic energy production at the national level using Temporal Convolutional Networks and weather covariates. The work is built on a substantial volume of real-world data sourced from ENTSO-E, and the overall research design - including the use of both hourly and 15-minute intervals, the incorporation of exogenous meteorological variables, and the real-world out-of-sample scenario - reflects a genuine effort to bridge methodological rigor with practical applicability. The comparative analysis across multiple model configurations and the transparent reporting of hardware requirements further add to the value of the work. The topic is well-suited for the scope of PLOS ONE, and the manuscript shows clear potential. However, a number of issues were identified during the review that require the authors' attention before the manuscript can be considered for publication. These are detailed below.

1-

Section 2.5 (“The TCN model”) contains equations that correspond to a standard LSTM architecture rather than a Temporal Convolutional Network (TCN). Specifically, Equations (3)-(8) define recurrent gating mechanisms and hidden/cell state transitions ((i_t, f_t, o_t, c_t, h_t)), which are characteristic of LSTM-based recurrent neural networks.

However, TCN architectures are fundamentally convolution-based and typically rely on causal convolutions, dilated convolutions, receptive fields, and residual connections instead of recurrent hidden states and gating operations. Therefore, the current formulation creates substantial methodological ambiguity regarding the actual implemented architecture.

The authors should carefully revise this section to ensure consistency between the theoretical description and the implemented model. In particular, the manuscript should include equations and architectural explanations specifically related to TCN operations and clarify whether Equations (3)-(8) are included for conceptual background purposes only, or whether recurrent components were actually incorporated into the implemented architecture.

2-

Equation (12), intended to define the Symmetric Mean Absolute Percentage Error (sMAPE), appears to be inconsistent with the standard formulation commonly used in the forecasting literature.

The denominator is written as:

[(X_i - Y_i)/2]

whereas the conventional sMAPE definition generally uses:

[(|X_i| + |Y_i|)/2]

This distinction is important because replacing the average magnitude term with the prediction error term substantially changes the mathematical behavior of the metric. In the current formulation, both the numerator and denominator are derived from the same difference quantity, causing the expression to simplify toward nearly constant values (approximately ±2 for each term depending on the sign of the error), rather than functioning as a normalized symmetric percentage error metric.

As written, Equation (12) therefore does not appear to preserve the intended properties of sMAPE as a scale-independent forecasting evaluation metric.

The authors should carefully verify whether this issue is only a typographical error in the manuscript or whether the metric was also implemented in this form during the experimental evaluation, since this could directly affect the validity and interpretation of the reported forecasting results.

3-

The interpretation of Table 2 would benefit from further clarification regarding the sources of performance gains across different model configurations.

Although the TCN_multivariate model consistently outperforms the Linear Regression baseline across both forecasting horizons, the TCN_univariate model performs worse than the baseline in the 15-minute forecasting scenario (sMAPE: 33.52 vs 21.04; MAE: 82.55 vs 69.41).

This suggests that the observed improvements are primarily driven by the inclusion of external weather-related covariates (e.g., irradiance and solar position features), rather than by the TCN architecture itself. In other words, the contribution of exogenous variables appears to dominate the predictive gain.

The manuscript would therefore benefit from a clearer separation between (i) the effect of the neural network architecture and (ii) the effect of multivariate feature augmentation, in order to avoid attributing performance improvements solely to the TCN model. A more controlled ablation-style discussion would strengthen the validity of the conclusions.

4-

The discussion of Variance Inflation Factor (VIF) values requires clarification from a methodological standpoint.

Several predictor variables exhibit very high VIF values, including Madrid_H_sun (VIF = 95.734) and Seville_H_sun (VIF = 104.226), which are typically considered indicative of severe multicollinearity in standard statistical practice.

However, the manuscript interprets these values as “relatively low” based on a comparison with the mean magnitude of the dependent variable (solar energy generation). This approach is not consistent with standard interpretations of VIF, as VIF is a dimensionless measure of multicollinearity among explanatory variables and is not defined relative to the scale of the dependent variable.

Clarification is recommended regarding whether this interpretation is intentional and based on a specific methodological framework or whether a conventional VIF interpretation (e.g., commonly used thresholds such as 5 or 10) should be applied.

5-

Several citation inconsistencies have been identified in the manuscript that require careful verification and correction.

First, the manuscript cites “Y. Li et al. (2023) [33]” in the context of solar energy forecasting within the Discussion section; however, the corresponding reference entry [33] appears to describe a different study (Lv M. et al., 2022) focused on wind energy forecasting. This suggests a potential mismatch between the in-text citation and the reference list. It is possible that the intended citation was another work by Y. Li et al. (2023), which may be listed under a different reference number in the manuscript.

Second, in Section 3.6.2, the statement regarding medium- to long-term prediction requirements for integrated local energy systems is supported by reference [37] (Shewalkar et al., 2019). However, this reference focuses on performance evaluation of deep neural networks applied to speech recognition using RNN/LSTM/GRU models and has no relevance to energy systems or forecasting applications. Notably, a more appropriate reference already present in the manuscript - reference [6] (Taheri et al., 2021), which explicitly addresses long-term planning of integrated local energy systems - appears to directly support this statement.

Additionally, minor formatting issues were observed in the reference list (e.g., reference [15], where an extraneous character “B” appears before the author name), which should be corrected for consistency.

The authors are therefore requested to carefully verify all citations throughout the manuscript to ensure full consistency between in-text references and the bibliography, particularly in the related work and comparative analysis sections.

6-

Equation (13) may contain a minor rendering or typography issue in the PDF version. In the displayed equation, some subscript indices, particularly the subscript “i” in the Y terms, visually appear as the character “ı” (dotless i) rather than a standard mathematical subscript index. This behavior persists even when zooming into the PDF, suggesting a possible font embedding, Unicode, or PDF rendering artifact.

7-

The manuscript organization paragraph in the Introduction appears to be inconsistent with the final structure of the paper.

The text states that the paper is structured in six sections, including the Introduction, and refers to a separate “state of the art” section. However, the current manuscript appears to contain four main sections: Introduction, Materials and Methods, Results and Discussion, and Conclusions.

A distinct standalone “state of the art” section is not clearly identifiable in the revised version, suggesting that the roadmap paragraph may not have been updated following manuscript revisions.

The authors are therefore encouraged to revise the introductory organization paragraph to accurately reflect the final structure and section numbering of the manuscript to ensure consistency and clarity.

8-

The term “photovoltaic shells” appears to be non-standard in the context of photovoltaic energy systems. In the solar energy literature, commonly accepted terminology includes photovoltaic cells, modules, panels, or systems.

9-

A careful proofreading pass is recommended prior to publication, as the manuscript contains several typographical errors, including apparent OCR or formatting artifacts (e.g., “201minu9” instead of “2019”) and misspellings (e.g., “miniute” instead of “minute”, appearing in both the main text figure caption and the Appendix II list).

10-

There is a systematic inconsistency between the figure numbering in the main text and the figure list in Appendix II. In the main text, the US/Spain solar energy forecast comparison appears as Fig. 16a and Fig. 16b, while Appendix II lists these same figures as Fig. 13a and Fig. 13b. As a consequence, all subsequent figures are shifted by one number between the two locations: for example, what the main text calls Fig. 13 (1-hour out-of-sample prediction) appears as Fig. 14 in Appendix II, and what the main text calls Fig. 15 (Feature importance) appears as Fig. 16 in Appendix II. This discrepancy has a direct practical consequence: in Section 3.6.1, the text refers to ‘Fig 16’ to show the US/Spain forecast comparison, whereas a reader consulting Appendix II would find an entirely different figure (Feature importance) under that number. The figure list in Appendix II appears not to have been updated following the repositioning of these figures during revision.

11-

An internal numerical inconsistency has been identified in the reported nMAE values for the 15-minute multivariate TCN model (Model 6).

Table 2 and Figure 11 report an nMAE value of 1.79%, whereas Section 3.6.1 states a value of 1.96% when comparing the results against Bouquet et al. (2023).

The authors are kindly requested to verify this discrepancy and ensure consistency across all sections of the manuscript. If the difference arises from distinct evaluation settings or subsets of data, this should be explicitly clarified in the text.

12-

A lack of definition has been identified for the metric labeled “profits” appearing in Figures 10 and 11.

While Figure 10 reports a value of 4,827.66 and Figure 11 reports “inf”, this metric is not defined or discussed anywhere in the manuscript, including the methodology, evaluation metrics section, or results discussion. As a result, the interpretation of this quantity is unclear to the reader.

Moreover, the occurrence of an infinite value (“inf”) suggests a potential computational or scaling issue that is not addressed in the manuscript.

The authors are therefore requested to clarify the definition, computation method, and relevance of this metric, or remove it if it is not part of the formal evaluation framework.

13-

An inconsistency has been identified between the statistical notation used in Table 1 and its interpretation in Section 2.4.

Table 1 reports a column labeled “R²”, whereas the corresponding text describes these values as “correlation coefficients” or “average correlation coefficients.” However, the numerical values reported in the table do not appear consistent with the standard mathematical relationship between Pearson correlation (r) and the coefficient of determination (R²).

For example, if the reported value 0.800787 corresponds to R², the implied correlation coefficient would be approximately √0.800787 ≈ 0.895, which does not align with the reported correlation value (~0.796). Similar inconsistencies are observed for other entries.

This suggests that either the table header is mislabeled (R² vs. correlation coefficient), or the textual interpretation of the results is inconsistent with the reported statistics. The authors should carefully verify and correct the statistical notation throughout the manuscript to ensure consistency between tables, formulas, and narrative descriptions.

Additionally, minor language revision is recommended (e.g., “a average correlation coefficient” -> “an average correlation coefficient”).

14-

A minor inconsistency was identified between the Abstract and Table 2 regarding the reported improvements for the 15-minute forecasting scenario.

In the Abstract, the manuscript states that the forecasts were improved by “3.33% and 10.50% respectively” following the mention of “MAE and sMAPE”. However, Table 2 reports the 15-minute improvements as 3.33% for sMAPE, 10.60% for MAE, and 10.50% for nMAE.

Therefore, the ordering and metric correspondence in the Abstract appear inconsistent with the values presented in Table 2. In addition, the Abstract refers to “MAE”, whereas the table separately reports both MAE and nMAE, which are different evaluation metrics.

The authors are kindly encouraged to verify and align the reported values, metric names, and ordering between the Abstract and Table 2 for consistency and clarity.

15-

The 15-minute dataset available in the GitHub repository contains nine missing values in the solar generation column. These missing entries are not acknowledged anywhere in the manuscript, and no information is provided regarding how they were handled during preprocessing, model training, or evaluation. In the interest of reproducibility and methodological transparency, the authors are encouraged to briefly describe the missing value handling procedure applied to this dataset.

Additionally, a minor discrepancy was noted regarding the reported end timestamp of the 15-minute dataset: the manuscript states "31.12.2023 23:45" whereas the last recorded entry in the deposited file is 2023-12-31 23:00:00. The authors may wish to verify this point for completeness.

Reviewer #6: TITLE AND ABSTRACT - Provide comments and recommendations for the title and abstract.

Title:

o I prefer to remove the phrase "now-casting" from the manuscript's title, as the energy prediction not acquire at real time.

o Instead, I'd prefer the title: Very short-term production prediction for photovoltaic plants using Artificial Intelligence and Temporal Convolution Networks.

Abstract:

o The abstract mentions " of solar energy production can be successful on a short-term basis." Can you explain it in terms of the results you obtained?

o The abstract mentions “99.83% for the first case and 99.97% for the second case” ، How many training times did you use to achieve this accuracy percentage?

INTRODUCTION - Provide comments and recommendations

Introduction:

o Fig. 2: What is the reference of these values within Fig.2? it is preferable to cite the Ref. used for this study.

o Literature Survey: In previous studies, why did the authors not highlight the accuracy and error metrics (such as MAE or MSE) of those studies? Which previous studies used the same dataset as the proposed manuscript? A simpler comparison with the most relevant previous studies related to the proposed work in this manuscript is recommended. In addition, some key results from each referenced study should be included to better demonstrate the contribution and performance of the proposed system

METHOD - Provide comments and recommendations.

section (2.5 and 2.6):

It would be beneficial to include a detailed diagram of the proposed model illustrating the architecture design, network layers, interconnections, and internal model configurations to provide a clearer understanding of the system structure and operational flow. )To minimize excessive software-related details and emphasize the essential design equations, the concept of the proposed model should be clearly illustrated through an appropriate schematic diagram.)

RESULTS AND DISCUSSION - Provide comments and recommendations for the research results and discussion.

Results:

o Table 2. The summary results:

Please review the symbols and the results presented in the table more carefully to ensure their accuracy and consistency.

o Section 3.5:

It requires better organization and arrangement.

o 3.6.1. Energy Forecast

I recommend creating a simplified comparison table between the previous studies and the proposed system in terms of accuracy, error rates (MAE), utilized datasets, and the methodology proposed by the researchers.

CONCLUSIONS - Provide comments and recommendations for conclusions.

- Update the conclusion to include the newly formulated theoretical contributions;

- Mention the limitations of the study and prospects for future research;

- Summarize the key results in a compact form and re-emphasize their significance;

- Summarize how the article contributes to new knowledge in the domain.

- Add future work to motivate other researchers to continue the research.

**********

7. PLOS authors have the option to publish the peer review history of their article (what does this mean?). If published, this will include your full peer review and any attached files.

If you choose “no”, your identity will remain anonymous but your review may still be made public.

**Do you want your identity to be public for this peer review?**  For information about this choice, including consent withdrawal, please see our Privacy Policy.

Reviewer #5: No

Reviewer #6: **Yes:** Zahraa Talib

**********

[NOTE: If reviewer comments were submitted as an attachment file, they will be attached to this email and accessible via the submission site. Please log into your account, locate the manuscript record, and check for the action link "View Attachments". If this link does not appear, there are no attachment files.]

To ensure your figures meet our technical requirements, please review our figure guidelines: https://journals.plos.org/plosone/s/figures

You may also use PLOS’s free figure tool, NAAS, to help you prepare publication quality figures: https://journals.plos.org/plosone/s/figures#loc-tools-for-figure-preparation.

NAAS will assess whether your figures meet our technical requirements by comparing each figure against our figure specifications.

# Author comment: Author response to Decision Letter 3

**DOI:** 10.1371/journal.pone.0354512.r006

29 Jun 2026

We have uploaded a separate file named "Response_to_Reviewers_R3.docx"

Attachment Submitted filename: Response_to_Reviewers_R3.docx

# Peer review: Decision Letter 3

**DOI:** 10.1371/journal.pone.0354512.r007

**Authors:** Dinesh Nishad

3 Jul 2026

PONE-D-25-68879R3

Very short-term production prediction for photovoltaic plants using Temporal Convolutional Networks

PLOS One

Dear Dr. Samaras,

Thank you for submitting your manuscript to PLOS ONE. After careful consideration, we feel that it has merit but does not fully meet PLOS ONE’s publication criteria as it currently stands. Therefore, we invite you to submit a revised version of the manuscript that addresses the points raised during the review process.

Please submit your revised manuscript by Aug 17 2026 11:59PM. If you will need more time than this to complete your revisions, please reply to this message or contact the journal office at plosone@plos.org. When you're ready to submit your revision, log on to https://www.editorialmanager.com/pone/ and select the 'Submissions Needing Revision' folder to locate your manuscript file.

Please include the following items when submitting your revised manuscript:

- A letter that responds to each point raised by the academic editor and reviewer(s). You should upload this letter as a separate file labeled 'Response to Reviewers'.
- A marked-up copy of your manuscript that highlights changes made to the original version. You should upload this as a separate file labeled 'Revised Manuscript with Track Changes'.
- An unmarked version of your revised paper without tracked changes. You should upload this as a separate file labeled 'Manuscript'.

If you would like to make changes to your financial disclosure, please include your updated statement in your cover letter. Guidelines for resubmitting your figure files are available below the reviewer comments at the end of this letter.

If applicable, we recommend that you deposit your laboratory protocols in protocols.io to enhance the reproducibility of your results. Protocols.io assigns your protocol its own identifier (DOI) so that it can be cited independently in the future. For instructions see: https://journals.plos.org/plosone/s/submission-guidelines#loc-laboratory-protocols. Additionally, PLOS ONE offers an option for publishing peer-reviewed Lab Protocol articles, which describe protocols hosted on protocols.io. Read more information on sharing protocols at https://plos.org/protocols?utm_medium=editorial-email&utm_source=authorletters&utm_campaign=protocols.

As the corresponding author, your ORCID iD is verified in the submission system and will appear in the published article. PLOS supports the use of ORCID, and we encourage all coauthors to register for an ORCID iD and use it as well. Please encourage your coauthors to verify their ORCID iD within the submission system before final acceptance, as unverified ORCID iDs will not appear in the published article. *Only*  the individual author can complete the verification step; PLOS staff *cannot*  verify ORCID iDs on behalf of authors.

We look forward to receiving your revised manuscript.

Kind regards,

Dinesh Kumar Nishad, PhD

Academic Editor

PLOS One

**Journal Requirements:**

If the reviewer comments include a recommendation to cite specific previously published works, please review and evaluate these publications to determine whether they are relevant and should be cited. There is no requirement to cite these works unless the editor has indicated otherwise.

[Note: HTML markup is below. Please do not edit.]

Reviewers' comments:

Reviewer's Responses to Questions

**Comments to the Author**

1. If the authors have adequately addressed your comments raised in a previous round of review and you feel that this manuscript is now acceptable for publication, you may indicate that here to bypass the “Comments to the Author” section, enter your conflict of interest statement in the “Confidential to Editor” section, and submit your "Accept" recommendation.

Reviewer #5: (No Response)

Reviewer #6: All comments have been addressed

**********

2. Is the manuscript technically sound, and do the data support the conclusions?

The manuscript must describe a technically sound piece of scientific research with data that supports the conclusions. Experiments must have been conducted rigorously, with appropriate controls, replication, and sample sizes. The conclusions must be drawn appropriately based on the data presented.

Reviewer #5: Partly

Reviewer #6: Yes

**********

3. Has the statistical analysis been performed appropriately and rigorously?

Reviewer #5: No

Reviewer #6: Yes

**********

4. Have the authors made all data underlying the findings in their manuscript fully available?

The PLOS Data policy requires authors to make all data underlying the findings described in their manuscript fully available without restriction, with rare exception (please refer to the Data Availability Statement in the manuscript PDF file). The data should be provided as part of the manuscript or its supporting information, or deposited to a public repository. For example, in addition to summary statistics, the data points behind means, medians and variance measures should be available. If there are restrictions on publicly sharing data—e.g. participant privacy or use of data from a third party—those must be specified.

Reviewer #5: Yes

Reviewer #6: Yes

**********

5. Is the manuscript presented in an intelligible fashion and written in standard English?

PLOS ONE does not copyedit accepted manuscripts, so the language in submitted articles must be clear, correct, and unambiguous. Any typographical or grammatical errors should be corrected at revision, so please note any specific errors here.

Reviewer #5: No

Reviewer #6: Yes

**********

6. Review Comments to the Author

Please use the space provided to explain your answers to the questions above. You may also include additional comments for the author, including concerns about dual publication, research ethics, or publication ethics. (Please upload your review as an attachment if it exceeds 20,000 characters)

Reviewer #5: I would like to thank the authors for their substantial revision of the manuscript. The revised version is clearly improved compared with the previous submission. In particular, the title and abstract are more focused, the previous LSTM-related equations have been replaced with a more appropriate TCN description, the sMAPE formula has been corrected, the distinction between architectural contribution and weather-feature contribution is now clearer, the discussion of the 15-minute forecasting case is more balanced, and the comparison with previous studies and the conclusion section have been improved.

However, I do not yet consider the manuscript ready for acceptance, because several important issues remain unresolved or insufficiently clarified.

1. The inconsistency between Table 1 and the surrounding text has not been fully resolved. The text describes the reported values as “correlation coefficients”, whereas Table 1 still labels the corresponding columns as “R²” and “R² (train)”. Pearson correlation coefficients and coefficients of determination are not the same quantity. If the values are Pearson correlation coefficients, the table headings should be changed accordingly. If they are R² values, the text should be revised and the interpretation should be corrected throughout the manuscript. This is an important point for statistical clarity.

2. My previous comment regarding the 15-minute dataset still appears to be unaddressed. The manuscript should explicitly state whether the 15-minute solar-generation dataset contains missing values and, if so, how these were handled during preprocessing, model training, and evaluation. In addition, the reported end timestamp of the 15-minute dataset should be checked for consistency with the deposited data. This clarification is important for reproducibility.

3. The manuscript should clarify a possible inconsistency in the TCN architecture description. Figure 8 and Section 2.5 describe dilated causal convolutions with increasing dilation factors, whereas the hyperparameter discussion indicates a dilation base of 1, which corresponds to regular causal convolution. Please clarify whether the final optimized model used dilated causal convolutions or regular causal convolutions, and revise the terminology accordingly.

4. Table 3 reports multiclass F1-score and cross-validation accuracy. Since the main task is a continuous forecasting/regression problem, the manuscript should more clearly explain how the classes were defined, what these classification-oriented metrics represent in this context, and why they are needed in addition to MAE, nMAE, sMAPE, and R/R²-type measures. If these metrics are not central to the forecasting evaluation, the authors may consider removing them or moving them to supplementary material.

5. The manuscript still needs careful proofreading and formatting correction before publication. Examples include the metadata short title “using using AI”, typographical errors in the Data Availability Statement such as “oroginal” and “web sotes”, inconsistent singular/plural keyword usage, inconsistent decimal formatting in Table 5, and some remaining unclear or grammatically incomplete sentences. Numerical formatting should also be checked carefully, especially scientific notation/exponent formatting.

6. The authors should ensure that the funding statement, acknowledgements, author affiliations, and competing interest statement are fully consistent with PLOS ONE requirements. The submission form states that the authors received no specific funding and have no competing interests. However, the manuscript lists an author affiliated with In2AI and acknowledges support from this private corporation. The authors should clarify whether this support involved salaries, materials, infrastructure, software/data support, or only non-financial institutional support, and revise the Funding Statement and Competing Interest Statement accordingly. I leave the final assessment of this disclosure issue to the editorial office.

Overall, I appreciate the authors’ considerable effort in revising the manuscript. The study addresses an important and practically relevant problem in national-level photovoltaic production forecasting, and the revised manuscript is much stronger than the previous version. Nevertheless, the remaining issues above concern statistical notation, reproducibility, model-description consistency, manuscript hygiene, and disclosure transparency. I therefore recommend a targeted major revision before the manuscript can be considered for acceptance.

Reviewer #6: The authors are sincerely thanked for their careful revision of the manuscript and for adequately addressing the reviewers' comments and suggestions. Their efforts have significantly improved the quality and clarity of the paper. Only one minor comment remains and should be carefully addressed before publication. I look forward to seeing more high-quality research from the authors in this important and promising research area.

Fig. 7 (Correlation Heatmap): Please improve the figure resolution and adjust its size to enhance readability and ensure that all labels and details are clearly visible. Also, Fig. no. 8.

**********

7. PLOS authors have the option to publish the peer review history of their article (what does this mean?). If published, this will include your full peer review and any attached files.

If you choose “no”, your identity will remain anonymous but your review may still be made public.

**Do you want your identity to be public for this peer review?**  For information about this choice, including consent withdrawal, please see our Privacy Policy.

Reviewer #5: No

Reviewer #6: **Yes:** Zahraa Talib

**********

[NOTE: If reviewer comments were submitted as an attachment file, they will be attached to this email and accessible via the submission site. Please log into your account, locate the manuscript record, and check for the action link "View Attachments". If this link does not appear, there are no attachment files.]

To ensure your figures meet our technical requirements, please review our figure guidelines: https://journals.plos.org/plosone/s/figures

You may also use PLOS’s free figure tool, NAAS, to help you prepare publication quality figures: https://journals.plos.org/plosone/s/figures#loc-tools-for-figure-preparation.

NAAS will assess whether your figures meet our technical requirements by comparing each figure against our figure specifications.

# Author comment: Author response to Decision Letter 4

**DOI:** 10.1371/journal.pone.0354512.r008

4 Jul 2026

A letetr with the filename "Response_to_Reviewers_R4.docx" was uploaded, containing our response to all comments

Attachment Submitted filename: Response_to_Reviewers_R4.docx

# Peer review: Decision Letter 4

**DOI:** 10.1371/journal.pone.0354512.r009

**Authors:** Dinesh Nishad

6 Jul 2026

PONE-D-25-68879R4

Very short-term production prediction for photovoltaic plants using Temporal Convolutional Networks

PLOS One

Dear Dr. Samaras,

Thank you for submitting your manuscript to PLOS ONE. After careful consideration, we feel that it has merit but does not fully meet PLOS ONE’s publication criteria as it currently stands. Therefore, we invite you to submit a revised version of the manuscript that addresses the points raised during the review process.

Please submit your revised manuscript by Aug 20 2026 11:59PM. If you will need more time than this to complete your revisions, please reply to this message or contact the journal office at plosone@plos.org. When you're ready to submit your revision, log on to https://www.editorialmanager.com/pone/ and select the 'Submissions Needing Revision' folder to locate your manuscript file.

Please include the following items when submitting your revised manuscript:

- A letter that responds to each point raised by the academic editor and reviewer(s). You should upload this letter as a separate file labeled 'Response to Reviewers'.
- A marked-up copy of your manuscript that highlights changes made to the original version. You should upload this as a separate file labeled 'Revised Manuscript with Track Changes'.
- An unmarked version of your revised paper without tracked changes. You should upload this as a separate file labeled 'Manuscript'.

If you would like to make changes to your financial disclosure, please include your updated statement in your cover letter. Guidelines for resubmitting your figure files are available below the reviewer comments at the end of this letter.

If applicable, we recommend that you deposit your laboratory protocols in protocols.io to enhance the reproducibility of your results. Protocols.io assigns your protocol its own identifier (DOI) so that it can be cited independently in the future. For instructions see: https://journals.plos.org/plosone/s/submission-guidelines#loc-laboratory-protocols. Additionally, PLOS ONE offers an option for publishing peer-reviewed Lab Protocol articles, which describe protocols hosted on protocols.io. Read more information on sharing protocols at https://plos.org/protocols?utm_medium=editorial-email&utm_source=authorletters&utm_campaign=protocols.

As the corresponding author, your ORCID iD is verified in the submission system and will appear in the published article. PLOS supports the use of ORCID, and we encourage all coauthors to register for an ORCID iD and use it as well. Please encourage your coauthors to verify their ORCID iD within the submission system before final acceptance, as unverified ORCID iDs will not appear in the published article. *Only*  the individual author can complete the verification step; PLOS staff *cannot*  verify ORCID iDs on behalf of authors.

We look forward to receiving your revised manuscript.

Kind regards,

Dinesh Kumar Nishad, PhD

Academic Editor

PLOS One

Journal Requirements:

If the reviewer comments include a recommendation to cite specific previously published works, please review and evaluate these publications to determine whether they are relevant and should be cited. There is no requirement to cite these works unless the editor has indicated otherwise.

Please review your reference list to ensure that it is complete and correct. If you have cited papers that have been retracted, please include the rationale for doing so in the manuscript text, or remove these references and replace them with relevant current references. Any changes to the reference list should be mentioned in the rebuttal letter that accompanies your revised manuscript. If you need to cite a retracted article, indicate the article’s retracted status in the References list and also include a citation and full reference for the retraction notice.

[Note: HTML markup is below. Please do not edit.]

Reviewers' comments:

Reviewer's Responses to Questions

**Comments to the Author**

1. If the authors have adequately addressed your comments raised in a previous round of review and you feel that this manuscript is now acceptable for publication, you may indicate that here to bypass the “Comments to the Author” section, enter your conflict of interest statement in the “Confidential to Editor” section, and submit your "Accept" recommendation.

Reviewer #5: (No Response)

**********

2. Is the manuscript technically sound, and do the data support the conclusions?

The manuscript must describe a technically sound piece of scientific research with data that supports the conclusions. Experiments must have been conducted rigorously, with appropriate controls, replication, and sample sizes. The conclusions must be drawn appropriately based on the data presented.

Reviewer #5: Yes

**********

3. Has the statistical analysis been performed appropriately and rigorously?

Reviewer #5: Yes

**********

4. Have the authors made all data underlying the findings in their manuscript fully available?

The PLOS Data policy requires authors to make all data underlying the findings described in their manuscript fully available without restriction, with rare exception (please refer to the Data Availability Statement in the manuscript PDF file). The data should be provided as part of the manuscript or its supporting information, or deposited to a public repository. For example, in addition to summary statistics, the data points behind means, medians and variance measures should be available. If there are restrictions on publicly sharing data—e.g. participant privacy or use of data from a third party—those must be specified.

Reviewer #5: Yes

**********

5. Is the manuscript presented in an intelligible fashion and written in standard English?

PLOS ONE does not copyedit accepted manuscripts, so the language in submitted articles must be clear, correct, and unambiguous. Any typographical or grammatical errors should be corrected at revision, so please note any specific errors here.

Reviewer #5: Yes

**********

6. Review Comments to the Author

Please use the space provided to explain your answers to the questions above. You may also include additional comments for the author, including concerns about dual publication, research ethics, or publication ethics. (Please upload your review as an attachment if it exceeds 20,000 characters)

Reviewer #5: I would like to sincerely thank the authors for their careful revision of the manuscript. The revised version is much improved, and I appreciate the effort made to address the previous comments. At this stage, I do not see major scientific obstacles to publication. However, I recommend a minor revision to resolve a few remaining presentation and clarification issues before acceptance.

1. Typographical, metadata, and formatting issues

Please correct the remaining typographical, metadata, and formatting issues wherever these fields are editable by the authors. If some of these items belong to the submission system metadata and are no longer editable by the authors, I kindly ask that they be checked during editorial or production processing.

In particular, the short title currently appears as “Very short-term production prediction for photovoltaic plants using using AI”. This should be corrected to a non-repetitive form, such as:

Very short-term production prediction for photovoltaic plants using Temporal Convolutional Networks

In the Data Availability Statement, please correct typographical/character issues such as “orginal” and “web stes”. A corrected version could be:

Data can be found free on the following GitHub repository: https://github.com/luxorsatellite/Darts_tcn/tree/main. Alternatively, it can be downloaded from its original sources through the websites referred to in the manuscript.

I also noticed the phrase “A letetr...” in the submission information. If this field is editable and intended to remain part of the submission record, it should be corrected to “A letter...”. If it is only an internal submission-system note and not editable by the authors, this point can simply be ignored or handled by the editorial office.

2. Numerical formatting

Please standardize numerical formatting in the tables, especially Table 4. For example, values such as 19.73, 289.10, 1.539,89, and 25,66 should follow a single decimal/thousand-separator convention throughout the manuscript.

3.Clarification of the final TCN architecture

The TCN description has improved, but there is still a minor terminology inconsistency. The manuscript describes TCNs in terms of dilated causal convolutions, while the optimized model is reported to use dilation base = 1, which corresponds to a regular causal convolution rather than an expanded dilated convolution.

Suggested correction:

Please add one clarifying sentence in the TCN architecture/hyperparameter section, for example:

Although the general TCN framework supports dilated causal convolutions, the optimized configuration selected in this study used a dilation base of 1. Therefore, the final implemented model corresponds to a causal convolutional TCN residual architecture without dilation expansion.

Alternatively, if the authors prefer to keep the term “dilated causal convolution”, please explicitly explain that dilation base = 1 is the special case of dilated convolution that reduces to regular causal convolution. This would avoid any possible misunderstanding about the final optimized architecture.

4.Clarification of Table 3 classification-oriented metrics

Table 3 reports F1-score and cross-validation accuracy, but the main task of the paper is continuous solar-production forecasting/regression. It is therefore still not fully clear how the continuous target variable was converted into classes, what thresholds or bins were used, and what “multiclass” means in this forecasting context.

Suggested correction:

Please either remove these classification-oriented metrics from the main manuscript or move them to supplementary material if they are not central to the forecasting evaluation. If the authors wish to keep Table 3 in the main text, please add a short explanation such as:

For the supplementary classification-oriented evaluation reported in Table 3, the continuous solar-production values were discretized into [number] classes using [equal-width / quantile-based / domain-defined] thresholds. The F1-score and cross-validation accuracy therefore do not replace the main regression metrics, but provide an additional indication of how well the models distinguish between different production-level categories.

Please specify the exact number of classes and the class-definition method. This clarification would make the purpose of Table 3 much clearer for readers.

Overall, I appreciate the authors’ substantial improvements and constructive responses during the revision process. The manuscript is now much stronger, and I wish the authors continued success with this valuable research direction. After the minor points above are addressed, I believe the manuscript can be considered suitable for publication.

**********

7. PLOS authors have the option to publish the peer review history of their article (what does this mean?). If published, this will include your full peer review and any attached files.

If you choose “no”, your identity will remain anonymous but your review may still be made public.

**Do you want your identity to be public for this peer review?**  For information about this choice, including consent withdrawal, please see our Privacy Policy.

Reviewer #5: No

**********

[NOTE: If reviewer comments were submitted as an attachment file, they will be attached to this email and accessible via the submission site. Please log into your account, locate the manuscript record, and check for the action link "View Attachments". If this link does not appear, there are no attachment files.]

To ensure your figures meet our technical requirements, please review our figure guidelines: https://journals.plos.org/plosone/s/figures

You may also use PLOS’s free figure tool, NAAS, to help you prepare publication quality figures: https://journals.plos.org/plosone/s/figures#loc-tools-for-figure-preparation.

NAAS will assess whether your figures meet our technical requirements by comparing each figure against our figure specifications.

# Author comment: Author response to Decision Letter 5

**DOI:** 10.1371/journal.pone.0354512.r010

7 Jul 2026

A A letter with the filename "Response_to_Reviewers_R5.docx" has been uploaded, containing all responses to the reviewers' comments

Attachment Submitted filename: Response_to_Reviewers_R5.docx

# Peer review: Decision Letter 5

**DOI:** 10.1371/journal.pone.0354512.r011

**Authors:** Dinesh Nishad

10 Jul 2026

Very short-term production prediction for photovoltaic plants using Temporal Convolutional Networks

PONE-D-25-68879R5

Dear Dr. Samaras,

We’re pleased to inform you that your manuscript has been judged scientifically suitable for publication and will be formally accepted for publication once it meets all outstanding technical requirements.

Within one week, you’ll receive an e-mail detailing the required amendments. When these have been addressed, you’ll receive a formal acceptance letter and your manuscript will be scheduled for publication.

An invoice will be generated when your article is formally accepted. Please note, if your institution has a publishing partnership with PLOS and your article meets the relevant criteria, all or part of your publication costs will be covered. Please make sure your user information is up-to-date by logging into Editorial Manager at Editorial Manager® and clicking the ‘Update My Information' link at the top of the page. For questions related to billing, please contact billing support.

If your institution or institutions have a press office, please notify them about your upcoming paper to help maximize its impact. If they’ll be preparing press materials, please inform our press team as soon as possible -- no later than 48 hours after receiving the formal acceptance. Your manuscript will remain under strict press embargo until 2 pm Eastern Time on the date of publication. For more information, please contact onepress@plos.org.

Kind regards,

Dinesh Kumar Nishad, PhD

Academic Editor

PLOS One

Additional Editor Comments (optional):

Reviewers' comments:

Reviewer's Responses to Questions

**Comments to the Author**

1. If the authors have adequately addressed your comments raised in a previous round of review and you feel that this manuscript is now acceptable for publication, you may indicate that here to bypass the “Comments to the Author” section, enter your conflict of interest statement in the “Confidential to Editor” section, and submit your "Accept" recommendation.

Reviewer #5: All comments have been addressed

**********

2. Is the manuscript technically sound, and do the data support the conclusions?

The manuscript must describe a technically sound piece of scientific research with data that supports the conclusions. Experiments must have been conducted rigorously, with appropriate controls, replication, and sample sizes. The conclusions must be drawn appropriately based on the data presented.

Reviewer #5: Yes

**********

3. Has the statistical analysis been performed appropriately and rigorously?

Reviewer #5: Yes

**********

4. Have the authors made all data underlying the findings in their manuscript fully available?

The PLOS Data policy requires authors to make all data underlying the findings described in their manuscript fully available without restriction, with rare exception (please refer to the Data Availability Statement in the manuscript PDF file). The data should be provided as part of the manuscript or its supporting information, or deposited to a public repository. For example, in addition to summary statistics, the data points behind means, medians and variance measures should be available. If there are restrictions on publicly sharing data—e.g. participant privacy or use of data from a third party—those must be specified.

Reviewer #5: Yes

**********

5. Is the manuscript presented in an intelligible fashion and written in standard English?

PLOS ONE does not copyedit accepted manuscripts, so the language in submitted articles must be clear, correct, and unambiguous. Any typographical or grammatical errors should be corrected at revision, so please note any specific errors here.

Reviewer #5: Yes

**********

6. Review Comments to the Author

Please use the space provided to explain your answers to the questions above. You may also include additional comments for the author, including concerns about dual publication, research ethics, or publication ethics. (Please upload your review as an attachment if it exceeds 20,000 characters)

Reviewer #5: I would like to sincerely thank the authors for their careful and thoughtful revision of the manuscript. I read and evaluated this work with great interest and pleasure, and I appreciate the effort the authors have made throughout the revision process.

In my opinion, the current version of the manuscript is much improved. The revised manuscript is clearer, more consistent, and of higher overall quality. The authors have satisfactorily addressed the previous comments, and the additional clarifications have strengthened both the methodological presentation and the interpretation of the results.

I only noticed a few very minor editorial/production issues that do not affect the scientific content: the submission metadata still appears to contain “A A letter…”, and some numerical formatting in Table 5 may still benefit from final editorial standardization. These are minor presentation issues and should not delay publication.

Overall, I believe that the manuscript is now suitable for publication in PLOS ONE. I congratulate the authors on their work and wish them continued success in their future research. I would also be pleased to see future high-quality contributions from the authors, including in PLOS ONE.

**********

7. PLOS authors have the option to publish the peer review history of their article (what does this mean?). If published, this will include your full peer review and any attached files.

If you choose “no”, your identity will remain anonymous but your review may still be made public.

**Do you want your identity to be public for this peer review?**  For information about this choice, including consent withdrawal, please see our Privacy Policy.

Reviewer #5: No

**********

# Editor report: Acceptance letter

**DOI:** 10.1371/journal.pone.0354512.r012

**Authors:** Dinesh Nishad

PONE-D-25-68879R5

PLOS One

Dear Dr. Samaras,

I'm pleased to inform you that your manuscript has been deemed suitable for publication in PLOS One. Congratulations! Your manuscript is now being handed over to our production team.

At this stage, our production department will prepare your paper for publication. This includes ensuring the following:

* All references, tables, and figures are properly cited

* All relevant supporting information is included in the manuscript submission,

* There are no issues that prevent the paper from being properly typeset

You will receive further instructions from the production team, including instructions on how to review your proof when it is ready. Please keep in mind that we are working through a large volume of accepted articles, so please give us a few days to review your paper and let you know the next and final steps.

Lastly, if your institution or institutions have a press office, please let them know about your upcoming paper now to help maximize its impact. If they'll be preparing press materials, please inform our press team within the next 48 hours. Your manuscript will remain under strict press embargo until 2 pm Eastern Time on the date of publication. For more information, please contact onepress@plos.org.

You will receive an invoice from PLOS for your publication fee after your manuscript has reached the completed accept phase. If you receive an email requesting payment before acceptance or for any other service, this may be a phishing scheme. Learn how to identify phishing emails and protect your accounts at https://explore.plos.org/phishing.

If we can help with anything else, please email us at customercare@plos.org.

Thank you for submitting your work to PLOS ONE and supporting open access.

Kind regards,

PLOS ONE Editorial Office Staff

on behalf of

Dr. Dinesh Kumar Nishad

Academic Editor

PLOS One
